# Tumorigenic role of Musashi-2 in aggressive mantle cell lymphoma

**DOI:** 10.1038/s41375-022-01776-x

**Published:** 2022-12-12

**Authors:** Marta Sureda-Gómez, Patricia Balsas, Marta-Leonor Rodríguez, Ferran Nadeu, Anna De Bolòs, Álvaro Eguileor, Marta Kulis, Giancarlo Castellano, Cristina López, Eva Giné, Santiago Demajo, Pedro Jares, José I. Martín-Subero, Silvia Beà, Elias Campo, Virginia Amador

**Affiliations:** 1 Institut d’Investigacions Biomèdiques August Pi i Sunyer (IDIBAPS), Barcelona, Spain; 2 Centro de Investigación Biomédica en Red de Cáncer (CIBERONC), Madrid, Spain; 3 Department of Hematology Hospital Clinic of Barcelona, University of Barcelona, Barcelona, Spain; 4 Institució Catalana de Recerca i Estudis Avançats (ICREA), Barcelona, Spain; 5 Hematopathology Section, Department of Pathology, Hospital Clínic of Barcelona, University of Barcelona, Barcelona, Spain

**Keywords:** B-cell lymphoma, Haematopoietic stem cells

## Abstract

SOX11 overexpression has been associated with aggressive behavior of mantle cell lymphomas (MCL). SOX11 is overexpressed in embryonic and cancer stem cells (CSC) of some tumors. Although CSC have been isolated from primary MCL, their relationship to SOX11 expression and contribution to MCL pathogenesis and clinical evolution remain unknown. Here, we observed enrichment in leukemic and hematopoietic stem cells gene signatures in SOX11+ compared to SOX11– MCL primary cases. Musashi-2 (MSI2) emerged as one of the most significant upregulated stem cell-related genes in SOX11+ MCLs. SOX11 is directly bound to the *MSI2* promoter upregulating its expression in vitro. *MSI2* intronic enhancers were strongly activated in SOX11+ MCL cell lines and primary cases. MSI2 upregulation was significantly associated with poor overall survival independently of other high-risk features of MCL. MSI2 knockdown decreased the expression of genes related to apoptosis and stem cell features and significantly reduced clonogenic growth, tumor cell survival and chemoresistance in MCL cells. MSI2-knockdown cells had reduced tumorigenic engraftment into mice bone marrow and spleen compared to control cells in xenotransplanted mouse models. Our results suggest that MSI2 might play a key role in sustaining stemness and tumor cell survival, representing a possible novel target for therapeutic interventions in MCL.

## Introduction

Mantle cell lymphoma (MCL) is one of the most aggressive mature B-cell neoplasm, characterized by the t(11;14)(q13;q32) primary oncogenic event [1]. Two subgroups of the disease with distinct clinical, biological and molecular features have been described [2, 3]. Conventional MCL (cMCL) is characterized by lymph node involvement, short responses and frequent relapses with current therapies, and adverse outcome. On the other hand, leukemic non-nodal MCL (nnMCL) subtype frequently presents with peripheral blood (PB) involvement without adenopathies and longer survival, without requirement of treatment for long time [1, 4]. cMCL derives from naive-like B cell, whereas nnMCL evolves from a more differentiated, germinal center experienced, memory-like B cell [5–7]. SRY-related HMG-box gene 11 (SOX11) is aberrantly overexpressed in cMCL, and negative or weakly expressed in nnMCL [2, 4, 5, 8]. Several studies have shown the oncogenic role of SOX11 in MCL pathogenesis by blocking B-cell differentiation, activating BCR signaling, and promoting angiogenesis and a protective tumor microenvironment with immune evasive mechanisms [9–14].

MCL frequently responds to initial treatment, although later development of resistance is common, relapsing with more aggressive disease. Tumor chemoresistance has been attributed, in part, to the presence of cancer stem cells (CSC) in some tumors [15]. Although there is still no consensus on a CSC phenotype in MCL, several groups have isolated MCL-CSC using different markers [16–19], with self-renewal capacity, aldehyde dehydrogenase (ALDH) activity and clonogenicity, and increased tumorigenicity in vivo [16, 20–22]. Moreover, all isolated MCL-CSC have shown resistance to standard therapies that could explain why MCL is still an incurable lymphoma, despite adequate rate of complete remission to frontline treatments [23].

Several studies have highlighted the relevance of Sox family members regulating proliferation and differentiation of progenitor and stem cells [24, 25]. Sox2 along with transcription factors Oct4, Klf4 and c-Myc act as reprogramming factors during induced pluripotent stem cells generation [26]. Sox proteins activate self-renewal genes and repress differentiation genes, and also function as pioneers to poise genes for activation by a related Sox factor once differentiation ensues [27, 28]. SOX11 is expressed in CSC population of oligodendrogliomas [29] and enhances CSC properties, increasing ALDH activity, mammosphere formation and drug resistance in mammary cells [30]. Nevertheless, nothing is known about the possible stemness role of SOX11 in MCL.

Here, we have searched for stem cell-related genes in MCL and their possible relationship to SOX11 expression and contribution to MCL biological and clinical evolution.

## Materials and methods

### MCL cell lines and primary samples

Four SOX11+ MCL cell lines, Z138, Granta-519, JeKo-1 (ATCC CRL-3001, DSMZ ACC-342 and ATCC CRL-3006, respectively) and HBL-2 (kindly provided by Dr D. Colomer (Hospital Clinic, Barcelona, Spain)), one negative, JVM2 (ATCC CRL-3002), and JVM13 (ATCC CRL-3003) B-prolymphocytic leukemia cell line, were used for in vitro studies. HEK-293T cell line (ATCC CRL-3216) was used for lentivirus production and luciferase assays. MCL primary cases cryopreserved at the Hospital Clínic/IDIBAPS Biobank were used for RNA-seq (*n* = 12) (see the next section); activity of the stem cell marker ALDH on ex vivo experiments was analyzed in other primary cases (*n* = 8). The study was approved by the Institutional Review Board of Hospital Clínic.

### Gene expression and molecular profiling from MCL cases

The gene expression profile (GEP) of 54 leukemic purified MCL cases (GSE79196) (Supplementary Table S1) [31], was used for gene set enrichment analysis (GSEA), differential expression and survival analysis. Molecular characterization was performed as described [32].

We used the GEP of a validation series containing 39 MCL cases, previously published (EGAD00010001842) [32]. We performed RNA sequencing (RNA-seq) on 12 purified cells from MCL patients representative of the above cohorts (8 SOX11+ and 4 SOX11–) (Supplementary Table S2), integrating them with published RNA-seqs (2 SOX11+ and 2 SOX11– MCLs) from BLUEPRINT [7, 33].

### Epigenomic dataset from MCL cases

Reference epigenomes of MCL cases, cell lines and normal B cells were made out of chromatin states, chromatin accessibility (ATAC-seq), whole-genome DNA methylation, and gene expression (RNA-seq). Data were generated and processed as described [7, 33, 34].

### Microarray analyses

Pre-published microarray data (GSE79196) (EGAD00010001842) were preprocessed by fRMA or RMA. Differentially expressed genes (DEG) were obtained with limma package. Genes with adjusted *P* value <0.05 and absolute log_2_ fold change >0.7 were selected.

### RNA-seq

RNA-seq libraries were sequenced on a HiSeq2500 (Supplementary Table S2). Sequencing reads were pseudo-aligned to GRCh38.p13-genome with kallisto. Differential expression was conducted using DESeq2. Genes were considered as differentially expressed when adjusted *P* value <0.1 and absolute log2-transformed fold change >0.65.

### Plasmid generation

Guide RNA (gRNA) for SOX11 knockout (KO) (Supplementary Table S3) was cloned in pL-CRISPR.EFS.GFP plasmid (Addgene#57818). pCDH-MCS-T2A-Puro-MSCV-Flag-SOX11 plasmid [13], was used for SOX11 overexpression, and empty plasmid pCDH-MCS-T2A-Puro-MSCV (CD522A-1; System Bioscience) as control. MSI2 MISSION shRNA Plasmids (Sigma-Aldrich) (Supplementary Table S3) were used for MSI2 knockdown (MSI2KD), generating sh4MSI2 and sh5MSI2 cell lines, respectively. Scramble-shRNA lentiviral particles (Santa Cruz Biotechnology) were used as control (shCT). Z138sh5MSI2 and Z138shCT cells were stable transduced with pLV430G-oFL-T2A-eGFP plasmid expressing luciferase and green fluorescent proteins (GFP).

### Lentiviral transduction

HEK-293T cells were transfected with lentiviral packaging, envelope and expression plasmids (see Plasmid generation). Granta-519 and Z138 cells were transduced with concentrated lentivirus or commercial Control shRNA Lentiviral Particles (sc-108080; Santa Cruz Biotechnology) and selected with puromycin (Gibco) or G-418 (Sigma). For SOX11KO and generation of Z138shCT-Luc and Z138sh5MSI2-Luc cells, GFP+ cells were sorted in FACSAriaII Cell Sorter (BD).

### Luciferase assay

*MSI2* promoter region (chr17:57,257,388-57,257,837) was amplified by PCR (Supplementary Table S4) and cloned in pGL4.23 plasmid containing luciferase reporter gene. Reporter construct in cotransfection with SOX11 full-length (pcDNA3-HA-SOX11) or truncated SOX11 protein (pcDNA3-HA-SOX11ΔHMG) vectors was used for luciferase assay in HEK-293T cells, performed as described [12].

### RNA-immunoprecipitation

RNA-immunoprecipitation (RIP) was performed using Magna RIP RNA-binding protein immunoprecipitation kit (Millipore) following the manufacturer’s indications. Lysates from 30 × 10^6^ Z138 cells were incubated with magnetic beads protein A/G bound to 3 µg of anti-MSI2 or IgG antibodies (Supplementary Table S5) on a rotating wheel overnight at 4 °C. RNA was extracted from protein-RNA-complexes with Phenol:Chloroform:Isoamyl Alcohol (125:24:1) after digestion with proteinase K. cDNA was generated using Verso cDNA synthesis kit (Thermo Scientific) and specific primers (Supplementary Table S4) were used for RT-qPCR.

### Colony assay

For colony assay, 500 growing cells were mixed with Human Methylcellulose Complete Media (R&D System). For MSI2 in vitro inhibition, cells were pre-treated with Ro 08-2750 (Tocris Bioscience) inhibitor [35], or DMSO.

### Surface and intracellular antigens for flow cytometry

MCL cells treated with Ro 08-2750 (Ro), Doxorubicin (Selleckchem) or untreated were incubated with Annexin Binding Buffer mixed with Propidium Iodide (PI) and/or Annexin V-FITC (eBioscience) to analyze apoptosis; fixed and stained with anti-active caspase 3 antibody, or directly stained with anti-Fas antibody or isotype to detect antigens by flow cytometry (FC) (Supplementary Table S5). For cell cycle analysis, Click-iT Plus EdU Pacific Blue FC Assay Kit (Thermo Fisher Scientific) was used, following instructions. Cells were analyzed by FC.

### Cytotoxicity assay

MCL and lymphoblastic cell lines treated with increasing concentrations of Ro were incubated with MTT (Invitrogen) and formazan crystals were solubilized. Absorbance was quantified in Sinergy HT spectrophotometer.

### RT-qPCR

RNA from MCL leukemic primary cases and cell lines was extracted using RNeasy Plus kit (QIAGEN). cDNA was generated using qScript cDNA Synthesis Kit (Quantabio) and analyzed by RT-qPCR using Fast SYBR Green Master Mix or TaqMan FAST Universal PCR Master Mix (Applied Biosystems), and primers or probes (Supplementary Table S4).

### Western blot

Protein was separated by SDS–PAGE and transferred to nitrocellulose membranes. Membranes were blocked for 1 h and incubated overnight with primary antibodies (Supplementary Table S5). Next, 1 h of incubation with a secondary antibody (Supplementary Table S5) was done. Pierce ECL reagent (Thermo Fisher Scientific) was used to detect proteins in ImageQuant LAS4000 (Fujifilm). Protein quantification was performed with MultiGauge Software (Fujifilm).

### ALDEFLUOR assay

MCL primary cells were treated with Ro or DMSO 0.05% for 24 h. ALDEFLUOR assay kit (STEMCELL Technologies) was used following the manufacturer’s recommendations.

### Engraftment in xenograft mice models

Immunodeficient NSG mice (NOD.Cg-Prkdc^scid^Il2rg^tm1Wjl^/SzJ, Janvier-LABS) were intravenously injected with Z138sh5MSI2-Luc or Z138shCT-Luc cell lines (5 mice/group), generating MSI2CT-Luc+ and MSI2KD-Luc+ xenografts. Mice were euthanized at 35 days post-inoculation and percentage of MCL cells was determined by FC.

### Statistics

Unpaired two-tailed Student’s *t*-test was used for comparisons between groups. Overall survival (OS) was used for Kaplan–Meier curves. Cox regression was used to evaluate prognosis. *P* values were adjusted with Benjamini and Hochberg method. EC50 was obtained fitting dose–response curve with non-linear regression methods. Statistical tests were performed using R (v3.6.9) or GraphPad Prism 5.

See Supplementary methods for additional information on all sections.

## Results

### MSI2 is upregulated in SOX11+ MCLs and associates with shorter overall survival in MCL primary cases

**Fig. 1:**
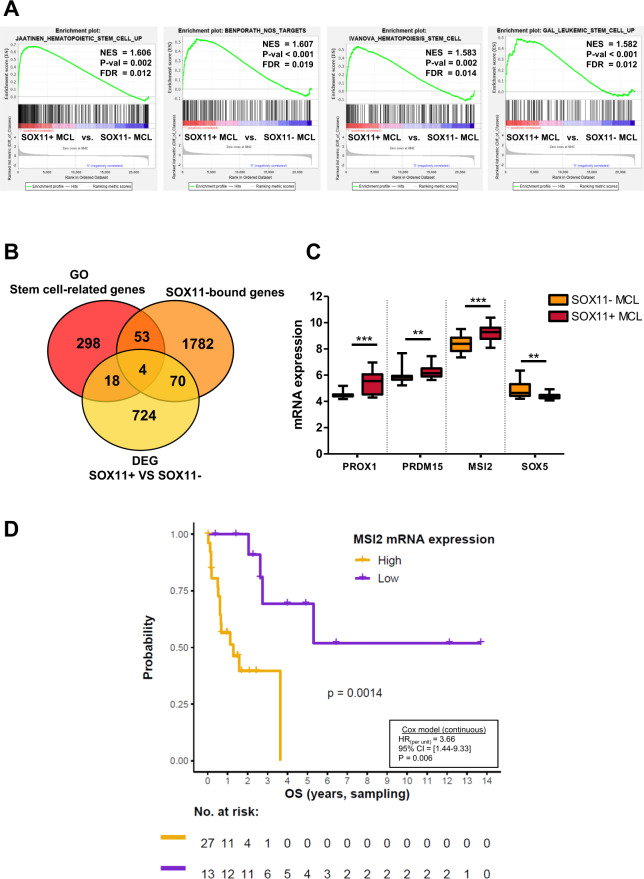
MSI2 expression is upregulated in SOX11+ compared to SOX11– MCL cases and associated with poor survival in MCL patients. **A** Enrichment plots obtained by GSEA using GEP microarray data from 30 SOX11+ and 24 SOX11– MCL primary tumors (GSE79196), showing significant enrichment of HSC, LSC and NOS-related target gene sets in SOX11^+^. Normalized enrichment score (NES), *P* value (*P* val) and false discovery rate (FDR) are shown. Statistical significance is considered when FDR < 0.2. **B** Venn diagram illustrating the overlap between differential expressed genes (DEG) between SOX11+ and SOX11– MCL primary cases (GSE79196) (yellow circle, 816 genes with adjusted *P* value <0.05 and absolute log_2_ fold change >0.7; see Supplementary Table S6), genes whose gene ontology biological process definition is related to stem cells (GO stem cell-related genes) (red circle, 373 genes; see Supplementary Table S7) and SOX11-bound genes (GSE35021) (orange circle, 1909 genes found by SOX11-specific ChIP-chip in MCL cell lines). **C** mRNA expression levels of *PROX1*, *PRDM15*, *MSI2* and *SOX5* genes in 30 SOX11+ and 24 SOX11– MCL primary cases (GSE79196). Expression levels were calculated by average of all the microarray probes of each gene, except for *MSI2* (see methods). The significance of difference was determined by unpaired two-tailed Student’s *t*-test (Welch’s correction was used for SOX5): **P value <0.01, ***P value <0.001. **D** Kaplan–Meier curve showing the association of MSI2 mRNA expression and OS in 40 MCL primary cases (GSE79196) (Supplementary Table S1). MSI2 high and low values were defined by maximally selected rank statistics (cutoff = 8.46 expression units). The *P* value of log-rank test (*P*), the risk table (no. at risk), the hazard ratio (HR) with 95% confidence interval (CI) and Cox regression *P* value (*P*) are shown.

To study the possible relationship of SOX11 expression and stemness properties in MCL, we analyzed the expression of stem cell-related gene signatures comparing the GEP of leukemic cells from 30 SOX11+/cMCL and 24 SOX11–/nnMCL primary cases (GSE79196) [31] (Supplementary Table S1). By GSEA, we observed a significant enrichment of hematopoietic stem cell (HSC), NANOG, OCT4 and SOX2 (NOS)-targets and leukemic stem cell (LSC) gene sets in SOX11+ compared to SOX11– MCLs (Fig. 1A and Supplementary Fig. S1). The enrichment of HSC, LSC and NOS gene sets in SOX11+ MCLs was validated in the GEP of an independent cohort of MCL (26 SOX11+ and 13 SOX11–) (EGAD00010001842) [32] (Supplementary Fig. S2A). Moreover, we performed RNA-seq on 12 samples and integrated them with 4 pre-published RNA-seqs data from BLUEPRINT (10 SOX11+ and 6 SOX11–, in total), to confirm the enrichment of stem cell-related gene sets in SOX11+ MCLs (Supplementary Fig. S2B).

To identify genes involved in stem cell features directly regulated by SOX11, we overlapped DEG between SOX11+ and SOX11– primary MCLs [31] (Supplementary Table S6) with established gene ontology (GO) functional stem cell-related genes (Supplementary Table S7) and SOX11-specific ChIP-chip bound genes in MCL cell lines [9]. This analysis showed that 74 out of the 816 DEG (9%) overlapped with SOX11-bound genes; 4 of them with experimentally validated stemness functions (Fig. 1B). We observed that the expression of *Prospero Homeobox 1 (PROX1), PR/SET Domain 15 (PRDM15)* and *Musashi-2 (MSI2)* genes were significantly upregulated and *SOX5* downregulated, in SOX11+ compared to SOX11– MCLs (Fig. 1C). Significant differences in PROX1, MSI2 and SOX5 mRNA levels between SOX11+ and SOX11– MCLs were also found in our validation cohorts, by microarray and RNA-seq (Supplementary Fig. S2C, D, respectively). These results suggest that SOX11 might directly regulate the transcription of these genes, activating the expression of *PROX1* and *MSI2* and repressing *SOX5* genes in MCL.

We next evaluated the clinical impact of the upregulated genes, using the GEP, molecular and OS data of MCL patients, in our initial series (Supplementary Table S1) [31]. We observed that higher MSI2 mRNA levels were significantly associated with shorter OS of patients (Fig. 1D), but not PROX1 levels (data not shown). In bivariate COX regression analyses, the adverse OS prognostic value of MSI2 was independent of different high-risk MCL features, such as SOX11 expression, high copy number alterations (≥6 CNA), *TP53* (17p13.1) and *CDKN2A* (9p21.3) alterations (Table 1).

**Table 1:** Bivariate Cox regression analysis for OS in 40 leukemic MCL cases (GSE79196), using MSI2 mRNA expression levels (continuous) and different molecular prognostic factors (SOX11, high CNA, and *TP53* and *CDKN2A* alterations) (categorical).

	HR	95% CI	*P* value
MSI2 mRNA expression	2.85	[1.07–7.57]	0.036
SOX11 status	3.42	[1.03–11.35]	0.044
MSI2 mRNA expression	4.09	[1.44–11.60]	0.008
CNA (High >6 CNA)	1.03	[0.37–2.85]	0.950
MSI2 mRNA expression	4.16	[1.54–11.21]	0.005
*TP53* (17p13.1)	1.86	[0.73–4.76]	0.192
MSI2 mRNA expression	4.96	[1.59–15.44]	0.006
*CDKN2A* (9p21.3)	7.92	[1.99–31.52]	0.003

*HR* hazard ratio, *CI* confidence interval, *P value* Cox regression *P* value.

Together, these results suggest that MSI2 may be a prognostic factor with a tumorigenic role in aggressive MCL.

### SOX11 upregulates MSI2 by direct binding to its promoter in aggressive MCL

**Fig. 2:**
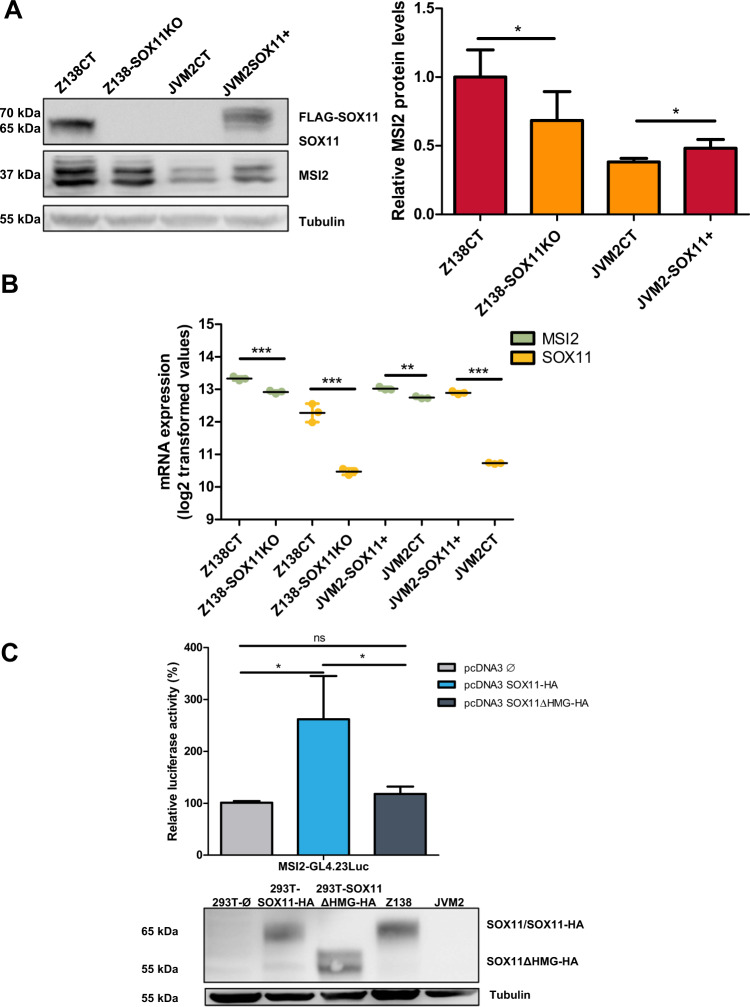
MSI2 is upregulated by SOX11 in MCL. **A** Left: MSI2 and SOX11 protein levels obtained by western blot in Z138 SOX11 knockout (Z138-SOX11KO), Z138 control (Z138CT), JVM2 control (JVM2CT) and JVM2 ectopically overexpressing SOX11 (JVM2-SOX11+, FLAG-SOX11 tagged protein) MCL cell lines. Tubulin protein levels were used as loading control. Right: quantification of MSI2 protein levels relative to their tubulin (in fold change) and normalized to Z138CT in 5 independent western blot experiments. **B** MSI2 and SOX11 mRNA levels (log_2_-transformed values) in Z138CT, Z138-SOX11KO, JVM2CT and JVM2-SOX11+ MCL cell lines, using RNA-seq data. **C** Top: luciferase assay in transient co-transfections of MSI2 promoter region-GL4.23 Luc with SOX11 full-length (pcDNA3 SOX11-HA) or the truncated SOX11 proteins (pcDNA3 SOX11∆HMG-HA) in HEK-293T cell line. Results are shown as fold induction percentage referred to luciferase activity in cotransfection with empty vector (pcDNA3ϕ) in two independent experiments. Bottom: SOX11 protein levels in HEK-293T pcDNA3ϕ, SOX11-HA and SOX11∆HMG-HA cells after 48 h of transfection, obtained by western blot. Tubulin was used as loading control and Z138 and JVM2 cell lines were used as positive and negative control of SOX11 expression, respectively. The significance of differences was determined by unpaired two-tailed Student’s *t*-test in panels **A**–**C**: *P value <0.05, **P value <0.01, ***P value < 0.001, ns: not significant.

To analyze whether SOX11 directly regulates MSI2 expression in MCL, we first knocked out (KO) SOX11 in Z138 SOX11+ MCL cell line (Z138-SOX11KO). In addition, SOX11 was overexpressed in the SOX11– JVM2 MCL cell line (JVM2-SOX11+) [13]. MSI2 protein levels were reduced in Z138-SOX11KO and increased in JVM2-SOX11+ compared to their corresponding controls (Z138CT and JVM2CT, respectively) (Fig. 2A). The modulation of MSI2 by SOX11 was also confirmed by RNA-seq, showing significant lower mRNA levels in Z138-SOX11KO and JVM2CT compared to Z138CT and JVM2-SOX11+ cells, respectively (Fig. 2B). Besides, MSI2 protein and mRNA levels were significantly recovered upon SOX11 ectopic overexpression in Z138-SOX11KO cell line (Z138-SOX11KO SOX11+) (Supplementary Fig. S3A, B, respectively), indicating the implication of SOX11 in the transcriptional upregulation of MSI2 in MCL cells.

Z138CT and JVM2-SOX11+ presented a GEP enriched in stem cell-related gene signatures compared to Z138-SOX11KO and JVM2CT cells, respectively (Supplementary Fig. S4), suggesting that SOX11 regulates MSI2 and other stemness factors in MCL.

In a previous study, we observed that SOX11 binds directly to *MSI2* promoter region, by ChIP-chip and ChIP-qPCR experiments [9]. To confirm that this binding regulates *MSI2* transcription, we performed luciferase assays in HEK-293T cells and observed activity when the *MSI2* promoter cloned in front of a minimal luciferase reporter was transiently co-transfected with a vector expressing SOX11-HA full-length, but not with SOX11 lacking the high mobility group domain (SOX11ΔHMG-HA) (Fig. 2C). Together, these results support that SOX11 directly binds *MSI2* promoter and activates its transcription.

### *MSI2* intronic superenhancers associate with MSI2 upregulation and SOX11 expression in MCL

**Fig. 3:**
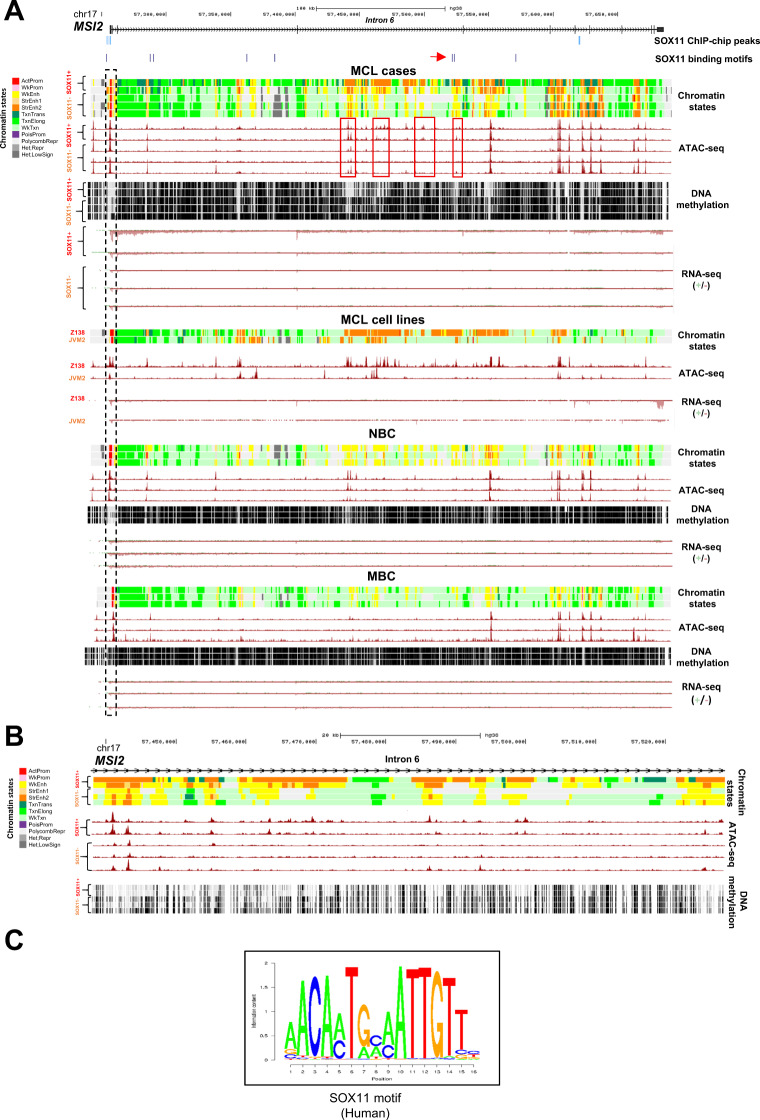
*MSI2* intronic superenhancers associate with *MSI2* upregulation and *SOX11* expression in MCL. **A** Multiple epigenetic layers of *MSI2* gene region (GRCh38/hg38 version, chr17:57,240,242-57,691,985) and gene expression in MCL primary cases (2 SOX11+ and 3 SOX11–), MCL cell lines (Z138 and JVM2), naive B cells (NBC) and memory B cells (MBC), generated in the BLUEPRINT consortium, which includes different histone modification marks (H3K4me3, H3K27ac, H3K4me1, H3K36me3, H3K9me3, H3K27me3) by ChIP-seq, used to generate the chromatin states; chromatin accessibility by ATAC-seq; DNA methylation by whole-genome bisulfite sequencing and gene expression by RNA-seq. SOX11-binding motifs were obtained with PWMScan, using SOX11 human motif from Hocomoco v11 Human TF Collection (**C**), and two of the motifs found are highlighted with a red arrow. SOX11-binding regions (SOX11 ChIP-chip peaks) were obtained by SOX11-specific ChIP-chip experiment in MCL cell lines (GSE35021). Chromatin states indicated by different colors (upper-left legend), ATAC-seq (signal from 0 to 40), DNA methylation (signal from 0 to 1) and RNA-seq (signal for positive strand from 0 to 5 and for negative strand from –5 to 0) are shown. Promoter region is underline with a black dashed rectangle. Specific SOX11+ MCL ATAC-seq peaks are highlighted with red rectangles. **B** Maximization of region chr17:57,438,165-57,527,952 (GRCh38/hg38 version) showing enhancer regions, ATAC-seq peaks (signal from 0 to 40) and DNA methylation (signal from 0 to 1) on *MSI2* intron 6 in 2 SOX11+ and 3 SOX11– MCL cases. Chromatin states are indicated by different colors (legend). **C** SOX11-specific consensus binding motif in *Homo sapiens* extracted from Hocomoco v11 collection.

To have a more comprehensive understanding of the mechanisms underlying MSI2 upregulation, we analyzed the epigenetic profile of the *MSI2* locus using reference epigenomes, comparing two SOX11+ and three SOX11– MCL cases, Z138 (SOX11+) and JVM2 (SOX11–) MCL cell lines, and naive (NBC) and memory B cells (MBC) [7, 33, 34]. The *MSI2* promoter showed open chromatin and active marks in SOX11+ and SOX11– MCL cases, cell lines, and normal B cells (Fig. 3A, black dashed rectangle). Furthermore, SOX11+ cases and cell line (Z138) had strong active enhancers located in intronic regions associated with higher chromatin accessibility, DNA hypomethylation and MSI2 expression (RNA-seq; (-) strand) compared to SOX11– MCLs, JVM2 and normal B cells (Fig. 3A, B).

SOX11-specific binding motifs (Fig. 3C) were found in *MSI2* promoter, near the SOX11-binding region previously found by ChIP-chip in MCL cells [9], and also in an enhancer region located on intron 6 (Fig. 3A, red arrow). Several matches to Sox family binding motifs were observed by FIMO analysis in specific SOX11+ MCL ATAC-seq peaks (Supplementary Table S8 and Fig. 3A, red rectangles).

These data support that *MSI2* expression associates with several active *MSI2* intronic superenhancers, only strongly activated in SOX11+ MCL.

### MSI2 promotes clonogenic growth, tumor cell survival and chemoresistance in MCL

**Fig. 4:**
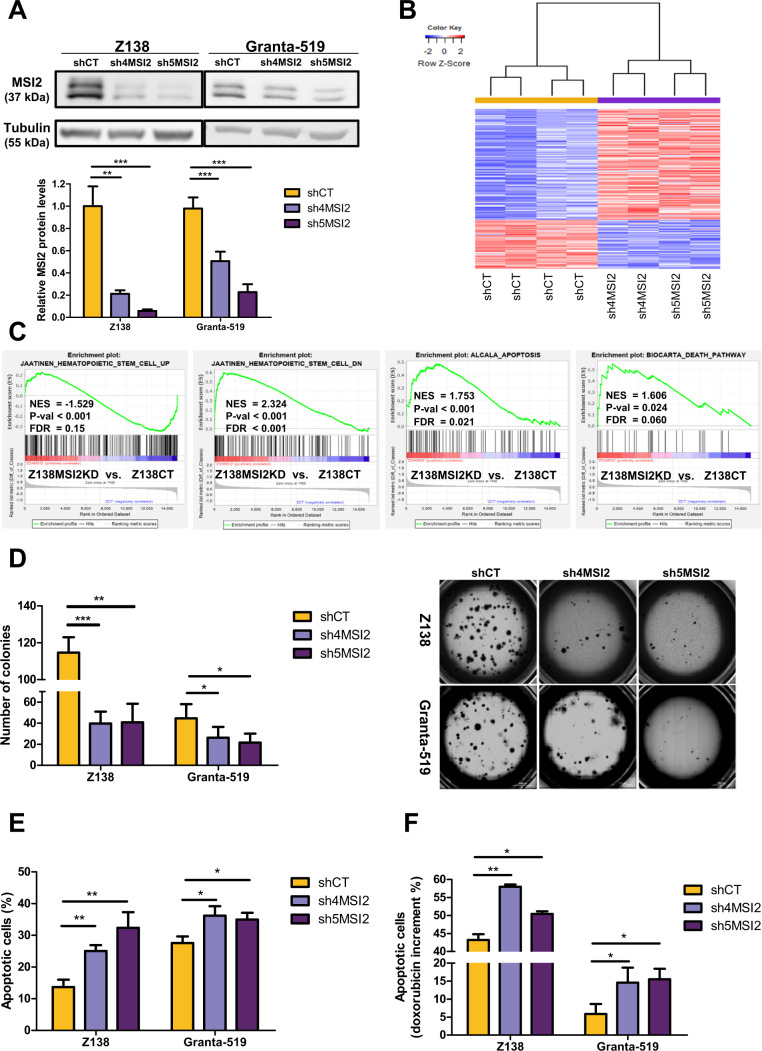
MSI2 knockdown downregulates HSC– while upregulates apoptotic-related gene sets and decreases clonogenic growth, cell survival and doxorubicin chemoresistance in MCL cell lines. **A** Top: western blot showing MSI2 protein levels upon MSI2KD in Z138 and Granta-519 (sh4MSI2 and sh5MSI2) compared to control (shCT) MCL cell lines, stable transduced with a scramble-shRNA lentiviral vector. Tubulin was used as a loading control. Bottom: quantification of MSI2 protein levels relative to their tubulin (in fold change) and normalized to Z138 or Granta-519 shCT, respectively, from 3 independent western blot experiments. **B** Heatmap illustrating the scaled expression (*Z*-score) of 401 DEG (277 upregulated and 124 downregulated genes; Supplementary Table S9) in Z138shCT compared to sh4MSI2 and sh5MSI2 Z138 cell lines, obtained by RNA-seq. Samples are shown in columns (shCT in yellow and shMSI2 in purple) and genes in rows; red indicates high expression and blue low. Genes with an adjusted *P* value <0.1 and absolute log_2_-transformed fold change >0.65 were considered. **C** GSEA on GEP RNA-seq data from Z138MSI2KD vs. Z138CT cell lines using gene sets related to HSC and apoptosis. NES, *P* val and FDR are shown, and statistical significance is assumed when FDR < 0.2. **D** Left: bar graph representing the number of colonies counted after 2 weeks of Z138 and Granta-519 shCT and shMSI2 cells growing in methylcellulose (1 colony >50 cells). Right: bright-field images of colony assay were obtained using Cytation 5 Imaging Reader with 4X objective lens. To create the full picture 88 images were stitched together (see Methods). **E** Bar graph representing the percentage of Annexin V+ population for shCT and shMSI2 Z138 and Granta-519 cells. **F** Percentage of increment in apoptotic cells (% Doxorubicin Annexin V+ cells – % Basal Annexin V+ cells) after 24 h of Doxorubicin treatment in Z138 and Granta-519 shCT and shMSI2 cells (0.05 and 3 µM for Z138 and Granta-519 cell lines, respectively). Results of panels **A**, **D**, **E** and **F** are represented as the mean ± standard deviation of at least 3 independent experiments. The significance of the difference was determined by unpaired two-tailed Student’s *t*-test: *P value <0.05, **P value <0.01, ***P value <0.001.

To elucidate the stemness role of MSI2 in MCL, we first silenced MSI2 in Z138 and Granta-519 MCL cell lines. Two shRNAs (sh4MSI2 and sh5MSI2) efficiently reduced MSI2 protein levels compared to control cells (shCT) (Fig. 4A). We then analyzed by RNA-seq the GEP upon MSI2 knockdown (MSI2KD) and found 277 upregulated and 124 downregulated genes compared to Z138shCT (Fig. 4B, Supplementary Fig. S5A and Supplementary Table S9). Z138shCT (Z138CT) cell lines were enriched in genes upregulated in HSC (UP); while Z138sh4MSI2 and sh5MSI2 (Z138MSI2KD) were enriched in genes downregulated in HSC (DN) and in gene sets related to apoptosis (Fig. 4C). Moreover, we observed that downregulated genes upon MSI2KD were involved in regulating pluripotency of stem cells, NOTCH, TP53 and WNT signaling pathways. On the contrary, the upregulated genes upon MSI2KD were involved in proliferation and apoptotic pathways (Supplementary Fig. S5B).

CDK6 and NOTCH1, important genes in stemness [36–38] and MCL pathogenesis [39, 40], were identified as MSI2-direct targets in long-term hematopoietic stem cells (LT-HSCs) [41] and possible targets in MCL, as they were differentially expressed upon MSI2KD in Z138 cell line (Supplementary Fig. S6A). We validated the direct binding of MSI2 to CDK6 and NOTCH1 mRNAs by RNA-Immunoprecipitation (RIP)-RT-qPCR experiments in Z138 MCL cell line (Supplementary Fig. S6B). CDK6 and NOTCH1, but not control GUSB, mRNAs were significantly enriched over their inputs in RIPs performed with specific anti-MSI2 antibody, but not with control anti-IgG antibody (Supplementary Fig. S6C). In line with MSI2 RNA-binding activity regulating protein translation [35, 42–44], CDK6 and NOTCH1 protein levels were significantly reduced upon MSI2KD (sh4MSI2 and sh5MSI2), compared to Z138CT cells (Supplementary Fig. S6D–F).

Colony formation was significantly reduced upon MSI2KD in both Z138 and Granta-519-sh4MSI2 and -sh5MSI2 compared to shCT cells (Fig. 4D). Interestingly, SOX11KO in Z138 cell line downregulated MSI2 and significantly reduced colony growth compared to control cells. These results suggest that SOX11 might be regulating MCL cell self-renewal through MSI2. However, depletion of MSI2 by shRNA in SOX11KO showed a significantly higher decrease in the number of colonies formed compared to SOX11KO alone in Z138 MCL cells, suggesting that other factors might also contribute in MSI2 function on the regulation of self-renewal in MCL (Supplementary Fig. S7). Furthermore, basal apoptosis (Fig. 4E and Supplementary Fig. S8A) and apoptosis induced by doxorubicin chemotherapy treatment significantly increased upon MSI2KD in both cell lines (Fig. 4F and Supplementary Fig. S8B). We observed mRNA upregulation of several caspases (CASP1, CASP10 and CASP8), genes related to mitochondrial apoptosis (*BAX* and *BLK*) and to extrinsic apoptosis (*FAS* and *PRF1*) (Supplementary Fig. S9A, B). In line, we observed, by FC, increased number of cleaved caspase 3+ cells and a higher FAS protein level in MSI2KD than in CT MCL cells (Supplementary Fig. S9C, D, respectively). Interestingly, ectopic overexpression of MSI2 in Z138MSI2KD cells rescued them from apoptosis (Supplementary Fig. S9E, F) and the percentage of cleaved caspase 3+ cells and FAS protein levels decreased, reaching the same levels as in Z138CT cells (Supplementary Fig. S9G, H). On the contrary, we did not observe changes in proliferation and cell cycle checkpoints upon MSI2KD in Z138 and Granta-519 cells (Supplementary Fig. S10A, B, respectively).

Together, these results suggest that MSI2 regulates the expression of HSC and pluripotency-related programs, mediates colony growth and chemoresistance; and inhibits apoptosis through downregulation of several pro-apoptotic gene pathways in MCL.

### MSI2 inhibition with Ro 08-2750 small molecule reduces stemness in MCL

Ro 08-2750 (Ro) is a small molecule that binds selectively to the MSI2 RNA-binding site, leading to MSI2 loss of function and affecting the survival of acute myeloid leukemia (AML) and chronic lymphocytic leukemia (CLL) cells [35, 45].

**Fig. 5:**
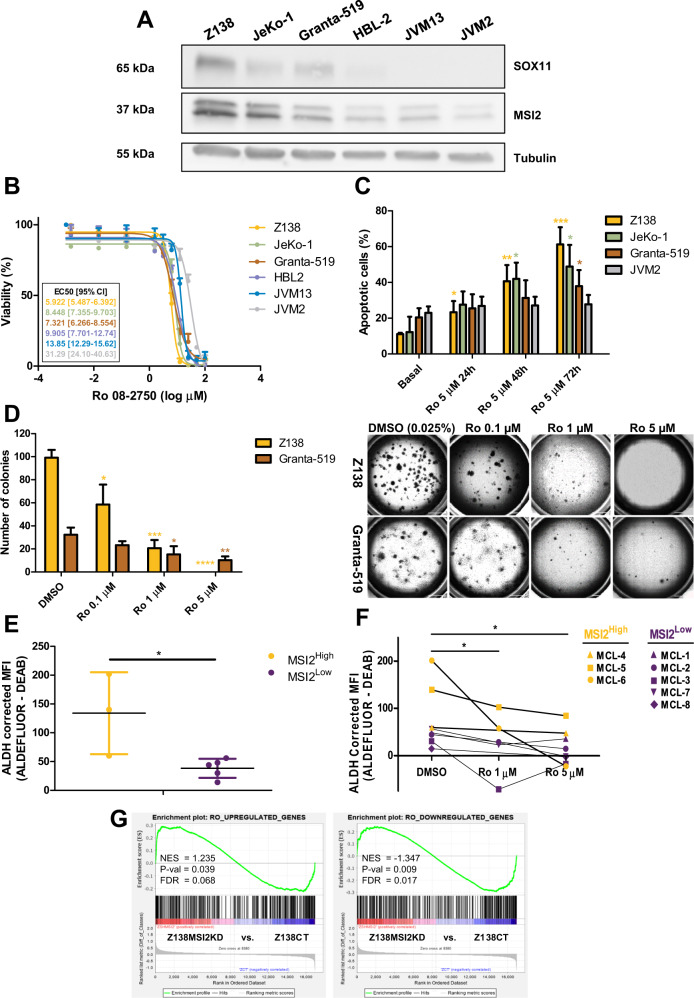
Ro 08-2750 small molecule inhibits MSI2 activity, impairing stemness functions in MCL. **A** Western blot experiments showing SOX11 and MSI2 protein levels in Z138, JeKo-1, Granta-519, HBL-2 and JVM2 MCL or JVM13 lymphoblastic cell lines. Tubulin was used as loading control. **B** Cytotoxicity of Ro in Z138, JeKo-1, Granta-519, HBL-2 and JVM2 MCL or JVM13 lymphoblastic cell lines, with decreasing MSI2 protein levels, by MTT assay. Data are represented as percentage of viability at different Ro concentrations (in log µM), relative to untreated cells. EC50 and confidence intervals (mean of two independent experiments) for each cell line are shown. **C** Apoptosis assay showing percentage of Annexin V+ cells in Z138, JeKo-1 and Granta-519 SOX11+, and JVM2-SOX11– MCL cell lines after 24, 48 and 72 h of Ro treatment (5 µM) or without treatment (basal). Statistical significance is determined by unpaired two-tailed Student’s *t*-test comparing to basal apoptosis in at least 3 independent experiments (asterisks comparisons: Z138 in yellow, JeKo-1 in green, Granta-519 in brown and JVM2 in gray). **D** Left: bar graph representing data for colony formation assays (number of colonies) in Z138 and Granta-519 cell lines treated at different concentrations of Ro drug (0.1, 1 and 5 µM) or with 0.025% of DMSO, used as control. Results are represented as the mean ± standard deviation of at least 3 independent experiments. Statistical significance is determined by unpaired two-tailed Student’s *t*-test compared to DMSO (asterisk comparisons: Z138 in yellow, Granta-519 in brown). Right: bright-field images of Z138 and Granta-519 colonies growing in Methylcellulose medium, obtained using Cytation 5 Imaging Reader with 4× objective lens, treated at different concentrations of Ro drug (0.1, 1 and 5 µM) or with 0.025% of DMSO. **E** ALDH activity quantified as corrected mean fluorescence intensity (MFI ALDEFLUOR – MFI ALDEFLUOR + DEAB) in MSI2^High^ (*N* = 3) and MSI2^Low^ (*N* = 5) MCL primary leukemic cases, measured by ALDEFLUOR assay. Results are represented as the mean ± standard deviation of all the cases in each group. The significance of difference was determined by unpaired two-tailed Student’s *t*-test. **F** ALDEFLUOR assay showing ALDH activity quantified as corrected mean fluorescence intensity (MFI ALDEFLUOR – MFI ALDEFLUOR + DEAB) in MSI2^Low^ (*N* = 5) and MSI2^High^ (*N* = 3) MCL primary leukemic cases, after 24 h of treatment with 1 and 5 µM of Ro or 0.05% of DMSO. The significance of difference was determined by paired one-tailed Student’s *t*-test. **G** GSEA on RNA-seq GEP data comparing Z138MSI2KD vs. Z138CT cell lines using gene sets upregulated and downregulated in Z138 cell line upon Ro treatments with 20 µM, compared to Z138 treated with 0.1% DMSO, obtained by differential expression analysis in RNA-seq data (Supplementary Table S10). NES, *P* val and FDR are shown, and statistical significance is assumed when FDR < 0.2. *P value <0.05, **P value <0.01, ***P value <0.001, ****P value <0.0001.

To determine if Ro could inhibit MSI2 activity in MCL, we first tested Ro cytotoxicity effects in MCL and lymphoblastic cell lines from high to very low MSI2 protein levels (Fig. 5A). We observed that increasing doses of Ro were needed to reduce the viability to 50% of different MCL and lymphoblastic cell lines with decreasing MSI2 levels (*EC*_50_ = 5.9, 7.3, 8.4, 9.9, 13.8 and 31.3 μM in Z138, Granta-519, Jeko-1, HBL-2, JVM13 and JVM2, respectively) (Fig. 5B). We found a qualitative inverse correlation between Ro cytotoxicity, measured by the logEC_50_, and the MSI2 protein levels in the cell lines (Supplementary Fig. S11A). We also found a significant increase in apoptosis upon Ro treatment at 5 μM in Z138, Granta-519 and Jeko-1 cells but not in JVM2 (Fig. 5C). Moreover, Ro treatment significantly reduced Z138 and Granta-519 colony formation (Fig. 5D), even at Ro concentrations (0.1 and 1 μM) that clearly did not induce apoptosis (Supplementary Fig. S11B).

We tested the activity of the stem cell marker ALDH in MCL primary samples expressing high and low levels of MSI2 (Supplementary Fig. S11C). ALDH activity was significantly higher in MSI2^High^ than in MSI2^Low^ cases (Fig. 5E). Besides, Ro treatment reduced ALDH activity in MCL primary cases (Fig. 5F and Supplementary Fig. S11D).

To globally assess the effect of Ro treatment in the MCL transcriptional program, we performed RNA-seq on Z138 cells after 4 h of treatment. Ro treatment altered gene expression, with 379 upregulated and 389 downregulated genes compared to Z138 treated with DMSO (Supplementary Table S10). GO analysis showed that upregulated genes upon Ro treatment were involved in apoptotic and TP53 signaling pathways, while the downregulated genes were involved in developmental signaling pathways (Supplementary Table S11). Interestingly, Z138MSI2KD cells were enriched in genes upregulated in Z138 Ro treated cells, while the downregulated genes upon Ro treatment were enriched in Z138CT compared to Z138MSI2KD cell lines (Fig. 5G). Moreover, CDK6 and NOTCH1 protein levels were significantly reduced when MCL cells were treated for 24 h with Ro compared to DMSO-treated cells (Supplementary Fig. S11E).

### MSI2KD delays MCL engraftment in vivo

**Fig. 6:**
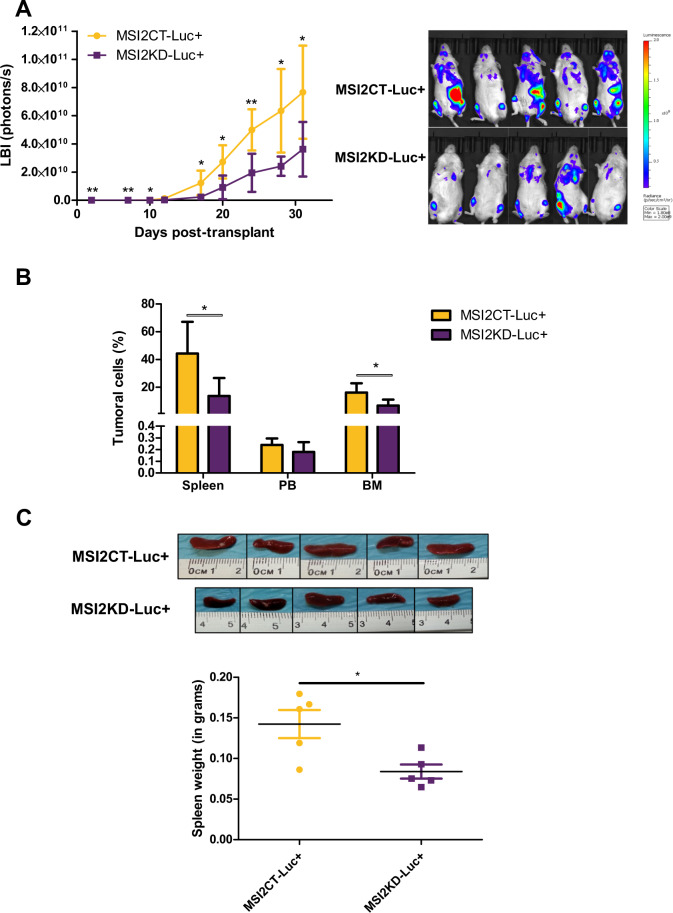
MSI2 knockdown reduces in vivo engraftment of MCL cells into mice BM and spleen of immunosuppressed MCL xenograft mouse models. **A** Left: NSG mice intravenously injected with 10 × 10^6^ Z138shCT (*n* = 5, MSI2CT-Luc+) or Z138sh5MSI2 (*n* = 5, MSI2KD-Luc+) cells expressing the GFP and Luciferase enzyme were imaged twice per week for 5 weeks. LBI signal (photons/s) shows the tumor engraftment at indicated days post tumor inoculation. LBI signal at mice ovaries was subtracted from the total signal for the two last points (days 28 and 31) to minimize the differences between males and females. Right: pictures showing the LBI signal in MSI2CT-Luc+ and MSI2KD-Luc+ MCL xenograft mice at day 31 post injection (luminescence signal from 1.00e8 to 2.00e9). **B** Bar graph displaying percentage of MCL tumoral cells in different mice tissues (SP spleen, PB peripheral blood, BM bone marrow), comparing MSI2CT-Luc+ and MSI2KD-Luc+ xenograft mice, analyzed using GFP fluorescence by FC. **C** Top: illustrative images of spleen engraftment in MSI2CT-Luc+ and MSI2KD-Luc+ MCL xenograft mice models. Bottom: graph showing spleen weight (in grams) in MSI2CT-Luc+ and MSI2KD-Luc+ xenograft mice models. Data are represented as the mean ± standard deviation of the 5 mice in panels **A**–**C**. The significance of difference was determined by unpaired two-tailed Student’s *t*-test: *P value <0.05, **P value <0.01.

To investigate the potential tumorigenic role of MSI2 in MCL in vivo, Z138sh5MSI2-Luc and Z138shCT-Luc cell lines were intravenously injected into immunodeficient NSG mice, generating MSI2KD-Luc+ and MSI2CT-Luc+ mouse models. We analyzed tumor engraftment every week monitoring luciferase bioimage (LBI) signal. Significant lower LBI signal was detected in MSI2KD-Luc+ compared to MSI2CT-Luc+ mice, already at day 2 post injection, showing significantly higher differences at the final time (Fig. 6A). Accordingly, 35 days after injection, MSI2CT-Luc+ had a higher number of MCL cells in the spleen and bone marrow (BM) (Fig. 6B) and significant higher spleen weight (Fig. 6C) than MSI2KD-Luc+ mice. However, no differences in PB were obtained.

These results suggest that MSI2 promotes tumorigenic growth into mice spleen and BM in MCL xenograft in vivo models and support the concept that targeting MSI2 in vivo could have therapeutic efficacy in MCL.

## Discussion

SOX11 is a neural transcription factor expressed in progenitors and embryonic stem cells (ESC) [46–49]. Its overexpression has been observed in undifferentiated tumor cells with CSC features [29, 30]. SOX11 regulates oncogenic factors in MCL and is aberrantly overexpressed in aggressive cMCLs [2, 4, 5]. To determine whether SOX11 could mediate stem-like properties in MCL, we compared the differential GEP between SOX11+ and SOX11– MCL cases and observed high enrichment of HSC and LSC-related genes in the SOX11+ subtype. In addition, SOX11KO in Z138 cells downregulated the expression of HSC and LSC-related gene signatures and reduced colony formation. These results suggest that SOX11 could be mediating stemness features in aggressive MCL, regulating the expression of stem cell-related genes, which could be associated with the chemotherapy resistance frequently observed in these tumors [2, 4].

*MSI2* emerged as one of the most significant stem cell-related genes upregulated in SOX11+ MCL primary cases. MSI2 mRNA and protein expression decreased upon SOX11KO and increased after SOX11 overexpression in MCL cell lines. Moreover, its expression was rescued upon SOX11 overexpression in Z138-SOX11KO cells, suggesting that SOX11 directly regulates MSI2 transcription in MCL cells. Besides, SOX11 binds to *MSI2* promoter increasing its expression. Nevertheless, additional factors must be involved in the regulation of *MSI2*, since SOX11– MCL patients express low levels of MSI2, and Z138-SOX11KO cells did not reach the low levels of MSI2 observed in the SOX11– MCL cell line, JVM2. Interestingly, we observed that MSI2 upregulation in SOX11+ MCL cells was associated with specific epigenetic changes compared to SOX11– MCLs or normal B cells, suggesting that active *MSI2* intronic enhancers may be responsible, in part, for MSI2 upregulation in MCL. However, further studies would be necessary to understand the complex regulation of MSI2 expression by epigenetics in MCL.

MSI2 regulates self-renewal and differentiation in ESC [50] and HSCs [51], and is overexpressed in solid [52, 53] and hematological malignancies [38, 43, 45, 51, 54]. High MSI2 expression has been associated with shorter survival in CML, myelodysplastic syndromes, CLL and AML [38, 45, 51, 54]. Interestingly, we observed that high MSI2 expression was associated with poor OS in MCL patients, independently of other high-risk factors, like SOX11 expression, high CNA, *TP53* and *CDKN2A* alterations.

MSI2 is an RNA-binding protein that mediates its biological function by controlling the translation of downstream oncogenic targets [43, 51]. MSI2 depletion or specific inhibition reduced colony formation, cell survival, leukemogenesis, self-renewal and proliferation followed by differentiation in several tumor cells [35, 38, 43, 45, 51, 54]. Interestingly, we observed that MSI2KD or MSI2 inhibition with Ro lead to GEP changes, downregulating genes involved in WNT, NOTCH and other developmental pathways whereas upregulating pro-apoptotic genes, in MCL cells. Concordantly, we observed direct binding of MSI2 to CDK6 and NOTCH1 mRNAs, reducing its protein levels upon MSI2KD or Ro treatment.

Cleaved Caspase 3 and FAS protein levels were also increased upon MSI2KD. Phenotype reverted upon MSI2 overexpression in MSI2KD MCL cells. MSI2KD in vitro suppressed stemness features, such as clonogenic growth, chemoresistance and tumor cell survival. SOX11KO reduced clonogenic growth. However, MSI2 silencing in SOX11KO cells significantly increased the reduction in colony formation compared to single SOX11KO, in Z138 cells. These results suggest that SOX11 is involved in stemness features principally through *MSI2* transcriptional regulation in MCL. In CLL, MSI2 promotes proliferation and cell survival [45]. However, upon MSI2KD we did not observe differences in proliferation or cell cycle, suggesting a different way of MSI2 action in MCL.

In line with other groups describing higher ALDH activity in MCL cell populations with clonogenicity [22], we observed higher ALDH activity in MSI2^High^ than in MSI2^Low^ MCL primary cases.

Interestingly, MSI2KD inhibited dissemination and growth of MCL cells into mice BM and spleen in vivo, suggesting that MSI2 is an important tumorigenic factor in MCLs.

**Fig. 7:**
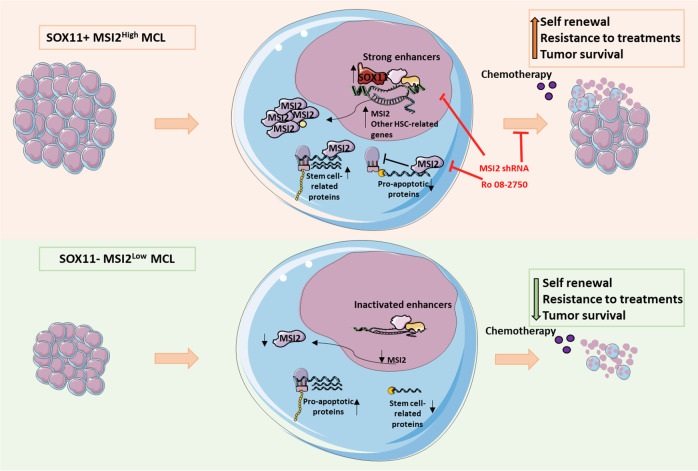
Model proposed for SOX11/MSI2 axis in the regulation of tumor self-renewal, cell survival, chemoresistance and tumorigenic growth in MCL. HSC- and LSC-related signatures are enriched and MSI2 is upregulated in SOX11+ MCL cases (upper panel) compared to negative MCL primary cases and cell lines (lower panel). MSI2 upregulation associates with activated *MSI2* intronic enhancers and SOX11 direct binding to its promoter, activating MSI2 expression in aggressive MCLs. MSI2 RNA-binding protein regulates developmental and cell death pathways, activating protein translation of stem cell-related proteins and blocking protein translation of pro-apoptotic transcripts in MCL. MSI2 expression increases in vitro clonogenic growth, cell survival and resistance to chemotherapy in MCL cells; and in vivo tumorigenic growth in MCL xenograft mice models. MSI2 depletion by shRNAs or MSI2 activity inhibition with Ro 08-2750 treatment can reverse stemness phenotypes in MCL, restoring the expression of pro-apoptotic proteins, downregulating stem cell-related transcripts, increasing apoptosis, drug sensitivity and decreasing colony formation. Overall, our results suggest that MSI2 is playing a key stemness role in MCL, and present MSI2 as a new potential therapeutic target for aggressive MCLs.

Overall, our findings suggest that MSI2 expression in MCL is regulated in part by SOX11 binding to its promoter and associated with active intronic superenhancers. MSI2 upregulation might contribute to the maintenance of stem cell properties in MCL cells through the post-transcriptional upregulation of stemness-related genes and downregulation of apoptotic factors, providing them with self-renewal capabilities, higher cell survival and chemoresistance (Fig. 7). Our results open a new perspective for treatment, highlighting MSI2 as potential therapeutic target to inhibit drug resistance and relapse in aggressive MCLs.

## Supplementary information

Supplementary data

## Data Availability

The RNA-seq data reported in this paper are available at the European Genome-phenome Archive (EGA) EGAS00001006613 number.
