# Supplementary material for: Tumorigenic role of Musashi-2 in aggressive mantle cell lymphoma
Source: Leukemia. 2022 Dec 12;37(2):408–21. doi: 10.1038/s41375-022-01776-x (PMC9898029; doi:10.1038/s41375-022-01776-x)
Supplement: Supplementary file 1 — Supplementary data [file 41375_2022_1776_MOESM1_ESM.pdf]

SUPPLEMENTARY DATA

TUMORIGENIC ROLE OF MUSASHI-2 IN AGGRESSIVE MANTLE CELL LYMPHOMA

Marta Sureda-Gómez[1], Patricia Balsas[1,2], Marta-Leonor Rodríguez[1], Ferran Nadeu[1,2], Anna De Bolòs[1], Álvaro Eguileor[1], Marta Kulis[1], Giancarlo Castellano[1], Cristina López[1,2], Eva Giné[1,2,3], Santiago Demajo[1], Pedro Jares[1,2], José I. Martín-Subero[1,2,4], Silvia Beà[1,2,5], Elias Campo[1,2,5], Virginia Amador[1,2]

1. Institut d'Investigacions Biomèdiques August Pi i Sunyer (IDIBAPS), Barcelona, Spain.

2. Centro de Investigación Biomédica en Red de Cáncer (CIBERONC), Madrid, Spain.

3. Department of Hematology Hospital Clinic of Barcelona, University of Barcelona, Barcelona, Spain.

4. Institució Catalana de Recerca i Estudis Avançats (ICREA), Barcelona, Spain

5. Hematopathology Section, Department of Pathology, Hospital Clínic of Barcelona, University of Barcelona, Barcelona, Spain.

SUPPLEMENTARY METHODS

MCL cell lines and primary samples

Z138 cell line was used for SOX11 knock out (KO) using CRISPR-Cas9 genome editing technology (see below). SOX11 expression was rescued in Z138-SOX11KO cell line by lentiviral transduction to obtain Z138-SOX11KO SOX11+ (FLAG-SOX11 tagged protein) cell line. We silenced MSI2 expression in Z138-SOX11KO cell line generating Z138-SOX11KO sh5MSI2 cell line. Z138 and Granta-519 wild type SOX11+ MCL cell lines were stable transduced to obtain MSI2 knockdown (KD) and control (CT) in vitro models, by lentiviral transduction, in which we performed functional in vitro analyses. MSI2 expression was rescued in Z138 sh4 and sh5MSI2 cells by lentiviral transduction to obtain Z138 sh4MSI2 and sh5MSI2 MSI2-FLAG cell lines. JVM2-SOX11+ (FLAG-SOX11 tagged protein) MCL cell line was previously obtained [1]. Transduced or wild type Z138, Granta-519, JeKo-1, HBL-2 and JVM2 MCL cell lines, and JVM13 B-prolymphocytic leukemia cell line, were used for clonogenic growth, apoptosis assays, cytotoxic treatment assays and in vitro experiments in normal conditions or after MSI2 inhibition with Ro 08-2750 small molecule. MSI2CT-Luc+ and MSI2KD-Luc+ cell lines were generated after lentiviral transduction of Z138shCT and sh5MSI2 cell lines with a lentiviral plasmid expressing the green fluorescence protein (GFP) and luciferase enzyme and used for in vivo experiments.

MCL primary cells from peripheral blood (PB) samples were isolated by Ficoll-Hypaque density gradient centrifugation (GE Healthcare). Some MCL primary cases (n=12) were used for RNA extraction and RNA-seq experiments (Table S2). Other MCL primary cases (n = 8) were used to test the activity of the stem cell marker ALDH on ex vivo experiments.

Cells were cultured at 37 ºC and 5% CO_2 in RPMI-1640 (Corning) (Z138, JeKo-1, JVM2 and primary samples) or DMEM (Lonza) (Granta-519 and HEK-293T) with L-glutamine supplemented with 10% FBS (Gibco), 100 µg/ml streptomycin and 100 U/ml penicillin (Gibco). Cell line authentication was performed by qCell Identity (qGenomics) and *Mycoplasma* contamination was regularly tested.

Cryopreserved MCL primary cells were obtained from the Hospital Clínic/IDIBAPS Biobank. The study was approved by the Institutional Review Board of the Hospital Clínic, Barcelona, Spain. Informed consent was obtained from all the subjects.

Gene expression and molecular profiling from MCL primary cases

Previously published GEPs of 54 leukemic purified (>95%) tumor cells from MCL primary cases (GSE79196) (Table S1) [2] were obtained by microarray analysis with the Affymetrix GeneChip Human Genome U133 Plus 2.0. For survival analysis, samples obtained after treatment (n=7) and from patients that received allogeneic stem cell transplant (n=2) were discarded. Follow-up information was missed in 5 patients. Previously published GEP data from lymph node or peripheral blood samples of 39 MCL primary cases, [3] was used for validation analysis.

MCL cases were classified as SOX11+ and SOX11- by immunohistochemistry and/or L-MCL16 assay [4,5]. To obtain MSI2 mRNA levels in Fig. 1C, 1D and Table 1 only 4 out of 10 probe sets, that showed a good expression and correlation (225237_s_at, 225238_at, 225240_at and 226134_s_at), were averaged together. To obtain mRNA levels for other genes analyzed in Fig. 1C and Fig. S2C, all the probe sets were averaged together.

Epigenomic dataset from MCL primary cases

Reference epigenomes of five MCL cases (2 SOX11+ and 3 SOX11-), 2 MCL cell lines (Z138 and JVM2), and sorted naïve B-cells and memory B-cells from 3 healthy donors were mined from previously published reports [6,7]. Chromatin states derived from ChIP-seq of six different histone modifications (H3K27ac, H3K4me1, H3K4me3, H3K36me3, H3K9me3, H3K27me3). The 12 chromatin states were generated at 200 bp interval using the chromHMM software [8], as previously described [9].

Motif enrichment analysis

PMWScan (https://ccg.epfl.ch/pwmtools/pwmscan.php) was used to scan SOX11 human motif from Hocomoco-v11 collection through human genome (GRCh38/hg38). FIMO analysis was performed on ATAC-seq peaks from Fig.3, using Sox family binding motifs from JASPAR database: ATAC_peak_1: chr17:57 440 795-57 441 325; ATAC_peak_2: chr17:57 463 187-57 463 603; ATAC_peak_3: chr17:57 499 225-57 500 470; ATAC_peak_4: chr17:57 525 240-57 527 912.

Microarray analyses

For differential expression analysis in microarray data, gene filtering was done with featureFilter in order to maintain only probes sets with Entrez Gene ID and with the highest interquartile range of the probe sets annotated by the same Entrez Gene ID. Differentially expressed genes (DEG) were obtained by linear models and empirical Bayes methods with limma package (3.42.2)

RNA-seq

RNA from MCL cell lines was extracted using RNeasy Plus kit (QIAGEN) following manufacturer's instructions. RNA from MCL primary cases was extracted with TRIZOL (Life Technologies). RNA quantity and quality were examined using NanoDrop (Thermo Scientific) and RNA 6000 Nano Assay on the Agilent 2100 Bioanalyzer (Agilent Technologies). RNA samples from cell lines or primary cases with RIN higher than 8 were selected to generate mRNA or total RNA libraries using the TruSeq RNA Sample Prep Kit v2 or the TruSeq Stranded Total RNA kit with Ribo-Zero Gold (Illumina), respectively, following manufacturer’s specifications. RNA-seq libraries were amplified, pooled and sequenced on a HiSeq2500 to generate 75 bp paired-end, 50 bp single-end or paired-end reads (Samples information, RNA extraction, library and sequencing conditions are shown in Table S2). More than 30 million of reads were sequenced for each sample. Sequencing reads were quality checked with FastQC (version 0.11.9), depleted from rRNA reads with SortMeRNA (version 4.3.2), trimmed to remove adapters and low-quality reads with trimmomatic (version 0.40), and pseudo-aligned to reference human genome GRCh38.p13 version with kallisto (version 0.46.1) to extract gene-level counts (Ensembl release 100) Gene level counts were imported to R (version 3.6.3, https://www.r-project.org) using tximport (version 1.12.3). Differential expression was conducted using DESeq2 package (version 1.24.0), shrinking the size factor with apeglm method. Variance stabilized expression matrix were obtained for gene set enrichment analysis (GSEA) and clustering. Lists of DEG were used for functional annotation analysis using DAVID (https://david.ncifcrf.gov/tools.jsp).

For Z138CT and Z138-SOX11KO or JVM2CT and JVM2-SOX11+, samples were sequenced per triplicated. For MSI2 silencing, 4 samples from Z138shCT, 2 from Z138sh4MSI2 and 2 from Z138sh5MSI2 were sequenced. For Ro inhibitory experiments,

Z138 cell line was independently treated per triplicated with Ro 08-2750 20 µM or DMSO 0.1% for 4 hours and samples were sequenced. Previously published RNA-seq data from MCL primary cases, cell lines and normal B-cells were obtained from BLUEPRINT consortium [6,7,9].

GSEA

Microarray data and variance-stabilized expression matrices were analyzed with GSEA v4.0.3. C2 curated gene sets related to stem cells and cell death were used in GEP data. Gene sets derived from differential expression analysis in RNA-seq data (Z138-Ro vs Z138-DMSO) were generated for GSEA (Table S10).

For the analyses, data was randomized by 1000 permutations phenotype (datasets with >7 samples per phenotype) or gene_set (datasets with <7 samples per phenotype). For microarray data, probes were collapsed to Gene Symbol using Max-probe. The metric for ranking genes was Diff_of_Classes, which uses the difference of class means to calculate fold change for log scale data. Enrichment was considered at P-value <0.05 and FDR <0.2.

Plasmid generation

Guide RNA (gRNA) for SOX11 knockout (KO) using CRISPR-Cas9 technology was designed using E-CRISP [10]. Plasmids pLV-Neo-EF1A empty or expressing hMSI2(NM_138962.4)-FLAG were customized by VectorBuilder.

Generation of stable cell lines by lentiviral transduction

HEK-293T were transfected at 60-70% of confluence with lentiviral packaging and envelope plasmids psPAX2 and pCMV-VSV-G (Addgene #12260 and #8454, respectively), and the corresponding plasmid of interest (see Plasmid generation) by using Lipotransfectin (Niborlab). Viral supernatants were collected 48 h after transfection, filtrated and concentrated adding Lenti-X Concentrator (Takara) at 1/10 following manufacturer's instructions. $1.5x10^6$ Granta-519 and Z138 cells were transduced with 300 µl of concentrated lentivirus or 30 µl of commercial Control shRNA Lentiviral Particles (sc-108080; Santa Cruz Biotechnology) by centrifugation for 90 minutes at 32 ºC and 2500 rpm. After 48 h, transduced Z138 and Granta-519 MSI2-KD, MSI2CT or SOX11+ (SOX11 overexpression) cells were selected with 0.2 and 0.4 µg/ml of puromycin (Gibco), respectively, for 1 week. Z138sh4 and sh5MSI2 empty or MSI2-FLAG cells were selected with 500 µg/ml of G-418 (Sigma) for 1 week. For SOX11KO, Z138 GFP+ cells were sorted 72 h after transduction in FACSAria II Cell Sorter (BD) and seeded at 500 cells/well in order to obtain pools with SOX11KO. Disruption of SOX11 locus using CRISPR/Cas9 technology was verified by using the Genomic Cleavage Detection Kit (GeneArt) following manufacturer's indications and by PCR using specific primers (Table S4) followed by Sanger Sequencing. For the generation of Z138shCT-Luc and Z138sh5MSI2-Luc cell lines, Z138shCT and Z138sh5MSI2 GFP+ cells were sorted in FACSAria II Cell 72 h after transduction with GFP-firefly luciferase (pLV430G-oFL-T2A-eGFP vector) lentivirus.

RNA immunoprecipitation

For RNA immunoprecipitation (RIP), Magna RIP RNA-binding protein immunoprecipitation kit (Millipore) was used following manufacturer's instructions. MCL

growing cells were lysed for 5 minutes at 4 ºC and stored at -80 ºC for 2 hours. Magnetic beads protein A/G were washed and incubated with 3 µg of MSI2 or normal Rabbit IgG antibody (Table S5) on rotating wheel for 1 hour at 4 ºC. Then, lysates were incubated with each immune-complex ($30x10^6$ cells per 3 ug of antibody bound to beads) on rotating wheel overnight at 4 ºC, separating a 10% of input fraction before antibody-beads addition. Supernatants (unbound fractions) were saved for western blot (WB) experiment. Immuno-complexes bound to lysates were washed 6 times. One sample incubated with MSI2 antibody was used for WB analysis adding 25 ul of Sample Buffer mixed with DTT and incubating at 95 ºC for 10 minutes. Remaining samples incubated with MSI2 and IgG antibodies and input fraction were used for protein digestion with Proteinase K Buffer at 55 °C for 30 min with shaking. Then, purification of RNA was done by Phenol:Chloroform:Isoamyl Alcohol (125:24:1) method by using Phase Lock Gel Heavy tubes (Quantabio). Ethanol precipitation was performed at -80 ºC overnight. After 80% ethanol wash, RNA was eluted with 12 µl of RNase-free water. NanoDrop (Thermofisher) was used for RNA quantification. RNA from input fraction (1 µg) and the whole RNA eluted from MSI2 and IgG RIP (< 1 µg) were used for cDNA generation by using Verso cDNA Synthesis Kit (Thermofisher) following manufacturer's guidelines and using a blend of oligo dT primers and random hexamers. CDK6, NOTCH1 and GUSB mRNA enrichment was analyzed by qPCR in StepOnePlus (Thermofisher) using Fast SYBR Green Master Mix (Applied Byosistems) and specific primers (Table S4). To quantify relative abundance of each mRNA, Ct of qPCR curves normalized with adjusted input Ct (ΔCt [normalized RIP] = (Ct [RIP] - (Ct [Input] + log2 (% of input/100)) were calculated with the following formula: mRNA enrichment (%) = $100*2^{\Delta Ct\ [normalized\ RIP]}$.

Colony assay

For colony assay, 500 growing cells were mixed with 1 ml of Human Methylcellulose Complete Media (R&D System). and plated in a 24-well plate for triplicate. We counted the number of colonies (1 colony >50 cells) after 14 days and we obtained bright field images (88 images stitched) of the colonies after 21 days, using Cytation 5 Imaging Reader.

Surface and intracellular antigens staining for flow cytometry

Cell death analyses were performed using $5x10^4$ growing MCL cells treated with Ro 08-2750 (Ro) at 1 µM or 5 µM during 24, 48 or 72 h in triplicate. For drug resistance assay, $5x10^4$ Z138 and Granta-519 shMSI2 cells were treated with 0.05 µM and 3 µM of Doxorubicin (Selleckchem), respectively, for 24 hours in triplicate. Cells were incubated with 100 µl of Annexin Binding Buffer mixed with 1.5 µl of Propidium Iodide (PI) and/or 0.4 µl of Annexin V-FITC (eBioscience) for 15 minutes to analyze apoptosis by flow cytometry (FC).

For intracellular staining of cleaved caspase 3, $2x10^5$ growing MCL cells were fixed with paraformaldehyde 4% for 15 minutes. Cells were washed with PBS, permeabilized with Triton 0.5% and FBS 5%, and stained with 10 µl of active caspase 3 antibody conjugated with FITC (Table S5) for 30 minutes at 4 ºC. Cells were washed with PBS and analyzed by FC. For extracellular staining of Fas protein, $2x10^5$ growing MCL cells were washed with PBS and FBS 5%, and stained with 2 µl of Fas or isotype antibodies conjugated with FITC for 20 minutes at 4 ºC. Cells were washed with PBS and analyzed by FC.

For cell cycle analysis, 0.5-1x10^6 growing cells were incubated 1 hour with 20 µM of EdU. Cells were fixed with ethanol 70% and stored at -20 ºC overnight. After 10 minutes of permeabilitzation and blocking with 0.1% of Tween and 1% of BSA, cells were incubated with Click-it reaction buffer for 30 minutes. Then, cells were incubated with PI staining solution (2.5 µg PI and 100 µg RNase A) for 30 minutes and analyzed by FC.

Cytotoxicity assay

MCL and lymphoblastic cell lines were plated into 96-well plate at 5x10^4/well density and treated with increasing concentrations of Ro (1:2-1:10 serial dilutions up to 100 µM, in quadruplicated) for 24 h. Cells were incubated with 10 µl of 12 mM MTT (Invitrogen) during 2-3 h. Formazan crystals were solubilized with Isopropanol:HCl 1M (24:1)

Western Blot

For protein extraction, 3-5x10^6 growing cells were washed with PBS and lysed in RIPA Buffer (Sigma-Aldrich) with Protease and Phosphatase inhibitor cocktail (Thermo Fisher Scientific) for 20 minutes at 4 ºC. Lysates were obtained by centrifugation at 13000 rpm for 15 min, collecting the supernatant. Protein was quantified by Protein Assay (Bio Rad) in Sinergy HT spectrophotometer (λ_{595} nm) and 50-70 µg of total protein mixed with Sample Buffer 5X and DTT was used for WB. Protein extracts were separated by 6–9% SDS–PAGE, transferring them to a 0.45 µm nitrocellulose membrane. Membranes were blocked with 5% of milk powder, BSA or 2.5% of PhosphoBLOCKER (Cell Biolabs) in TBS-T for 1 h and incubated overnight at 4 ºC with specific antibodies (Table S5). After incubation, membranes were washed 3 times with TBS-T and incubated 1 h with the

corresponding secondary antibody (Table S5). After washing, the membrane was incubated with Pierce ECL reagent (Thermo Fisher Scientific) to detect the proteins in ImageQuant LAS4000 (Fujifilm).

ALDEFLUOR assay

For ALDEFLUOR assay (STEMCELL Technologies), $5x10^5$ MCL primary cells were treated with 1 and/or 5 µM of Ro or DMSO 0.05%, for 24 h. The ALDEFLUOR reagent was incubated for 40 minutes at 37 ºC. DEAB was used as negative control. The ALDH activity was defined by the corrected mean fluorescence intensity (MFI ALDEFLUOR – MFI ALDEFLUOR+DEAB samples).

Engraftment in xenograft mice models

For the generation of MSICTLuc+ and MSI2KDLuc+ xenograft mice models, NSG mice (NOD.Cg-$Prkdc^{scid}Il2rg^{tm1Wjl}$/SzJ, Janvier LABS), 20-week of age from both sexes, were intravenously inoculated into their tail veins with $10x10^6$ cells of Z138shCT-Luc and Z138sh5MSI2-Luc cells (5 mice per group). Mice were intraperitoneal injected with 112 mg/kg of D-Luciferin (tebu-bio) and anesthetized with isofluorane (Esteve). Tumor dissemination and growth was captured by luciferase bioimage (LBI) twice a week for 5 weeks. Bioluminiscence was captured 10 min after the injection of D-luciferin by IVIS Lumina III In Vivo Imaging System (PerkinElmer) and total flux (photons/s) from the tumoral cells was quantified using Living Image Software.

MSICT-Luc+ and MSI2KD-Luc+ MCL xenograft mice models were euthanized at 35 days post inoculation. Animals were euthanized according to institutional guidelines. PB

was collected with capillary action blood collection tubes (SAI Infusion Technologies). Spleens were obtained, photographed, weighed, homogenized and filtered through 70 μm nylon cell strainers (Fisher Scientific). Femur and tibias were extracted, cleaned of all connective tissues, and flushed with PBS and EDTA using a 27-gauge needle (Becton Dickinson). Bones were grinded with a mortar and filtered along with the BM flushes by using a 70 μm nylon cell strainer. Erythrocytes from PB, spleens and BM were lysed incubating samples with ACK buffer (Quality Biological) for 10 minutes. Samples were washed with PBS and the process was repeated until pellets remained white. Cells were filtered and resuspended in PBS to determine the percentage of MCL cells measuring GFP+ cells fluorescence by flow cytometry.

The protocol was approved by the animal testing ethical committee of the University of Barcelona.

Statistics

Welch's correction was applied to compare samples with different variances. Overall survival (OS) calculated from time of sampling was used for Kaplan-Meier curves, and log-rank test was performed to measure the association of OS with categorical variables. Maximally selected rank statistics was applied to obtain cutoffs of continuous variables for Kaplan Meier curves (maxstat R package 0.7-25). Ro EC50 was obtained fitting the dose-response curve with non-linear regression methods (log(agonist) vs. response - Variable slope). Pearson correlation was used to measure linear correlation between continuous variables. To calculate sample size enough to ensure adequate power to detect a pre-specified effect, we used G*Power software

(https://stats.oarc.ucla.edu/other/gpower/). For in vivo experiment, we randomized mice to have the same number of animals of each sex in each group.

SUPPLEMENTARY TABLES

Table S1. Clinicomolecular characteristics of the 54 MCL patients used for gene expression profile and survival analysis.

Variable	cMCL (n = 30)	nnMCL (n= 24)
**Clinical data (at diagnosis)**		
Age yr, median (range)	73 (56-99)	64 (45-82)
Male (%)	23/30 (77)	12/24 (50)
**Molecular data**		
SOX11+ (%)	30/30 (100)	0/24 (0)
MSI2 High	25/30 (83)	11/24 (46)
Pretreatment sample (%)	26/30 (87)	21/24 (88)
TP53 alterations (%)	10/25 (40)	7/21 (33)
CDKN2A alterations (%)	6/27 (22)	0/21 (0)
CNA		
CNA High (%)	16/30 (53)	3/24 (12)
CNA Low (%)	11/30 (37)	18/24 (75)
**Follow-up data (n = 40)**		
2-yr OS, % (95% CI)	38 (21-70)	88 (74-100)
n dead, n censored	14, 9	5, 12

CI: confidence interval, CNA: copy number alteration (High >6 CNA), MSI2 High > 8.46 units of mRNA expression.

Table S2. MCL sample information and experimental conditions for RNA extraction, library amplification and RNA-seq.

Dataset	Case or samples	SOX11 status	Files	Method for RNA extraction	Library	Sequencing
MCL primary cases	M027	Negative	1182-01-04TR_1_1_mergedF.fastq.gz	TRIZOL	TruSeq Stranded Total RNA kit with Ribo-Zero Gold	75 bp paired-end
			1182-01-04TR_1_2_mergedR.fastq.gz			
	M029	Positive	1227-02-05TR_1_1_mergedF.fastq.gz			
			1227-02-05TR_1_2_mergedR.fastq.gz			
	M032	Positive	1296-01-04TR_1_1_mergedF.fastq.gz			
			1296-01-04TR_1_2_mergedR.fastq.gz			
	M248	Positive	1359-01-04TR_1_1_mergedF.fastq.gz			
			1359-01-04TR_1_2_mergedR.fastq.gz			
	M399	Positive	1623-04-01BR_1_1_mergedF.fastq.gz			
			1623-04-01BR_1_2_mergedR.fastq.gz			
	M397	Positive	1664-03-02BR_1_1_mergedF.fastq.gz			
			1664-03-02BR_1_2_mergedR.fastq.gz			
	M432	Positive	1807-01-02TR_1_1_mergedF.fastq.gz			
			1807-01-02TR_1_2_mergedR.fastq.gz			
	M435	Positive	1888-01-02BR_1_1_mergedF.fastq.gz			
			1888-01-02BR_1_2_mergedR.fastq.gz			
	M395	Positive	2565-03-01BR_1_1_mergedF.fastq.gz			
			2565-03-01BR_1_2_mergedR.fastq.gz			
	M244	Negative	394-02-05TR_1_1_mergedF.fastq.gz			
			394-02-05TR_1_2_mergedR.fastq.gz			

	M003	Negative	792-04-03TR_1_1_mergedF.fastq.gz			
			792-04-03TR_1_2_mergedR.fastq.gz			
	M076	Negative	817-02-03TR_1_1_mergedF.fastq.gz			
			817-02-03TR_1_2_mergedR.fastq.gz			
Z138 SOX11 knock out	Z138 SOX11-KO 1	Negative	Zcr1_02159AAC_CGATGT.fastq.gz	RNeasy Plus kit	TruSeq RNA Sample Prep Kit v2	50 bp single-end
	Z138 SOX11-KO 2	Negative	Zcr2_02161AAC_CAGATC.fastq.gz			
	Z138 SOX11-KO 3	Negative	Zcr3_02163AAC_AGTTCC.fastq.gz			
	Z138 CT 1	Positive	Zwt1_02158AAC_ATCACG.fastq.gz			
	Z138 CT 2	Positive	Zwt2_02160AAC_TTAGGC.fastq.gz			
	Z138 CT 3	Positive	Zwt3_02162AAC_GATCAG.fastq.gz			
JVM2 overexpressing SOX11	JVM2CT 1	Negative	JC1_25289_ACAGTG.fastq.gz	RNeasy Plus kit	TruSeq RNA Sample Prep Kit v2	50 bp single-end
	JVM2CT 2	Negative	JC2_25290_GCCAAT.fastq.gz			
	JVM2CT 3	Negative	JC3_25291_CTTGTA.fastq.gz			
	JVM2-SOX11+ 1	Positive	JS1_25292_AGTTCC.fastq.gz			
	JVM2-SOX11+ 2	Positive	JS2_25293_GTGAAA.fastq.gz			
	JVM2-SOX11+ 3	Positive	JS3_25294_GAGTGG.fastq.gz			
Z138 MSI2 silencing	Z138sh4MSI2 1	.	Sh4_1_33141_TGACCA_read1_mergedF.fastq.gz	RNeasy Plus kit	TruSeq RNA Sample Prep Kit v2	50 bp paired-end
			Sh4_1_33141_TGACCA_read2_mergedR.fastq.gz			
	Z138sh4MSI2 2	.	Sh4_2_33142_CAGATC_read1_mergedF.fastq.gz			
			Sh4_2_33142_CAGATC_read2_mergedR.fastq.gz			
	Z138sh5MSI2 1	.	Sh5_1_33143_CCGTCC_read1_mergedF.fastq.gz			
			Sh5_1_33143_CCGTCC_read2_mergedR.fastq.gz			
	Z138sh5MSI2 2	.	Sh5_2_33144_GTTTCG_read1_mergedF.fastq.gz			
			Sh5_2_33144_GTTTCG_read2_mergedR.fastq.gz			
	Z138shCT 1.1	.	Zct1_1_33137_CTTGTA_read1_mergedF.fastq.gz			
			Zct1_1_33137_CTTGTA_read2_mergedR.fastq.gz			
	Z138shCT 1.2	.	Zct1_2_33138_GTGAAA_read1_mergedF.fastq.gz			

			Zct1_2_33138_GTGAAA_read2_mergedR.fastq.gz			
	Z138shCT 2.1	.	Zct2_1_33135_ACAGTG_read1_mergedF.fastq.gz			
			Zct2_1_33135_ACAGTG_read2_mergedR.fastq.gz			
	Z138shCT 2.2	.	Zct2_2_33136_GCCAAT_read1_mergedF.fastq.gz			
			Zct2_2_33136_GCCAAT_read2_mergedR.fastq.gz			
Z138 MSI2 inhibition with Ro 08-2750	Z138 DMSO 1	.	DMSO1_R1.fastq.gz	RNeasy Plus kit	TruSeq RNA Sample Prep Kit v2	50 bp paired-end
			DMSO1_R2.fastq.gz			
	Z138 DMSO 2	.	DMSO2_R1.fastq.gz			
			DMSO2_R2.fastq.gz			
	Z138 DMSO 3	.	DMSO3_R1.fastq.gz			
			DMSO3_R2.fastq.gz			
	Z138 RO 1	.	RO1_R1.fastq.gz			
			RO1_R2.fastq.gz			
	Z138 RO 2	.	RO2_R1.fastq.gz			
			RO2_R2.fastq.gz			
	Z138 RO 3	.	RO3_R1.fastq.gz			
			RO3_R2.fastq.gz			

Table S3. Sequences for short hairpin RNAs, MSI2 silencing and guide RNA for SOX11-KO.

RNA	Name	Commercial number	Sequence
gRNA	SOX11.1	-	GTTCCCCGACTACTGCACGC
shRNA	sh4MSI2	TRCN0000062811	CCCAACTTCGTGGCGACCTAT
shRNA	sh5MSI2	TRCN0000417428	TGCAATGCTGATGTTTGATAA

Table S4. Primers used for Sanger Sequencing, to generate luciferase reporter and to perform RT-qPCR.

Gene or region	Primers or Taqman probes
MSI2 luciferase region	Forward: 5'-TCCTTGATCAAAATTTGCATTG-3'
	Reverse: 5'-TATGCGAAAGTAAATGAAGCTGA-3'
SOX11 Sanger sequencing region	Forward: 5'-TGATGTTCGACCTGAGCTTG-3'
	Reverse: 5'-AGATGTCGTGACGCAAAGAAA-3'
CDK6	Forward: 5'-TCTCCCGGCACTTCTGAAAT-3'
	Reverse: 5'-ACACCAGGTAGAAGGACTGC-3'
NOTCH1	Forward: 5'-ACTGTACCGAGGATGTGGAC-3'
	Reverse: 5'-ACACTCGCAGTAGAAGGAGG-3'
MSI2	Forward: 5’-CCAAAGTTGCATTTCCTCGT-3’
	Reverse: 5’-ACAAAGCCAAACCCTCTGTG-3’
SOX11	Forward: 5’-GACCCAGACTGGTGCAAGAC-3’
	Reverse: 5’-GCTGTCCTTCAGCATTTTCC-3’
GUSB	Forward: 5’-CGTGGTTGGAGAGCTCATTT-3’
	Reverse: 5’-GAACGCTGCACTTTTTGGTT-3’
CASP8	hs01018151_m1
CASP10	hs01017899_m1

Table S5. Antibodies used for WB, RIP and FC.

Protein detected	Company	Reference	Dilution WB	WB	RIP	FC
SOX11	Cell Marque	MRQ-58	1/1000	X		
MSI2	Abcam	ab76148	1/1000	X	X	
CDK6	Santa Cruz	sc-56282	1/200	X		
NOTCH1 (D1E11)	Cell Signaling	#3608	1/1000	X		
p-27 (KIP1)	BD Biosciences	554069	1/1000	X		
p-21 (WAF1)	Sigma-Aldrich	P1484	1/1000	X		
p-53	Cell signaling	9282	1/1000	X		
Phospho-p-53 (S15)	Cell signaling	9286	1/1000	X		
Alpha-Tubulin	Sigma-Aldrich	CP06	1/5000	X		
Lamin B1	Abcam	ab65986	1/5000	X		
Anti-rabbit IgG (HRP conjugated)	DAKO	P0217	1/5000	X		
Anti-mouse IgG (HRP conjugated)	DAKO	P0260	1/5000	X		
Anti-rabbit IgG (HRP conjugated)	Cell signaling	#7074	1/1000	X		
Anti-mouse IgG (HRP conjugated)	Cell signaling	#7076	1/1000	X		
Normal rabbit IgG	Millipore	sc-2027			X	
Active Caspase-3 FITC	BD Biosciences	550480				X
Fas FITC (CD95)	Thermo scientific	11-0959-42				X
Mouse IgG1 kappa isotype FITC	Thermo scientific	11-4714-42				X

WB: Western Blot, RIP: RNA immunoprecipitation, FC: Flow cytometry

Table S6. Differentially expressed genes (DEG) between SOX11+ and SOX11- leukemic MCL (GSE79196) with adjusted P-value <0.05 and absolute log_2 fold change >0.7.

Probe	Gene Symbol	logFC	adj.P.Val
204914_s_at	SOX11	5.711	2.78E-22
209524_at	HDGFRP3	4.883	5.88E-17
201310_s_at	NREP	4.569	1.26E-15
228988_at	ZNF711	4.240	1.28E-07
201876_at	PON2	3.742	2.04E-12
215017_s_at	FNBP1L	3.393	1.76E-11
39318_at	TCL1A	3.297	9.29E-06
217504_at	ABCA6	3.283	2.64E-07
202552_s_at	CRIM1	3.269	6.55E-11
212985_at	APBB2	3.165	4.56E-07
201005_at	CD9	3.160	5.05E-07
201540_at	FHL1	3.123	6.55E-11
222258_s_at	SH3BP4	3.075	5.31E-06
239246_at	FARP1	3.046	7.12E-14
204591_at	CHL1	3.011	7.78E-06
202806_at	DBN1	3.003	5.96E-13
222101_s_at	DCHS1	2.924	1.68E-11
203408_s_at	SATB1	2.900	1.44E-08
238983_at	NSUN7	2.897	2.51E-07
203216_s_at	MYO6	2.815	9.27E-08
203695_s_at	DFNA5	2.771	1.35E-07
203240_at	FCGBP	2.733	5.38E-11
213005_s_at	KANK1	2.671	1.91E-07
207705_s_at	NINL	2.662	3.47E-12
225330_at	IGF1R	2.659	2.09E-07
215001_s_at	GLUL	2.645	2.51E-07
213436_at	CNR1	2.636	6.03E-06
1569040_s_at	ANKRD36BP2	2.601	3.28E-03
200644_at	MARCKSL1	2.590	3.23E-11
209674_at	CRY1	2.588	5.49E-04
230441_at	PLEKHG4B	2.547	3.91E-14
212233_at	MAP1B	2.505	4.06E-05
229147_at	RASSF6	2.502	4.20E-03
224215_s_at	DLL1	2.494	2.87E-04
202283_at	SERPINF1	2.462	1.10E-05

202565_s_at	SVIL	2.450	2.26E-08
226184_at	FMNL2	2.392	7.63E-07
214039_s_at	LAPTM4B	2.386	2.11E-05
212190_at	SERPINE2	2.386	5.08E-07
224428_s_at	CDCA7	2.373	2.80E-04
206864_s_at	HRK	2.366	3.97E-04
213478_at	KAZN	2.362	7.21E-08
91816_f_at	MEX3D	2.338	9.65E-07
204208_at	RNGTT	2.336	9.61E-07
225673_at	MYADM	2.334	1.86E-05
200783_s_at	STMN1	2.287	2.95E-11
203355_s_at	PSD3	2.284	2.04E-05
228108_at	PPM1L	2.274	5.88E-06
230875_s_at	ATP11A	2.257	1.21E-06
205234_at	SLC16A4	2.239	6.30E-04
228297_at	---	2.229	6.24E-06
209598_at	PNMA2	2.225	2.96E-04
219255_x_at	IL17RB	2.222	1.22E-05
219895_at	TMEM255A	2.191	2.01E-04
226029_at	VANGL2	2.156	7.74E-10
227126_at	PTPRG	2.140	2.05E-04
214023_x_at	TUBB2B	2.136	4.58E-04
204891_s_at	LCK	2.122	1.08E-06
213194_at	ROBO1	2.095	2.13E-02
217963_s_at	NGFRAP1	2.083	1.32E-04
210835_s_at	CTBP2	2.082	4.31E-06
213060_s_at	CHI3L2	2.060	5.68E-05
226189_at	ITGB8	2.051	2.82E-06
212503_s_at	DIP2C	2.049	1.36E-05
225745_at	LRP6	2.041	1.20E-05
226884_at	LRRN1	2.038	4.47E-06
227013_at	LATS2	2.032	3.75E-05
209570_s_at	NSG1	2.016	5.84E-06
229053_at	SYT17	1.988	8.95E-05
235763_at	SLC44A5	1.983	1.82E-02
222482_at	SSBP3	1.979	1.97E-06
242136_x_at	MGC70870	1.973	4.67E-05
202085_at	TJP2	1.948	2.07E-10
219892_at	TM6SF1	1.946	1.97E-06
242079_at	RGS12	1.925	2.66E-04
204026_s_at	ZWINT	1.922	1.54E-03

209138_x_at	IGLC1 /// IGLJ3	1.922	4.70E-02
203680_at	PRKAR2B	1.921	5.84E-06
223340_at	ATL1	1.920	1.13E-08
222640_at	DNMT3A	1.910	6.74E-09
217979_at	TSPAN13	1.905	6.48E-03
212560_at	SORL1	1.896	4.65E-04
214770_at	MSR1	1.892	2.29E-05
224367_at	BEX2	1.876	1.09E-02
225864_at	FAM84B	1.848	1.90E-03
203386_at	TBC1D4	1.841	2.46E-03
239229_at	PHEX	1.835	1.28E-02
209094_at	DDAH1	1.825	2.49E-05
228476_at	CCDC191	1.823	1.83E-05
208651_x_at	CD24	1.822	4.63E-05
222819_at	CTPS2	1.821	9.81E-07
200762_at	DPYSL2	1.816	5.88E-03
223374_s_at	B3GALNT1	1.804	2.51E-07
231472_at	FBXO15	1.790	4.56E-05
222668_at	KCTD15	1.787	3.03E-05
225532_at	CABLES1	1.766	1.21E-04
204011_at	SPRY2	1.744	1.95E-03
203874_s_at	SMARCA1	1.740	1.28E-04
232664_at	LINC00954	1.740	6.74E-09
212442_s_at	CERS6	1.725	2.30E-03
202946_s_at	BTBD3	1.722	8.17E-04
235109_at	ZBED3	1.715	6.74E-09
239186_at	SERPINB9P1	1.712	2.51E-07
202615_at	GNAQ	1.708	5.09E-03
217975_at	WBP5	1.706	7.99E-04
213135_at	TIAM1	1.697	8.48E-06
225601_at	HMGB3	1.693	3.19E-07
225478_at	MFHAS1	1.687	6.35E-04
209732_at	CLEC2B	1.684	4.70E-02
202988_s_at	RGS1	1.683	4.51E-02
227290_at	CDYL2	1.679	7.86E-06
238021_s_at	CRNDE	1.676	1.48E-02
212372_at	MYH10	1.670	2.04E-04
201307_at	40787	1.669	8.06E-05
225627_s_at	CACHD1	1.662	1.54E-05
229070_at	ADTRP	1.660	2.75E-02
204063_s_at	ULK2	1.654	9.43E-09

235500_at	---	1.649	4.03E-12
229963_at	BEX5	1.648	1.21E-03
236918_s_at	LRRC34	1.641	6.60E-04
223228_at	LDOC1L	1.639	2.19E-10
216080_s_at	FADS3	1.639	2.04E-05
244521_at	TSHZ2	1.631	4.89E-04
201387_s_at	UCHL1	1.629	3.07E-03
204798_at	MYB	1.620	1.75E-04
201417_at	SOX4	1.620	2.78E-02
235048_at	FAM169A	1.617	7.06E-04
200923_at	LGALS3BP	1.616	7.83E-05
218532_s_at	FAM134B	1.613	8.60E-05
203404_at	ARMCX2	1.613	2.73E-04
230664_at	H2BFXP /// LOC100101478	1.611	5.53E-08
218641_at	C11orf95	1.607	2.32E-06
228667_at	AGPAT4	1.607	4.68E-07
203886_s_at	FBLN2	1.605	1.91E-10
201015_s_at	JUP	1.604	2.75E-06
222154_s_at	SPATS2L	1.595	1.32E-04
203688_at	PKD2	1.591	2.92E-05
219132_at	PELI2	1.588	8.94E-05
218412_s_at	GTF2IRD1	1.586	1.81E-08
221054_s_at	TCL6	1.583	2.04E-04
201565_s_at	ID2	1.579	1.75E-02
201825_s_at	SCCPDH	1.576	4.14E-05
239752_at	CECR2	1.568	2.76E-04
213122_at	TSPYL5	1.566	1.66E-02
236126_at	ACVR2B	1.560	1.59E-12
226164_x_at	RIMKLB	1.557	9.69E-04
230495_at	LINC01102	1.556	1.76E-02
206314_at	ZKSCAN7	1.552	8.40E-06
202404_s_at	COL1A2	1.545	4.43E-03
39729_at	PRDX2	1.534	7.94E-03
222696_at	AXIN2	1.526	2.32E-03
224733_at	CMTM3	1.523	2.40E-06
225147_at	CYTH3	1.522	1.15E-03
223627_at	MEX3B	1.522	7.06E-08
228737_at	TOX2	1.520	2.25E-02
230593_at	GRIK3	1.519	2.37E-03
226846_at	PHYHD1	1.515	3.81E-05
213906_at	MYBL1	1.514	6.72E-03

204992_s_at	PFN2	1.512	5.02E-06
206554_x_at	SETMAR	1.511	5.96E-08
201710_at	MYBL2	1.507	9.15E-05
227361_at	HS3ST3B1	1.505	2.27E-04
205803_s_at	TRPC1	1.505	4.85E-03
218856_at	TNFRSF21	1.490	5.88E-03
236798_at	LINC00888	1.485	7.06E-04
202967_at	GSTA4	1.482	2.65E-05
232239_at	LINC00865	1.481	1.04E-08
212812_at	SERINC5	1.476	2.78E-04
226157_at	TFDP2	1.474	4.47E-06
212320_at	TUBB	1.461	4.28E-05
203795_s_at	BCL7A	1.458	1.70E-03
209790_s_at	CASP6	1.450	5.34E-07
201677_at	HMCES	1.450	1.50E-04
205780_at	BIK	1.448	1.97E-03
213358_at	MTCL1	1.440	1.42E-03
235230_at	PLCXD2	1.440	1.39E-03
206414_s_at	ASAP2	1.429	1.45E-04
202016_at	MEST	1.428	8.05E-04
212012_at	PXDN	1.427	4.90E-04
229376_at	PROX1	1.425	3.26E-04
238870_at	KCNK9	1.421	8.30E-05
211026_s_at	MGLL	1.419	1.71E-04
225286_at	ARSD	1.418	5.31E-06
201938_at	CDK2AP1	1.412	1.58E-05
210690_at	KLRC4	1.406	2.20E-03
206167_s_at	ARHGAP6	1.403	5.73E-04
218066_at	SLC12A7	1.402	9.32E-07
228167_at	KLHL6	1.402	3.47E-03
206404_at	FGF9	1.397	2.84E-04
225224_at	NOL4L	1.397	3.01E-04
215111_s_at	TSC22D1	1.396	2.22E-04
218309_at	CAMK2N1	1.391	9.96E-05
213484_at	ADD2	1.391	3.21E-03
229114_at	GAB1	1.388	3.02E-02
218285_s_at	BDH2	1.386	2.06E-10
225688_s_at	PHLDB2	1.383	4.65E-04
206142_at	ZNF135	1.369	1.68E-07
203748_x_at	RBMS1	1.367	3.08E-10
227860_at	CPXM1	1.364	1.22E-05

226430_at	RELL1	1.361	2.65E-02
219855_at	NUDT11	1.358	6.16E-04
244370_at	KIAA2022	1.355	1.02E-02
223253_at	EPDR1	1.351	9.82E-06
204805_s_at	H1FX	1.350	7.74E-05
203029_s_at	PTPRN2	1.348	9.07E-03
226000_at	CTTNBP2NL	1.346	3.41E-04
222431_at	SPIN1	1.346	3.53E-12
230446_at	LOC101927811	1.339	1.27E-06
224759_s_at	TMEM263	1.338	3.56E-05
224952_at	TANC2	1.336	2.01E-04
229002_at	FAM69B	1.335	1.62E-04
200824_at	GSTP1	1.333	9.81E-07
225665_at	ZAK	1.331	2.48E-02
213792_s_at	INSR	1.329	2.19E-03
228560_at	CHDH	1.318	9.15E-05
218820_at	C14orf132	1.317	1.99E-04
214617_at	PRF1	1.314	1.80E-03
221526_x_at	PARD3	1.311	4.38E-06
216356_x_at	BAIAP3	1.308	9.15E-05
239185_at	ABCA9	1.308	8.20E-04
224772_at	NAV1	1.305	6.74E-09
232478_at	MIR181A2HG	1.304	1.86E-05
225655_at	UHRF1	1.304	3.07E-02
226003_at	KIF21A	1.296	7.84E-04
201830_s_at	NET1	1.289	1.15E-02
58780_s_at	ARHGEF40	1.287	3.39E-05
212820_at	DMXL2	1.284	1.43E-02
232303_at	ZNF608	1.284	7.71E-04
211798_x_at	IGLJ3	1.284	2.70E-02
213348_at	CDKN1C	1.284	3.45E-03
209892_at	FUT4	1.284	1.42E-04
205593_s_at	PDE9A	1.282	3.56E-05
219569_s_at	SLC35G2	1.278	1.89E-04
226905_at	FAM101B	1.273	1.61E-02
201976_s_at	MYO10	1.273	2.60E-03
218773_s_at	MSRB2	1.264	8.03E-04
230561_s_at	KANSL1L	1.264	1.79E-04
223361_at	ABRACL	1.260	1.58E-04
208147_s_at	CYP2C8	1.256	2.15E-03
217894_at	KCTD3	1.254	1.75E-04

212658_at	LHFPL2	1.253	6.26E-03
200832_s_at	SCD	1.252	9.38E-03
203080_s_at	BAZ2B	1.246	4.41E-04
238480_at	TTC39C	1.243	2.78E-03
202729_s_at	LTBP1	1.240	1.75E-05
203987_at	FZD6	1.238	7.34E-03
228280_at	ZC3HAV1L	1.238	3.73E-04
236656_s_at	LOC100288911	1.229	2.73E-06
209695_at	PTP4A3	1.228	6.12E-05
204647_at	HOMER3	1.228	8.47E-05
226065_at	PRICKLE1	1.221	1.08E-06
200897_s_at	PALLD	1.221	1.43E-02
201887_at	IL13RA1	1.221	2.40E-02
226364_at	HIP1	1.220	3.65E-04
225105_at	C12orf75	1.220	6.74E-03
215440_s_at	BEX4	1.218	1.92E-02
225485_at	CEP41	1.208	9.13E-05
204103_at	CCL4	1.206	6.50E-03
221606_s_at	HMGN5	1.202	1.29E-05
202478_at	TRIB2	1.200	2.73E-02
228620_at	LOC101927027	1.199	2.46E-04
226869_at	MEGF6	1.198	4.11E-03
226112_at	SGCB	1.195	6.21E-03
203562_at	FEZ1	1.195	4.89E-02
221556_at	CDC14B	1.193	2.05E-03
235142_at	ZBTB8A	1.190	1.90E-03
227985_at	LOC100506098	1.190	2.02E-02
242912_at	POTEM	1.188	1.19E-02
240413_at	PYHIN1	1.188	3.66E-02
225603_s_at	TRIQK	1.188	3.15E-03
205414_s_at	ARHGAP44	1.186	3.63E-06
204702_s_at	NFE2L3	1.186	4.02E-05
215807_s_at	PLXNB1	1.182	4.06E-10
212761_at	TCF7L2	1.176	6.71E-03
201525_at	APOD	1.175	4.30E-02
226188_at	LGALSL	1.173	1.44E-05
212099_at	RHOB	1.173	2.65E-02
202068_s_at	LDLR	1.171	6.05E-03
212737_at	GM2A	1.166	2.53E-03
201063_at	RCN1	1.166	7.83E-05
231579_s_at	TIMP2	1.164	2.26E-02

227449_at	EPHA4	1.164	1.79E-02
231996_at	N4BP2	1.163	1.54E-04
203431_s_at	ARHGAP32	1.161	5.37E-03
219179_at	DACT1	1.161	9.69E-06
221538_s_at	PLXNA1	1.157	2.34E-04
38671_at	PLXND1	1.156	2.99E-04
218706_s_at	GRAMD3	1.156	1.34E-04
242584_at	FAM161A	1.155	9.38E-04
201204_s_at	RRBP1	1.150	1.97E-05
225450_at	AMOTL1	1.150	9.19E-06
205692_s_at	CD38	1.144	1.97E-03
212768_s_at	OLFM4	1.144	1.09E-03
229553_at	PGM2L1	1.132	3.03E-05
228654_at	SPIN4	1.129	5.11E-03
217865_at	RNF130	1.128	4.92E-06
218418_s_at	KANK2	1.125	2.35E-03
204137_at	GPR137B	1.121	8.57E-03
212771_at	FAM171A1	1.121	2.16E-03
201578_at	PODXL	1.119	8.06E-05
1568780_at	LOC729732	1.117	4.20E-05
201681_s_at	DLG5	1.116	2.84E-04
219583_s_at	SPATA7	1.114	8.79E-05
235735_at	TNFSF8	1.107	3.90E-02
203072_at	MYO1E	1.104	2.15E-03
209789_at	CORO2B	1.098	1.56E-02
213273_at	TENM4	1.096	3.47E-03
236635_at	ZNF667	1.095	4.94E-02
202786_at	STK39	1.094	1.06E-02
202933_s_at	YES1	1.092	2.99E-03
204749_at	NAP1L3	1.090	1.82E-02
226043_at	GPSM1	1.087	9.96E-07
226726_at	MBOAT2	1.083	9.96E-05
1553956_at	TMEM237	1.082	2.29E-04
223740_at	AGPAT4-IT1	1.080	9.12E-07
226344_at	ZMAT1	1.079	2.78E-02
235146_at	TMCC3	1.079	2.40E-02
224341_x_at	TLR4	1.078	6.06E-04
203130_s_at	KIF5C	1.077	1.15E-02
218974_at	SOBP	1.072	1.48E-02
227336_at	DTX1	1.066	5.03E-04
224802_at	NDFIP2	1.065	2.46E-03

233142_at	LINC01224	1.062	8.01E-04
207426_s_at	TNFSF4	1.062	7.72E-03
224789_at	DCAF12	1.060	3.40E-02
221047_s_at	MARK1	1.059	1.94E-03
228054_at	TMEM44	1.059	2.65E-05
201560_at	CLIC4	1.059	1.56E-02
226590_at	ZNF618	1.059	2.75E-06
208950_s_at	ALDH7A1	1.059	6.60E-04
225293_at	COL27A1	1.059	4.53E-04
52975_at	MVB12B	1.056	1.50E-06
227666_at	DCLK2	1.052	3.36E-06
226408_at	TEAD2	1.051	1.97E-03
238365_s_at	C1orf228	1.049	4.15E-03
235408_x_at	ZNF117	1.048	2.66E-03
204301_at	KBTBD11	1.047	2.72E-03
214829_at	AASS	1.047	9.98E-03
203693_s_at	E2F3	1.042	3.26E-05
208869_s_at	GABARAPL1	1.042	1.70E-02
205489_at	CRYM	1.039	2.24E-03
238002_at	GOLIM4	1.039	1.17E-03
230793_at	LRRC16A	1.039	1.93E-02
225368_at	HIPK2	1.038	8.89E-04
225731_at	ANKRD50	1.035	1.33E-03
201243_s_at	ATP1B1	1.034	4.57E-02
228906_at	TET1	1.033	3.76E-04
219368_at	NAP1L2	1.028	1.14E-02
203585_at	ZNF185	1.025	1.56E-03
204720_s_at	DNAJC6	1.023	1.89E-03
227742_at	CLIC6	1.021	4.01E-02
238029_s_at	SLC16A14	1.020	2.48E-02
231807_at	KIAA1217	1.019	3.56E-02
225061_at	DNAJA4	1.014	6.84E-03
224827_at	UBTD2	1.013	7.21E-04
219213_at	JAM2	1.011	2.67E-04
223327_x_at	GOLGA2P10	0.997	1.69E-06
223044_at	SLC40A1	0.994	2.46E-02
204083_s_at	TPM2	0.991	1.21E-02
228707_at	CLDN23	0.987	5.47E-03
205771_s_at	AKAP7	0.987	4.78E-02
213720_s_at	SMARCA4	0.986	2.01E-04
204730_at	RIMS3	0.985	2.20E-03

234983_at	C12orf49	0.985	1.23E-04
236831_at	CCDC50	0.984	5.75E-03
222196_at	LOC389906	0.982	1.59E-02
202430_s_at	PLSCR1	0.982	2.83E-02
219173_at	MYO15B	0.975	4.19E-04
232282_at	WNK3	0.971	4.61E-04
1570351_at	ADAMTS6	0.971	4.17E-02
226436_at	RASSF4	0.970	7.22E-03
209301_at	CA2	0.969	4.67E-02
226456_at	RMI2	0.966	2.20E-02
206028_s_at	MERTK	0.962	9.33E-04
209014_at	MAGED1	0.960	1.22E-05
204226_at	STAU2	0.959	1.11E-02
223089_at	VEZT	0.958	1.17E-04
226120_at	TTC8	0.953	1.96E-03
212699_at	SCAMP5	0.950	8.80E-05
208782_at	FSTL1	0.950	1.05E-03
1560132_a_at	LOC101927432	0.949	3.89E-02
203283_s_at	HS2ST1	0.947	7.10E-04
201656_at	ITGA6	0.947	5.32E-03
209197_at	SEPT11 /// SYT11	0.946	3.96E-02
211700_s_at	TRO	0.946	2.51E-07
231188_at	ZSCAN2	0.945	2.65E-05
223366_at	ZNF704	0.943	1.62E-02
227542_at	SOCS6	0.942	9.98E-04
210401_at	P2RX1	0.940	4.29E-04
216548_x_at	HMGB3P1	0.935	3.16E-04
209082_s_at	COL18A1	0.933	3.36E-02
212136_at	ATP2B4	0.933	8.15E-03
238603_at	MIR9-3HG	0.932	5.27E-03
222857_s_at	KCNMB4	0.930	5.20E-05
206102_at	GINS1	0.928	4.44E-02
202897_at	SIRPA	0.928	2.15E-02
201448_at	TIA1	0.926	2.34E-02
213479_at	NPTX2	0.925	2.34E-02
219232_s_at	EGLN3	0.925	1.58E-05
221909_at	RNFT2	0.924	6.78E-03
228557_at	L3MBTL4	0.922	2.60E-02
212921_at	SMYD2	0.921	1.62E-04
205888_s_at	JAKMIP2	0.919	1.32E-03
229515_at	PAWR	0.916	1.13E-03

203046_s_at	TIMELESS	0.916	2.89E-03
218847_at	IGF2BP2	0.916	4.82E-03
204173_at	MYL6B	0.916	3.64E-03
209642_at	BUB1	0.915	3.38E-03
219423_x_at	TNFRSF25	0.915	2.02E-02
1558041_a_at	KIAA0895L	0.915	2.50E-06
221081_s_at	DENND2D	0.914	2.98E-02
230489_at	CD5	0.913	4.08E-04
200665_s_at	SPARC	0.911	3.95E-02
226676_at	ZNF521	0.910	2.52E-02
226548_at	SBK1	0.908	2.84E-04
226358_at	APH1B	0.902	8.14E-04
226374_at	CXADR	0.902	5.17E-04
222761_at	BIVM	0.902	1.32E-02
214032_at	ZAP70	0.901	9.24E-03
209267_s_at	SLC39A8	0.898	3.51E-02
205181_at	ZSCAN9	0.896	2.33E-05
225017_at	CCDC14	0.891	7.12E-03
235122_at	HIVEP3	0.891	2.00E-02
232111_at	TUNAR	0.890	2.14E-03
227834_at	TXLNB	0.889	4.41E-02
219815_at	GAL3ST4	0.885	7.71E-06
228973_at	DLG2	0.885	6.70E-03
201425_at	ALDH2	0.884	1.84E-03
209737_at	MAGI2	0.883	3.90E-04
203140_at	BCL6	0.883	1.07E-02
212792_at	DPY19L1	0.879	2.12E-03
244033_at	CEP128	0.878	1.75E-03
207399_at	BFSP2	0.877	4.89E-02
209163_at	CYB561	0.876	1.13E-03
209118_s_at	TUBA1A	0.876	2.68E-05
219787_s_at	ECT2	0.874	5.71E-03
219123_at	ZNF232	0.873	1.73E-02
225732_at	KLHL42	0.871	7.71E-03
235721_at	DTX3	0.871	1.77E-04
230187_s_at	LOC101927268 /// LOC102724851 /// LOC105374994	0.870	3.40E-02
1565951_s_at	CHML	0.868	2.94E-03
226563_at	SMAD2	0.865	5.60E-06
209108_at	TSPAN6	0.862	8.63E-03
243010_at	MSI2	0.857	8.99E-03

34726_at	CACNB3	0.854	1.08E-06
207130_at	ZMYND8	0.852	1.40E-02
223513_at	CENPJ	0.851	1.52E-02
212895_s_at	ABR	0.846	2.47E-03
228397_at	TUG1	0.845	8.89E-04
223381_at	NUF2	0.843	3.51E-02
213435_at	SATB2	0.843	2.74E-02
204170_s_at	CKS2	0.840	3.93E-02
201397_at	PHGDH	0.839	3.26E-05
201548_s_at	KDM5B	0.837	5.47E-03
208898_at	ATP6V1D	0.837	1.97E-02
1558801_at	NNT-AS1	0.836	5.83E-03
214712_at	SNX29P2	0.835	3.14E-02
203725_at	GADD45A	0.833	2.88E-02
220423_at	PLA2G2D	0.833	3.26E-05
1556049_at	RTN4	0.832	1.73E-03
211071_s_at	MLLT11	0.831	4.88E-02
210220_at	FZD2	0.830	1.29E-04
205848_at	GAS2	0.829	3.03E-04
228377_at	KLHL14	0.827	1.05E-03
1569608_x_at	LOC102723382 /// LOC102723891 /// LOC105379516 /// LOC105379552	0.826	1.13E-03
210046_s_at	IDH2	0.826	6.45E-03
200884_at	CKB	0.824	1.41E-03
205327_s_at	ACVR2A	0.823	6.60E-04
204822_at	TTK	0.822	3.87E-02
204454_at	LDOC1	0.820	1.28E-02
220054_at	IL23A	0.820	5.79E-04
225020_at	DAB2IP	0.820	1.85E-02
203305_at	F13A1	0.817	4.01E-02
222184_at	---	0.816	1.23E-04
205109_s_at	ARHGEF4	0.815	1.54E-04
223062_s_at	PSAT1	0.814	3.71E-02
235170_at	ZNF92	0.812	4.71E-03
203016_s_at	SSX2IP	0.811	2.61E-03
218694_at	ARMCX1	0.811	2.20E-02
219446_at	RIC8B	0.811	1.53E-03
204531_s_at	BRCA1	0.811	1.14E-02
219736_at	TRIM36	0.809	1.34E-02
201518_at	CBX1	0.809	1.09E-02

231931_at	PRDM15	0.809	1.32E-02
218676_s_at	PCTP	0.808	3.51E-03
239231_at	ZNF101	0.808	2.64E-03
202315_s_at	BCR	0.806	3.73E-03
217950_at	NOSIP	0.805	3.64E-03
224817_at	SH3PXD2A	0.805	1.74E-02
241503_at	---	0.800	5.37E-03
205088_at	MAMLD1	0.800	4.33E-03
228365_at	CPNE8	0.799	1.97E-02
230100_x_at	PAK1	0.799	1.38E-02
231899_at	ZC3H12C	0.797	1.78E-03
212651_at	RHOBTB1	0.797	1.44E-02
213587_s_at	ATP6V0E2	0.796	3.54E-03
226925_at	PXYLP1	0.795	7.72E-03
226439_s_at	NBEA	0.795	6.45E-03
224819_at	TCEAL8	0.794	1.44E-03
206695_x_at	ZNF43	0.793	4.45E-03
213452_at	ZNF184	0.793	1.64E-03
221002_s_at	TSPAN14	0.791	1.80E-03
225958_at	PHC1	0.791	7.86E-06
209781_s_at	KHDRBS3	0.790	4.78E-02
225021_at	ZNF532	0.790	7.31E-04
219687_at	HHAT	0.789	2.58E-03
219038_at	MORC4	0.789	2.34E-03
214710_s_at	CCNB1	0.788	2.72E-02
211031_s_at	CLIP2	0.788	5.68E-04
212032_s_at	PTOV1	0.787	1.98E-03
229331_at	SPATA18	0.786	4.36E-02
200782_at	ANXA5	0.785	6.85E-03
216860_s_at	GDF11	0.784	2.50E-03
209035_at	MDK	0.784	1.12E-03
201054_at	HNRNPA0	0.782	3.76E-03
201897_s_at	CKS1B	0.780	4.51E-02
204079_at	TPST2	0.775	2.33E-02
224578_at	RCC2	0.775	6.06E-06
208636_at	ACTN1	0.774	4.83E-02
224758_at	C7orf73	0.771	4.02E-03
222547_at	MAP4K4	0.769	3.41E-04
225335_at	ZNF496	0.769	1.61E-04
226249_at	SNX30	0.769	5.36E-03
206862_at	ZNF254	0.767	1.58E-02

216905_s_at	ST14	0.767	4.04E-02
228920_at	ZNF260	0.766	1.22E-02
227108_at	STARD9	0.765	1.48E-02
203878_s_at	MMP11	0.763	2.18E-05
202179_at	BLMH	0.762	2.92E-03
202107_s_at	MCM2	0.762	1.72E-02
240637_at	WDR41	0.761	1.19E-02
205051_s_at	KIT	0.761	8.03E-03
202154_x_at	TUBB3	0.760	1.84E-02
209132_s_at	COMMD4	0.759	7.67E-03
202073_at	OPTN	0.758	1.05E-02
203228_at	PAFAH1B3	0.755	8.17E-03
203817_at	GUCY1B3	0.754	1.46E-02
212765_at	CAMSAP2	0.754	1.21E-02
35147_at	MCF2L	0.754	2.96E-05
221203_s_at	YEATS2	0.752	3.73E-03
212886_at	CCDC69	0.752	2.90E-03
238469_at	OGFRL1	0.751	6.72E-03
229700_at	ZNF738	0.751	9.33E-03
206498_at	OCA2	0.749	4.15E-02
218326_s_at	LGR4	0.749	1.35E-02
220001_at	PADI4	0.748	5.00E-03
236725_at	WWC1	0.748	8.21E-04
203142_s_at	AP3B1	0.747	1.14E-02
229742_at	C15orf61	0.746	3.94E-03
225436_at	ABHD17C	0.745	2.15E-03
219342_at	CASD1	0.743	7.07E-04
209921_at	SLC7A11	0.742	9.23E-03
210609_s_at	TP53I3	0.741	1.35E-03
202789_at	PLCG1	0.738	4.60E-06
231887_s_at	PALD1	0.737	1.38E-02
226420_at	MECOM	0.736	1.01E-02
226571_s_at	PTPRS	0.736	2.72E-02
216755_at	OSBPL10	0.734	4.90E-02
201201_at	CSTB	0.733	1.47E-02
204886_at	PLK4	0.733	4.94E-02
227917_at	LOC100506990	0.731	4.37E-04
230166_at	KIAA1958	0.731	6.45E-03
219304_s_at	PDGFD	0.728	3.84E-02
235953_at	ZNF610	0.728	2.12E-03
201928_at	PKP4	0.727	3.69E-03

208939_at	SEPHS1	0.726	3.47E-05
41660_at	CELSR1	0.726	2.16E-03
219372_at	IFT81	0.725	1.15E-04
207786_at	CYP2R1	0.724	4.81E-03
213627_at	MAGED2	0.723	3.30E-04
226446_at	HES6	0.722	6.50E-03
202594_at	LEPROTL1	0.720	5.11E-03
230578_at	ZNF471	0.719	3.20E-02
210115_at	RPL39L	0.717	2.02E-02
238853_at	RAB3IP	0.717	1.84E-03
225841_at	HENMT1	0.717	3.31E-02
235758_at	PNMA6A	0.717	4.52E-03
209605_at	TST	0.717	6.78E-03
226152_at	TTC7B	0.716	2.68E-05
223063_at	C1orf198	0.716	1.09E-04
212639_x_at	TUBA1A /// TUBA1B	0.714	4.28E-05
213093_at	PRKCA	0.713	3.36E-02
225897_at	MARCKS	0.711	3.56E-02
1555841_at	MSANTD3	0.710	1.15E-04
200765_x_at	CTNNA1	0.710	2.52E-04
242722_at	LMO7	0.709	1.35E-02
222317_at	PDE3B	0.707	1.04E-02
226143_at	RAI1	0.705	2.27E-04
228780_at	POU3F3	0.703	4.45E-02
203724_s_at	RUFY3	0.702	1.94E-02
210024_s_at	UBE2E3	0.702	1.36E-04
221794_at	DOCK6	0.701	2.85E-04
219654_at	HACD1	0.701	5.04E-03
212274_at	LPIN1	0.700	4.45E-03
1559862_at	COPA	-0.703	2.40E-02
1552584_at	IL12RB1	-0.703	9.38E-04
218611_at	IER5	-0.708	1.74E-03
1562368_at	CARD11	-0.708	1.53E-03
211502_s_at	CDK14	-0.712	4.71E-02
226130_at	RPS16	-0.713	2.00E-02
226010_at	SLC25A23	-0.719	4.20E-03
201531_at	ZFP36	-0.720	2.53E-03
201473_at	JUNB	-0.721	1.56E-02
1553626_a_at	EFCAB13	-0.734	2.21E-02
224352_s_at	CFL2	-0.737	5.99E-03
204396_s_at	GRK5	-0.738	2.10E-02

203377_s_at	CDC40	-0.739	2.11E-03
228846_at	MXD1	-0.739	3.89E-02
1559263_s_at	ZC3H12D	-0.741	2.86E-02
244519_at	ASXL1	-0.743	7.69E-03
217848_s_at	PPA1	-0.750	1.40E-02
242069_at	CBX5	-0.757	8.21E-03
243213_at	STAT3	-0.758	1.13E-02
210656_at	EED	-0.759	1.56E-02
228697_at	HINT3	-0.761	4.70E-02
203836_s_at	MAP3K5	-0.765	5.88E-03
224252_s_at	FXYD5	-0.769	4.92E-03
200787_s_at	PEA15	-0.775	2.20E-03
238127_at	GAS6-AS1	-0.776	4.17E-02
238987_at	B4GALT1	-0.777	1.54E-03
1554464_a_at	CRTAP	-0.778	8.10E-03
225530_at	MOB3A	-0.778	1.04E-02
216915_s_at	PTPN12	-0.778	1.90E-02
1559942_at	MDFIC	-0.779	4.03E-02
207681_at	CXCR3	-0.784	2.84E-04
225567_at	INAFM2	-0.796	2.55E-03
235389_at	PHF20	-0.802	2.97E-03
201534_s_at	UBL3	-0.804	1.09E-02
226528_at	MTX3	-0.807	3.81E-02
207160_at	IL12A	-0.809	1.54E-03
219457_s_at	RIN3	-0.810	1.54E-03
225576_at	GINM1	-0.814	1.14E-03
215012_at	ZNF451	-0.817	2.51E-02
204244_s_at	DBF4	-0.818	5.90E-03
223999_at	PPIL2	-0.818	1.79E-02
204082_at	PBX3	-0.819	5.32E-03
232521_at	PCSK7	-0.821	4.48E-05
238694_at	DGKE	-0.821	4.05E-03
218700_s_at	RAB29	-0.822	5.72E-03
239377_at	EIF1AD	-0.823	8.15E-04
1554910_at	PRKD3	-0.827	4.51E-02
243977_at	LOC541472	-0.827	1.35E-02
230380_at	THAP2	-0.827	6.78E-03
223254_s_at	G2E3	-0.829	3.28E-02
205882_x_at	ADD3	-0.833	2.57E-03
1557719_at	PIKFYVE	-0.833	9.10E-03
1554258_a_at	DNAJC5B	-0.836	2.49E-02

235359_at	NRROS	-0.836	7.24E-03
207992_s_at	AMPD3	-0.837	3.61E-04
1556072_at	LINC00528	-0.842	5.12E-04
218346_s_at	SESN1	-0.847	4.26E-03
238025_at	MLKL	-0.848	3.33E-02
202255_s_at	SIPA1L1	-0.848	8.17E-04
206508_at	CD70	-0.849	9.60E-03
202771_at	PIEZO1	-0.864	1.83E-02
222292_at	CD40	-0.869	5.23E-03
205677_s_at	DLEU1	-0.871	2.00E-03
242218_at	PPARD	-0.872	4.45E-02
203542_s_at	KLF9	-0.875	3.17E-02
235850_at	FAM162A	-0.875	2.76E-05
239283_at	TMED5	-0.885	1.01E-04
230536_at	PBX4	-0.892	9.03E-03
1554522_at	CNNM2	-0.892	1.11E-02
223172_s_at	MTFP1	-0.898	7.45E-05
207431_s_at	DEGS1	-0.898	7.45E-05
205878_at	POU6F1	-0.899	4.42E-04
201695_s_at	PNP	-0.900	5.71E-03
204118_at	CD48	-0.905	4.37E-04
238057_at	USP45	-0.913	4.27E-03
201963_at	ACSL1	-0.927	2.54E-02
235020_at	TAF4B	-0.932	1.28E-03
222528_s_at	SLC25A37	-0.933	3.66E-04
214787_at	DENND4A	-0.936	3.85E-03
1569599_at	SAMSN1	-0.944	2.38E-02
204180_s_at	ZBTB43	-0.952	2.02E-02
242857_at	FARP2	-0.952	1.54E-04
224828_at	CPEB4	-0.955	1.15E-02
210214_s_at	BMPR2	-0.962	1.16E-02
243699_at	LOC100507006	-0.981	3.41E-04
220684_at	TBX21	-0.985	2.88E-03
1565544_at	RNF141	-0.991	1.93E-02
202431_s_at	MYC	-0.991	1.90E-02
203397_s_at	GALNT3	-0.993	6.14E-03
47105_at	DUS2	-0.996	1.19E-05
206896_s_at	GNG7	-0.997	4.64E-02
217553_at	STEAP1B	-0.999	5.75E-03
239494_at	FBXW7	-0.999	8.21E-04
226402_at	CYP2U1	-1.006	8.77E-03

229543_at	---	-1.011	4.78E-02
214467_at	GPR65	-1.014	4.49E-04
242020_s_at	ZBP1	-1.017	5.99E-03
200670_at	XBP1	-1.018	1.48E-03
212522_at	PDE8A	-1.030	2.24E-03
207000_s_at	PPP3CC	-1.031	3.74E-04
242676_at	NDUFV2-AS1	-1.037	6.39E-03
223596_at	SLC12A6	-1.047	1.77E-02
215565_at	LOC101929272	-1.058	5.27E-03
235421_at	MAP3K8	-1.060	6.32E-03
219624_at	BAG4	-1.064	4.15E-03
211744_s_at	CD58	-1.065	3.73E-03
210785_s_at	THEMIS2	-1.077	1.87E-02
242260_at	MATR3	-1.090	2.89E-03
222874_s_at	CLN8	-1.092	3.95E-05
218772_x_at	TMEM38B	-1.094	1.62E-04
1557557_at	MATN1-AS1	-1.095	4.61E-02
1554486_a_at	GFOD1	-1.104	1.56E-02
208683_at	CAPN2	-1.105	7.66E-03
221211_s_at	MAP3K7CL	-1.116	3.94E-02
205419_at	GPR183	-1.124	4.80E-03
232213_at	PELI1	-1.127	9.77E-04
241844_x_at	TMEM156	-1.134	1.72E-02
227189_at	CPNE5	-1.137	2.01E-02
208092_s_at	FAM49A	-1.139	1.97E-02
205542_at	STEAP1	-1.153	2.70E-03
242903_at	IFNGR1	-1.159	5.88E-03
239777_at	LINC01588	-1.169	1.91E-07
206983_at	CCR6	-1.178	8.74E-03
206907_at	TNFSF9	-1.181	2.31E-02
208010_s_at	PTPN22	-1.183	2.28E-03
219429_at	FA2H	-1.183	1.61E-03
208438_s_at	FGR	-1.190	1.04E-02
227607_at	STAMBPL1	-1.200	3.09E-03
203508_at	TNFRSF1B	-1.205	1.04E-03
204866_at	JADE3	-1.230	3.32E-02
214169_at	SUN1	-1.232	6.91E-04
209200_at	MEF2C	-1.233	3.83E-02
1564970_at	SETDB2	-1.238	5.88E-04
204780_s_at	FAS	-1.241	1.86E-03
235353_at	SEL1L3	-1.244	2.42E-03

207826_s_at	ID3	-1.259	3.03E-02
238009_at	SOX5	-1.273	1.17E-03
209406_at	BAG2	-1.280	1.50E-04
205801_s_at	RASGRP3	-1.283	2.79E-02
228032_s_at	DENND1B	-1.296	1.00E-04
212828_at	SYNJ2	-1.332	2.47E-06
205987_at	CD1C	-1.388	4.64E-02
206519_x_at	SIGLEC6	-1.392	3.92E-02
226051_at	SELM	-1.409	2.80E-04
222108_at	AMIGO2	-1.429	3.65E-04
205691_at	SYNGR3	-1.498	1.52E-03
225133_at	KLF3	-1.536	1.04E-02
1557267_s_at	GEN1	-1.536	8.63E-03
219049_at	CSGALNACT1	-1.621	2.92E-02
220118_at	ZBTB32	-1.783	4.26E-07
205207_at	IL6	-1.845	6.37E-04
209583_s_at	CD200	-1.894	1.53E-03
236226_at	BTLA	-1.924	3.18E-06
222838_at	SLAMF7	-1.929	2.70E-03
206715_at	TFEC	-2.034	1.62E-02
209470_s_at	GPM6A	-2.042	3.96E-02
206181_at	SLAMF1	-2.386	4.68E-07

FC: Fold Change; adj P Val: adjusted P-value

Table S7. Gene ontology (GO) of functional stem cell-related categories and their respective genes.

GO ID	GO term	Genes
GO:0005020	stem cell factor receptor activity	KIT
GO:0005173	stem cell factor receptor binding	KITLG, SH2B3, SPRED1, SPRED2
GO:0017145	stem cell division	CUL3, ESRRB, GNL3, PAFAH1B1, THOC2, TIAL1, WWTR1
GO:0019827	stem cell population maintenance	ARID1A, BMPR1A, CDC73, CTNNB1, CTR9, DDX6, DIS3L2, EIF4E, EIF4ENIF1, EOMES, ESRRB, FGF4, FZD7, GNL3, KDM4C, KIT, KLF4, LEO1, LIF, LIN28A, LSM1, MED10, MED12, MED14, MED15, MED21, MED24, MED27, MED28, MED30, MED6, MED7, METTL14, METTL3, MTF2, NANOG, NIPBL, NKAP, NODAL, NOTCH2, PADI4, PAF1, PELO, PHF19, PRDM14, PRDM16, RIF1, RTF1, SALL4, SETD6, SMC1A, SMC3, STAT3, TBX3, TET1, TPT1, TRIM8, TUT4, ZNF358
GO:0030718	germ-line stem cell population maintenance	NANOS2, PIWIL2, PRDM14
GO:0035019	somatic stem cell population maintenance	ASCL2, BCL9, BCL9L, BRAF, CDX2, CUL4A, DPPA4, ELF5, EPHA1, FGF10, FGF2, FOXD3, FOXP1, GATA2, HES1, KIT, KLF10, KLF4, LDB1, LDB2, LIG4, LIN28A, LRP5, NANOG, NOG, NR2E1, PBX1, POLR2A, POLR2B, POLR2C, POLR2D, POLR2E, POLR2F, POLR2G, POLR2H, POLR2I, POLR2J, POLR2K, POLR2L, POU5F1, PRDM14, PRDM16, RAF1, RBPJ, REST, SALL1, SALL4, SFRP1, SKI, SMAD2, SMAD4, SOX2, SOX4, SOX9, SPI1, STAT3, TDGF1,

		VANGL2, VPS72, WNT7A, YAP1, ZFP36L2, ZHX2, ZIC3, ZSCAN10
GO:0035701	hematopoietic stem cell migration	BCL11B, GPLD1, KIT
GO:0036334	epidermal stem cell homeostasis	KIF3A
GO:0036335	intestinal stem cell homeostasis	LGR4
GO:0044338	canonical Wnt signaling pathway involved in mesenchymal stem cell differentiation	FZD1, WNT3
GO:0048103	somatic stem cell division	CDKN2A, FZD7, HOXB4, KIT, NOTCH1, TGFB2, VANGL2, WNT3A, WNT7A, ZFP36L2
GO:0048133	male germ-line stem cell asymmetric division	ETV5, ING2, STRA8, ZBTB16
GO:0048863	stem cell differentiation	A2M, DNMT3L, ELL3, EPCAM, EPOP, ESR1, FOXO4, GPM6A, HMGA2, HOXA7, JARID2, KIT, LIF, LIN28A, MSX1, MSX2, MTF2, NELFB, OSR1, PAX2, PDX1, PHF19, PHF5A, PSMD11, PUM1, RUNX2, SETD2, SETD6, SHC4, SOX10, SOX17, SOX21, ZNF281
GO:0048864	stem cell development	MSI2, PTPRC, SETD2, SHH, WNT7A
GO:0048866	stem cell fate specification	SOX17, SOX18
GO:0060218	hematopoietic stem cell differentiation	ACE, BATF, CDK6, CHD2, ERCC2, HOXB4, LMBR1L, MEOX1, SFRP1, SP7, SRF, TAL1, TP53, XRCC5
GO:0060529	squamous basal epithelial stem cell differentiation involved in prostate gland acinus development	FGFR2, TP63
GO:0061484	hematopoietic stem cell homeostasis	ADAR, CCN3, NLE1, TCIRG1, UBAP2L
GO:0071425	hematopoietic stem cell proliferation	ARIH2, CD34, CTC1, ETV6, MECOM, NKAP, RUNX1, SART3, SFRP2, WNT1, WNT10B, WNT2B, WNT5A, YTHDF2
GO:0072038	mesenchymal stem cell maintenance involved in nephron morphogenesis	SIX2, WNT9B
GO:0072089	stem cell proliferation	ABCB1, CD34, FGF2, NES, RNF43, TRIM71, WNT3, WNT7B, ZNRF3

GO:0072091	regulation of stem cell proliferation	AGO3, HMGB2, NF2, SOX17, SOX18, YAP1, ZFP36L1
GO:0097150	neuronal stem cell population maintenance	ASPM, CDH2, DLL1, FANCC, FANCD2, FOXO1, FOXO3, FUT10, HES1, HES5, HOOK3, IGF2BP1, JAG1, MCPH1, MMP24, NOTCH1, PCM1, PROX1, PRRX1, REST, SOX2, SRRT, SS18
GO:0097168	mesenchymal stem cell proliferation	SCRG1, SIX2
GO:0097241	hematopoietic stem cell migration to bone marrow	GAS6, GPLD1, JAM2, JAM3
GO:1902033	regulation of hematopoietic stem cell proliferation	ACE, EIF2AK2, PIM1
GO:1902034	negative regulation of hematopoietic stem cell proliferation	MIR221, MIR222
GO:1902035	positive regulation of hematopoietic stem cell proliferation	ATXN1L, KITLG, N4BP2L2, PDCD2, THPO
GO:1902036	regulation of hematopoietic stem cell differentiation	ABL1, CBFB, CDK6, EIF2AK2, GATA1, GATA2, GATA3, ITCH, KMT2A, LDB1, LMO1, LMO2 , METTL3, MYB, OSM, PSMA1, PSMA2, PSMA3, PSMA4, PSMA5, PSMA6, PSMA7, PSMA8, PSMB1, PSMB10, PSMB11, PSMB2, PSMB3, PSMB4, PSMB5, PSMB6, PSMB7, PSMB8, PSMB9, PSMC1, PSMC2, PSMC3, PSMC4, PSMC5, PSMC6, PSMD1, PSMD10, PSMD11, PSMD12, PSMD13, PSMD14, PSMD2, PSMD3, PSMD4, PSMD5, PSMD6, PSMD7, PSMD8, PSMD9, PSME1, PSME2, PSME3, PSME4, PSMF1, PUS7, RUNX1, SEM1, SETD1A, TAL1, TCF12, TCF3, TP73, YAP1, YTHDF2
GO:1902037	negative regulation of hematopoietic stem cell	HSPA9, N4BP2L2, NFE2L2

	differentiation	
GO:1902038	positive regulation of hematopoietic stem cell differentiation	FOXC1
GO:1902455	negative regulation of stem cell population maintenance	LOXL2, WNT9B, ZNF706
GO:1902459	positive regulation of stem cell population maintenance	ESRRB, KDM2B, NCOA3, PRDM14, REST, YAP1, ZNF322
GO:1902461	negative regulation of mesenchymal stem cell proliferation	MIR29B1, MIR29B2
GO:1902462	positive regulation of mesenchymal stem cell proliferation	CITED1, LTBP3, VEGFC
GO:1904672	regulation of somatic stem cell population maintenance	MYC, TAF5L, TAF6L
GO:1904673	negative regulation of somatic stem cell population maintenance	MIR145
GO:1904674	positive regulation of somatic stem cell population maintenance	LBH, TP63
GO:1904676	negative regulation of somatic stem cell division	MIR145
GO:1904677	positive regulation of somatic stem cell division	LBH
GO:1905322	positive regulation of mesenchymal stem cell migration	ACKR3, CXCR4, FBXO5
GO:1905474	canonical Wnt signaling pathway involved in stem cell proliferation	WNT3
GO:2000035	regulation of stem cell division	CDK2AP2, ESRRB, EVI2B, NAP1L2, NCOA3, PRDM15, SFRP2, SOX17
GO:2000036	regulation of stem cell population maintenance	CNOT1, CNOT2, CNOT3, ELAVL1, HMGA2, KAT2A, KDM3A, NODAL, PTN, SAV1, SMO, TAL1, WDR43, ZC3H13
GO:2000103	positive regulation of mammary stem cell proliferation	LBH
GO:2000473	positive regulation of hematopoietic stem cell migration	CCR2, PTPRC
GO:2000647	negative regulation of stem cell proliferation	FBLN1, FERMT1, KDF1, OVOL1, OVOL2, SNAI2
GO:2000648	positive regulation of stem cell proliferation	EPCAM, GJA1, HMGA2, HNRNPU, KDM1A, NANOG, NR2E1, PTPRC, SIRT6, SOX11, TBX3, TERT

GO:2000736	regulation of stem cell differentiation	GDNF, KDM3A, KDM4C, MIR146A, MIR147B, NSUN2, OCIAD1, TEAD2
GO:2000737	negative regulation of stem cell differentiation	CDK12, CDK13, ESRRB, H18, HES1, HES5, HNRNPU, JAG1, LBH, NELFB, NOTCH1, STAT3, TRIM6, YAP1, ZFP36L2
GO:2000738	positive regulation of stem cell differentiation	HOXB4, NUDT21, PTN, PWP1, RBM24, SP7, TACSTD2
GO:2000739	regulation of mesenchymal stem cell differentiation	PDGFRA
GO:2000740	negative regulation of mesenchymal stem cell differentiation	REST
GO:2000741	positive regulation of mesenchymal stem cell differentiation	LTBP3
GO:2000798	negative regulation of amniotic stem cell differentiation	REST

GO: gene ontology

Table S8. FIMO analysis on 4 SOX11+ MCL specific ATAC-seq peaks matching with Sox family consensus binding motifs.

Motif id	Motif alt id	Sequence name	Start	Stop	Strand	Score	P-value	Q-value	Matched sequence
PB0167.1	Sox13_2	ATAC_peak_2	301	317	-	12.34	8.86E-06	0.069	TTAGTGGGTGGGTGACT
MA0514.1	Sox3	ATAC_peak_2	107	116	+	14.07	1.11E-05	0.105	CCTTTGTCTG
MA1563.1	SOX18	ATAC_peak_2	62	69	-	10.77	1.47E-05	0.067	CACAATGC
MA1563.1	SOX18	ATAC_peak_4	197	204	+	10.77	1.47E-05	0.067	CACAATGC
PB0168.1	Sox14_2	ATAC_peak_2	104	118	-	12.55	1.72E-05	0.165	GTCAGACAAAGGTCC
PB0166.1	Sox12_2	ATAC_peak_2	104	119	-	12.05	1.88E-05	0.164	AGTCAGACAAAGGTCC
PB0167.1	Sox13_2	ATAC_peak_3	1215	1231	+	11.56	2.06E-05	0.069	GTGCAGGGAGGGAAGAG
PB0167.1	Sox13_2	ATAC_peak_3	202	218	-	11.22	2.95E-05	0.069	GTTGTGGGTGGGGTGAG
PB0167.1	Sox13_2	ATAC_peak_1	164	180	+	11.17	3.10E-05	0.069	ATCCCGGGTGGGAGCTG
MA1120.1	SOX13	ATAC_peak_4	1623	1633	-	12.35	3.42E-05	0.265	GTACAATGGGG
PB0166.1	Sox12_2	ATAC_peak_4	1185	1200	+	11.40	3.45E-05	0.164	AGAGAGACAAAGACAG
PB0168.1	Sox14_2	ATAC_peak_2	59	73	-	11.82	3.57E-05	0.171	CATACACAATGCTGG
PB0167.1	Sox13_2	ATAC_peak_4	2373	2389	-	11.01	3.62E-05	0.069	TTACTGGGAGGAAAATG
MA0143.1	Sox2	ATAC_peak_4	1662	1676	-	11.69	3.82E-05	0.366	CCATTGTCATAAAGA
MA0143.2	Sox2	ATAC_peak_4	1662	1676	-	11.63	3.98E-05	0.381	CCATTGTCATAAAGA
MA0143.4	SOX2	ATAC_peak_4	1668	1678	+	12.35	4.09E-05	0.205	TGACAATGGTT
PB0171.1	Sox18_2	ATAC_peak_1	471	486	-	9.94	4.19E-05	0.403	GCACCAAATGCATGAA
MA0143.4	SOX2	ATAC_peak_4	1623	1633	-	12.31	4.26E-05	0.205	GTACAATGGGG
MA0078.1	Sox17	ATAC_peak_4	1669	1677	-	11.94	4.55E-05	0.436	ACCATTGTC
MA0077.1	SOX9	ATAC_peak_4	1625	1633	+	11.22	4.77E-05	0.461	CCATTGTAC
MA0515.1	Sox6	ATAC_peak_2	107	116	+	12.53	4.96E-05	0.273	CCTTTGTCTG
MA0143.3	Sox2	ATAC_peak_2	107	114	+	13.38	5.04E-05	0.313	CCTTTGTC
MA1120.1	SOX13	ATAC_peak_4	1668	1678	+	11.75	5.50E-05	0.265	TGACAATGGTT

MA0515.1	Sox6	ATAC_peak_4	1188	1197	-	12.23	5.73E-05	0.273	TCTTTGTCTC
MA0514.1	Sox3	ATAC_peak_4	1188	1197	-	11.98	5.81E-05	0.275	TCTTTGTCTC
MA0442.2	SOX10	ATAC_peak_4	1188	1198	+	12.01	6.25E-05	0.601	GAGACAAAGAC
MA0143.3	Sox2	ATAC_peak_4	1669	1676	-	13.10	6.51E-05	0.313	CCATTGTC
PB0167.1	Sox13_2	ATAC_peak_4	2020	2036	-	10.40	6.55E-05	0.104	TGGGTGGGTGGGGGATG
MA1563.1	SOX18	ATAC_peak_4	606	613	-	10.05	6.81E-05	0.208	GACAACGC
PB0176.1	Sox5_2	ATAC_peak_1	14	28	-	9.09	8.00E-05	0.770	AGGCATAAATAAGGG
MA1152.1	SOX15	ATAC_peak_4	1667	1676	-	10.82	8.90E-05	0.856	CCATTGTCAT

ATAC_peak_1: chr17:57,440,795-57,441,325
ATAC_peak_2: chr17:57,463,187-57,463,603
ATAC_peak_3: chr17:57,499,225-57,500,470
ATAC_peak_4: chr17:57,525,240-57,527,912

Table S9. Differentially expressed genes upon MSI2 knockdown in MCL cell lines with adjusted P-value <0.1 and absolute log_2-transformed fold change >0.65.

Gene Stable ID	Gene Symbol	logFC	adj.P.Val
ENSG00000162591.16	MEGF6	5.733	4.31E-06
ENSG00000188404.10	SELL	5.539	4.66E-11
ENSG00000165929.13	TC2N	4.958	1.59E-90
ENSG00000255819.7	KLRC4-KLRK1	4.936	5.88E-10
ENSG00000124813.23	RUNX2	4.821	2.09E-16
ENSG00000182870.13	GALNT9	4.786	3.65E-07
ENSG00000113657.13	DPYSL3	4.612	1.24E-16
ENSG00000276776.4	TC2N	4.094	1.13E-46
ENSG00000163564.15	PYHIN1	3.978	4.22E-23
ENSG00000153283.13	CD96	3.865	1.16E-05
ENSG00000165695.10	AK8	3.724	5.28E-39
ENSG00000182871.16	COL18A1	3.469	9.23E-19
ENSG00000013619.14	MAMLD1	3.354	1.90E-09
ENSG00000135046.14	ANXA1	3.240	1.31E-13
ENSG00000188064.10	WNT7B	3.136	4.92E-10
ENSG00000140030.6	GPR65	3.072	1.15E-15
ENSG00000151136.15	BTBD11	2.998	2.03E-06
ENSG00000080854.15	IGSF9B	2.881	1.39E-03
ENSG00000185924.7	RTN4RL1	2.838	5.23E-11
ENSG00000137968.16	SLC44A5	2.824	2.04E-07
ENSG00000221890.5	NPTXR	2.752	1.69E-11
ENSG00000106605.11	BLVRA	2.714	1.21E-04
ENSG00000026508.19	CD44	2.710	2.19E-04
ENSG00000188263.10	IL17REL	2.691	1.59E-22
ENSG00000175445.17	LPL	2.599	5.70E-03
ENSG00000100427.16	MLC1	2.576	9.53E-03
ENSG00000115183.15	TANC1	2.570	4.57E-55
ENSG00000071205.12	ARHGAP10	2.536	7.23E-05
ENSG00000105976.15	MET	2.523	2.03E-07
ENSG00000214889.3	RPS9P1	2.439	9.34E-06
ENSG00000137332.19	DDR1	2.410	7.20E-04
ENSG00000274174.4	NLRP7	2.337	4.28E-29
ENSG00000188536.13	HBA2	2.334	4.27E-04
ENSG00000144369.13	FAM171B	2.308	1.72E-02
ENSG00000153233.13	PTPRR	2.298	7.31E-23

ENSG00000161249.21	DMKN	2.208	9.06E-03
ENSG00000100218.12	RSPH14	2.150	3.47E-05
ENSG00000124429.18	POF1B	2.059	2.16E-09
ENSG00000168079.17	SCARA5	2.045	1.01E-02
ENSG00000169744.13	LDB2	2.041	1.21E-02
ENSG00000169752.17	NRG4	2.033	1.76E-02
ENSG00000278195.2	SSTR3	2.027	1.85E-24
ENSG00000135631.17	RAB11FIP5	2.024	1.86E-02
ENSG00000159231.6	CBR3	2.014	1.38E-19
ENSG00000104728.16	ARHGEF10	1.993	1.15E-03
ENSG00000125910.6	S1PR4	1.988	1.35E-17
ENSG00000147408.14	CSGALNACT1	1.978	1.57E-06
ENSG00000169064.12	ZBBX	1.932	2.35E-03
ENSG00000106976.21	DNM1	1.897	4.26E-03
ENSG00000133101.10	CCNA1	1.884	4.72E-05
ENSG00000227507.3	LTB	1.838	1.70E-08
ENSG00000139626.16	ITGB7	1.824	3.54E-21
ENSG00000255274.9	SMIM35	1.816	4.41E-03
ENSG00000162188.6	GNG3	1.815	2.27E-09
ENSG00000169442.9	CD52	1.797	1.86E-25
ENSG00000274726.4	ARHGEF10	1.773	8.73E-03
ENSG00000213809.9	KLRK1	1.763	1.07E-05
ENSG00000158815.11	FGF17	1.748	2.25E-03
ENSG00000141655.17	TNFRSF11A	1.742	1.57E-15
ENSG00000141068.14	KSR1	1.735	7.26E-19
ENSG00000177694.16	NAALADL2	1.718	2.55E-06
ENSG00000009790.15	TRAF3IP3	1.716	3.16E-30
ENSG00000197747.9	S100A10	1.708	3.43E-03
ENSG00000088992.18	TESC	1.704	2.14E-07
ENSG00000065717.15	TLE2	1.703	8.22E-07
ENSG00000189410.12	SH2D5	1.694	4.18E-04
ENSG00000010278.14	CD9	1.678	7.43E-42
ENSG00000099282.10	TSPAN15	1.674	1.42E-05
ENSG00000176463.14	SLCO3A1	1.643	1.27E-07
ENSG00000152128.13	TMEM163	1.641	1.35E-02
ENSG00000237330.3	RNF223	1.639	6.15E-04
ENSG00000158856.18	DMTN	1.633	3.68E-17
ENSG00000118257.16	NRP2	1.629	2.01E-05
ENSG00000135919.13	SERPINE2	1.629	1.13E-05
ENSG00000060566.14	CREB3L3	1.623	5.93E-03

ENSG00000204487.8	LTB	1.574	1.60E-02
ENSG00000099204.20	ABLIM1	1.573	1.48E-06
ENSG00000108001.14	EBF3	1.570	2.81E-03
ENSG00000182901.16	RGS7	1.569	5.56E-05
ENSG00000064787.13	BCAS1	1.567	4.00E-03
ENSG00000176092.15	CRYBG2	1.544	2.86E-04
ENSG00000179104.9	TMTC2	1.529	5.32E-06
ENSG00000008283.16	CYB561	1.519	9.23E-18
ENSG00000103187.8	COTL1	1.514	2.14E-53
ENSG00000120278.16	PLEKHG1	1.511	7.65E-03
ENSG00000275896.5	PRSS2	1.503	9.54E-05
ENSG00000187634.12	SAMD11	1.490	3.03E-04
ENSG00000228913.2	UBD	1.484	4.23E-04
ENSG00000140092.14	FBLN5	1.469	4.72E-05
ENSG00000132359.15	RAP1GAP2	1.467	7.08E-03
ENSG00000167281.19	RBFOX3	1.452	2.30E-05
ENSG00000131015.5	ULBP2	1.448	1.55E-05
ENSG00000075275.17	CELSR1	1.434	7.16E-07
ENSG00000123388.4	HOXC11	1.419	1.14E-03
ENSG00000107719.9	PALD1	1.410	1.63E-03
ENSG00000234424.2		1.404	2.19E-02
ENSG00000204580.13	DDR1	1.385	3.74E-02
ENSG00000091831.23	ESR1	1.379	7.04E-11
ENSG00000008517.17	IL32	1.376	4.17E-09
ENSG00000166289.6	PLEKHF1	1.376	2.12E-03
ENSG00000078081.8	LAMP3	1.358	4.72E-03
ENSG00000169896.18	ITGAM	1.357	2.65E-02
ENSG00000133328.4	PLAAT2	1.355	4.12E-03
ENSG00000175084.12	DES	1.347	1.72E-06
ENSG00000131019.11	ULBP3	1.344	2.51E-08
ENSG00000186854.11	TRABD2A	1.343	2.05E-03
ENSG00000131981.16	LGALS3	1.339	6.67E-07
ENSG00000134901.13	POGLUT2	1.332	2.21E-05
ENSG00000130755.13	GMFG	1.329	8.45E-13
ENSG00000148468.17	FAM171A1	1.320	2.41E-06
ENSG00000254838.5	GVINP1	1.298	1.07E-03
ENSG00000198003.12	CCDC151	1.297	1.39E-04
ENSG00000137767.14	SQOR	1.295	4.23E-07
ENSG00000183018.9	SPNS2	1.281	2.22E-06
ENSG00000196781.16	TLE1	1.281	1.89E-17

ENSG00000180644.8	PRF1	1.277	1.06E-10
ENSG00000075426.12	FOSL2	1.276	6.86E-03
ENSG00000126217.21	MCF2L	1.261	3.80E-05
ENSG00000001617.12	SEMA3F	1.251	3.35E-02
ENSG00000135318.12	NT5E	1.247	2.46E-05
ENSG00000085552.17	IGSF9	1.245	3.30E-02
ENSG00000135709.12	KIAA0513	1.245	1.30E-05
ENSG00000154102.11	C16orf74	1.241	7.21E-09
ENSG00000168785.8	TSPAN5	1.235	2.08E-03
ENSG00000132026.14	RTBDN	1.227	7.16E-04
ENSG00000115107.20	STEAP3	1.221	1.80E-12
ENSG00000188859.7	FAM78B	1.207	1.58E-06
ENSG00000075618.18	FSCN1	1.205	2.29E-14
ENSG00000167723.15	TRPV3	1.201	3.37E-03
ENSG00000164483.17	SAMD3	1.195	6.87E-03
ENSG00000163412.13	EIF4E3	1.187	1.66E-06
ENSG00000226321.5	CROCC2	1.181	1.24E-02
ENSG00000175707.9	KDF1	1.180	2.71E-02
ENSG00000101695.9	RNF125	1.172	6.47E-08
ENSG00000113303.12	BTNL8	1.169	1.21E-02
ENSG00000188015.10	S100A3	1.162	2.33E-03
ENSG00000198286.9	CARD11	1.142	8.88E-09
ENSG00000073146.16	MOV10L1	1.129	5.13E-04
ENSG00000146054.18	TRIM7	1.123	7.17E-09
ENSG00000121743.4	GJA3	1.121	9.84E-09
ENSG00000168685.15	IL7R	1.118	4.43E-02
ENSG00000125046.15	SSUH2	1.113	1.64E-08
ENSG00000182809.11	CRIP2	1.110	1.12E-02
ENSG00000099812.9	MISP	1.107	4.81E-04
ENSG00000026103.22	FAS	1.098	8.73E-09
ENSG00000164543.7	STK17A	1.095	1.57E-19
ENSG00000089327.15	FXYD5	1.091	8.79E-10
ENSG00000136960.12	ENPP2	1.086	1.85E-24
ENSG00000082512.15	TRAF5	1.074	1.94E-03
ENSG00000175793.12	SFN	1.071	2.21E-02
ENSG00000174307.7	PHLDA3	1.068	4.00E-09
ENSG00000267385.1		1.065	2.59E-02
ENSG00000000938.13	FGR	1.064	2.19E-16
ENSG00000167641.11	PPP1R14A	1.062	4.60E-03
ENSG00000136848.17	DAB2IP	1.058	4.50E-09

ENSG00000214456.8	PLIN5	1.050	8.65E-04
ENSG00000128536.16	CDHR3	1.048	2.16E-02
ENSG00000135638.14	EMX1	1.047	1.42E-05
ENSG00000124942.14	AHNAK	1.039	2.75E-04
ENSG00000082293.13	COL19A1	1.038	5.03E-10
ENSG00000159733.14	ZFYVE28	1.036	1.76E-02
ENSG00000275565.4	ALOX5	1.036	8.30E-04
ENSG00000168487.19	BMP1	1.033	2.57E-03
ENSG00000100767.17	PAPLN	1.026	6.79E-04
ENSG00000099864.18	PALM	1.016	2.59E-06
ENSG00000107679.14	PLEKHA1	1.015	4.24E-09
ENSG00000103811.17	CTSH	1.012	2.35E-11
ENSG00000168243.11	GNG4	1.012	6.67E-07
ENSG00000151150.22	ANK3	1.011	5.33E-05
ENSG00000167676.4	PLIN4	0.998	1.94E-02
ENSG00000042493.16	CAPG	0.994	8.39E-08
ENSG00000074964.17	ARHGEF10L	0.992	5.82E-12
ENSG00000166211.8	SPIC	0.989	4.27E-02
ENSG00000119888.11	EPCAM	0.983	1.16E-03
ENSG00000160213.8	CSTB	0.978	3.22E-16
ENSG00000168874.13	ATOH8	0.977	1.36E-03
ENSG00000156194.18	PPEF2	0.975	1.87E-02
ENSG00000132386.11	SERPINF1	0.971	3.63E-19
ENSG00000068078.18	FGFR3	0.966	4.92E-04
ENSG00000103647.13	CORO2B	0.964	3.57E-09
ENSG00000171130.18	ATP6V0E2	0.964	6.72E-04
ENSG00000111913.20	RIPOR2	0.963	1.25E-09
ENSG00000176383.9	B3GNT4	0.955	1.78E-02
ENSG00000147642.17	SYBU	0.947	4.27E-02
ENSG00000170379.20	TCAF2	0.946	2.49E-03
ENSG00000112280.16	COL9A1	0.945	1.25E-05
ENSG00000166394.15	CYB5R2	0.944	2.97E-02
ENSG00000163683.12	SMIM14	0.935	1.89E-09
ENSG00000184368.16	MAP7D2	0.934	2.51E-02
ENSG00000196684.12	HSH2D	0.931	3.53E-26
ENSG00000165698.16	SPACA9	0.920	6.79E-04
ENSG00000187688.15	TRPV2	0.909	7.17E-05
ENSG00000198873.12	GRK5	0.909	6.50E-05
ENSG00000143367.16	TUFT1	0.908	3.74E-07
ENSG00000081059.20	TCF7	0.906	7.58E-12

ENSG00000073910.22	FRY	0.905	1.51E-02
ENSG00000203523.3	TAS2R2P	0.892	1.47E-02
ENSG00000179855.9	GIPC3	0.892	2.60E-03
ENSG00000197520.10	FAM177B	0.889	3.56E-04
ENSG00000178773.15	CPNE7	0.883	1.03E-03
ENSG00000107796.13	ACTA2	0.883	9.45E-04
ENSG00000128815.19	WDFY4	0.880	1.21E-05
ENSG00000103021.9	CCDC113	0.879	5.00E-04
ENSG00000162543.6	UBXN10	0.870	1.91E-03
ENSG00000169213.7	RAB3B	0.870	9.08E-09
ENSG00000175048.17	ZDHHC14	0.869	7.08E-08
ENSG00000128284.19	APOL3	0.868	1.29E-11
ENSG00000132623.16	ANKEF1	0.866	8.46E-06
ENSG00000155792.10	DEPTOR	0.866	3.16E-04
ENSG00000274137.2	MYOM2	0.862	6.55E-08
ENSG00000158715.6	SLC45A3	0.858	1.56E-02
ENSG00000187902.11	SHISA7	0.852	9.26E-03
ENSG00000165140.11	FBP1	0.843	4.20E-02
ENSG00000166501.14	PRKCB	0.842	1.36E-02
ENSG00000127863.15	TNFRSF19	0.841	3.29E-11
ENSG00000196782.12	MAML3	0.840	1.24E-02
ENSG00000168961.17	LGALS9	0.838	3.07E-13
ENSG00000198626.17	RYR2	0.837	1.19E-02
ENSG00000101445.10	PPP1R16B	0.836	4.98E-04
ENSG00000168404.13	MLKL	0.833	5.58E-07
ENSG00000168993.15	CPLX1	0.831	2.22E-06
ENSG00000100298.15	APOBEC3H	0.825	1.59E-02
ENSG00000164078.13	MST1R	0.811	8.61E-03
ENSG00000133321.11	PLAAT4	0.810	2.22E-06
ENSG00000012124.17	CD22	0.809	4.08E-10
ENSG00000262473.5	GART	0.808	6.03E-03
ENSG00000117013.17	KCNQ4	0.801	2.06E-02
ENSG00000187486.5	KCNJ11	0.796	4.34E-05
ENSG00000175984.15	DENND2C	0.791	2.79E-02
ENSG00000134061.5	CD180	0.791	1.33E-03
ENSG00000188820.13	CALHM6	0.784	3.90E-04
ENSG00000196924.18	FLNA	0.774	3.51E-12
ENSG00000117148.8	ACTL8	0.763	1.29E-03
ENSG00000114450.10	GNB4	0.762	5.08E-05
ENSG00000150625.16	GPM6A	0.762	3.29E-02

ENSG00000134470.21	IL15RA	0.758	7.48E-04
ENSG00000188042.8	ARL4C	0.755	8.18E-07
ENSG00000113916.18	BCL6	0.755	2.72E-02
ENSG00000144749.13	LRIG1	0.751	5.95E-04
ENSG00000101384.12	JAG1	0.744	1.19E-07
ENSG00000131370.16	SH3BP5	0.739	2.72E-05
ENSG00000128394.17	APOBEC3F	0.734	1.32E-04
ENSG00000105289.15	TJP3	0.725	8.70E-03
ENSG00000163331.12	DAPL1	0.725	4.57E-05
ENSG00000273045.6	C2orf15	0.724	5.28E-05
ENSG00000083454.22	P2RX5	0.721	8.30E-07
ENSG00000008441.16	NFIX	0.718	1.69E-04
ENSG00000181625.17	SLX1B	0.717	5.17E-03
ENSG00000175920.18	DOK7	0.714	1.26E-03
ENSG00000165238.16	WNK2	0.707	3.32E-03
ENSG00000073861.3	TBX21	0.702	2.25E-02
ENSG00000013364.19	MVP	0.699	3.86E-05
ENSG00000125170.11	DOK4	0.699	8.27E-03
ENSG00000285277.1		0.698	2.07E-02
ENSG00000176381.6	PRR18	0.695	1.33E-02
ENSG00000185345.23	PRKN	0.694	4.33E-02
ENSG00000036448.10	MYOM2	0.694	4.74E-05
ENSG00000172986.13	GXYLT2	0.694	8.51E-03
ENSG00000138028.16	CGREF1	0.694	2.28E-06
ENSG00000167646.14	DNAAF3	0.690	4.55E-02
ENSG00000217801.10		0.688	1.06E-04
ENSG00000284009.2	PLSCR3	0.685	6.21E-10
ENSG00000187838.17	PLSCR3	0.685	6.21E-10
ENSG00000204219.11	TCEA3	0.683	5.56E-07
ENSG00000197540.8	GZMM	0.682	3.40E-02
ENSG00000239713.9	APOBEC3G	0.681	5.11E-10
ENSG00000005844.18	ITGAL	0.678	1.66E-02
ENSG00000160789.20	LMNA	0.677	3.79E-03
ENSG00000119703.15	ZC2HC1C	0.676	1.10E-02
ENSG00000285369.2		0.671	1.05E-10
ENSG00000162627.17	SNX7	0.667	2.42E-03
ENSG00000107551.21	RASSF4	0.666	5.70E-06
ENSG00000243811.10	APOBEC3D	0.663	1.31E-04
ENSG00000163393.13	SLC22A15	0.662	1.92E-03
ENSG00000138867.16	GUCD1	0.660	2.48E-16

ENSG00000136573.14	BLK	0.652	3.82E-10
ENSG00000132744.8	ACY3	0.652	5.80E-07
ENSG00000114126.17	TFDP2	-0.655146	8.21E-26
ENSG00000022567.9	SLC45A4	-0.658559	4.46E-04
ENSG00000115884.11	SDC1	-0.662774	1.25E-15
ENSG00000231247.8	PPP1R18	-0.664511	2.60E-03
ENSG00000172725.14	CORO1B	-0.669477	2.08E-02
ENSG00000140948.13	ZCCHC14	-0.674858	2.09E-02
ENSG00000186352.9	ANKRD37	-0.675078	8.83E-05
ENSG00000148400.12	NOTCH1	-0.683076	4.06E-11
ENSG00000138386.17	NAB1	-0.688688	1.53E-06
ENSG00000171843.16	MLLT3	-0.690466	4.35E-02
ENSG00000120693.13	SMAD9	-0.691561	1.13E-15
ENSG00000138758.11	SEPTIN11	-0.694946	1.32E-22
ENSG00000162068.2	NTN3	-0.701919	2.51E-04
ENSG00000105928.16	GSDME	-0.70473	4.42E-04
ENSG00000138760.10	SCARB2	-0.71029	2.61E-08
ENSG00000111885.7	MAN1A1	-0.717625	3.80E-19
ENSG00000123104.12	ITPR2	-0.720572	2.31E-07
ENSG00000223459.6	TCAF1P1	-0.725029	3.05E-02
ENSG00000129484.13	PARP2	-0.727206	1.12E-26
ENSG00000267041.6	ZNF850	-0.727599	3.86E-06
ENSG00000172339.10	ALG14	-0.730806	9.88E-04
ENSG00000117480.16	FAAH	-0.733258	2.68E-02
ENSG00000100368.14	CSF2RB	-0.735245	3.20E-13
ENSG00000171115.4	GIMAP8	-0.738115	8.72E-04
ENSG00000180787.6	ZFP3	-0.739256	3.74E-05
ENSG00000184979.10	USP18	-0.746846	1.05E-08
ENSG00000066294.15	CD84	-0.747231	2.35E-03
ENSG00000197121.15	PGAP1	-0.750548	3.13E-05
ENSG00000183458.14		-0.751503	1.70E-03
ENSG00000122367.19	LDB3	-0.751691	7.68E-07
ENSG00000134107.5	BHLHE40	-0.7517	1.27E-06
ENSG00000136040.9	PLXNC1	-0.755424	3.82E-14
ENSG00000175175.6	PPM1E	-0.765726	2.54E-05
ENSG00000133055.9	MYBPH	-0.768559	2.36E-03
ENSG00000155961.5	RAB39B	-0.775121	3.56E-06
ENSG00000160447.7	PKN3	-0.777045	2.59E-06
ENSG00000214265.11		-0.778679	6.40E-03
ENSG00000144785.8		-0.78208	1.38E-02

ENSG00000132932.17	ATP8A2	-0.790806	2.18E-03
ENSG00000120057.5	SFRP5	-0.796131	2.04E-52
ENSG00000168646.13	AXIN2	-0.803703	1.92E-04
ENSG00000251503.8	CENPS-CORT	-0.812444	2.73E-03
ENSG00000168209.5	DDIT4	-0.816176	1.31E-05
ENSG00000117707.16	PROX1	-0.82564	3.25E-05
ENSG00000162676.12	GFI1	-0.826553	1.70E-06
ENSG00000182010.11	RTKN2	-0.842695	3.97E-13
ENSG00000104921.15	FCER2	-0.851017	8.90E-06
ENSG00000110076.19	NRXN2	-0.872457	5.92E-09
ENSG00000179241.13	LDLRAD3	-0.886166	2.33E-14
ENSG00000285953.1		-0.891675	1.75E-03
ENSG00000172995.16	ARPP21	-0.892965	4.46E-04
ENSG00000165804.16	ZNF219	-0.893307	2.05E-12
ENSG00000147852.16	VLDLR	-0.896066	1.24E-20
ENSG00000157927.17	RADIL	-0.898267	2.72E-03
ENSG00000080709.16	KCNN2	-0.905142	9.00E-11
ENSG00000189367.15	KIAA0408	-0.910681	2.72E-02
ENSG00000274429.2	DLG5	-0.915907	2.68E-06
ENSG00000170681.7	CAVIN4	-0.917534	1.42E-02
ENSG00000153944.11	MSI2	-0.925795	1.37E-14
ENSG00000129993.15	CBFA2T3	-0.931168	4.61E-08
ENSG00000132718.9	SYT11	-0.938172	1.17E-07
ENSG00000124493.14	GRM4	-0.942201	8.04E-04
ENSG00000196358.11	NTNG2	-0.944373	2.95E-02
ENSG00000185909.15	KLHDC8B	-0.950642	8.78E-03
ENSG00000256663.1		-0.954987	1.77E-06
ENSG00000130222.11	GADD45G	-0.958376	5.14E-05
ENSG00000153208.17	MERTK	-0.958418	2.76E-06
ENSG00000229212.8		-0.983434	9.70E-03
ENSG00000165409.17	TSHR	-0.983658	1.57E-07
ENSG00000143153.13	ATP1B1	-1.006986	2.70E-02
ENSG00000103888.17	CEMIP	-1.018323	1.33E-05
ENSG00000185100.10	ADSS1	-1.020264	9.51E-03
ENSG00000137101.13	CD72	-1.020735	9.82E-17
ENSG00000158164.7	TMSB15A	-1.034041	1.13E-06
ENSG00000205336.13	ADGRG1	-1.037078	1.48E-03
ENSG00000128322.7	IGLL1	-1.037675	4.89E-15
ENSG00000046653.15	GPM6B	-1.040479	5.39E-04
ENSG00000168528.12	SERINC2	-1.041444	1.22E-04

ENSG00000163576.18	EFHB	-1.043127	1.94E-02
ENSG00000155966.14	AFF2	-1.050159	2.83E-13
ENSG00000267534.4	S1PR2	-1.052139	1.05E-21
ENSG00000133142.17	TCEAL4	-1.05449	3.53E-03
ENSG00000251349.3	MSANTD3-TMEFF1	-1.057434	1.18E-12
ENSG00000143889.16	HNRNPLL	-1.081362	2.27E-09
ENSG00000176406.23	RIMS2	-1.096787	3.25E-17
ENSG00000100504.17	PYGL	-1.110037	1.01E-14
ENSG00000123358.20	NR4A1	-1.115395	8.70E-19
ENSG00000181826.10	RELL1	-1.13816	2.83E-13
ENSG00000100154.14	TTC28	-1.155097	7.20E-03
ENSG00000160145.15	KALRN	-1.158196	1.62E-02
ENSG00000165801.10	ARHGEF40	-1.174894	6.05E-11
ENSG00000128886.12	ELL3	-1.189314	3.47E-09
ENSG00000250510.8	GPR162	-1.191031	8.57E-03
ENSG00000241697.5	TMEFF1	-1.194401	2.25E-05
ENSG00000177181.15	RIMKLA	-1.21235	9.49E-06
ENSG00000187122.17	SLIT1	-1.243718	5.47E-22
ENSG00000135605.13	TEC	-1.243726	2.71E-06
ENSG00000161835.11	GRASP	-1.264069	5.82E-03
ENSG00000185483.12	ROR1	-1.268433	1.06E-19
ENSG00000138356.14	AOX1	-1.284901	1.13E-02
ENSG00000177425.11	PAWR	-1.311433	1.93E-03
ENSG00000240771.8	ARHGEF25	-1.315896	1.46E-07
ENSG00000142583.18	SLC2A5	-1.370217	1.18E-12
ENSG00000160191.18	PDE9A	-1.418987	2.37E-16
ENSG00000143507.18	DUSP10	-1.419038	1.45E-18
ENSG00000140848.17	CPNE2	-1.448801	1.71E-18
ENSG00000129048.7	ACKR4	-1.452845	1.37E-07
ENSG00000149212.12	SESN3	-1.574672	2.49E-03
ENSG00000155093.19	PTPRN2	-1.594685	2.61E-10
ENSG00000069702.11	TGFBR3	-1.624086	4.08E-03
ENSG00000134323.12	MYCN	-1.632898	2.34E-03
ENSG00000196569.12	LAMA2	-1.652548	2.33E-04
ENSG00000105810.10	CDK6	-1.727766	1.90E-13
ENSG00000112474.4	HSD17B8	-1.748415	3.09E-03
ENSG00000168502.17	MTCL1	-1.788375	2.14E-12
ENSG00000260386.6	LDC1P	-1.791277	2.11E-05
ENSG00000112290.13	WASF1	-1.862236	2.53E-29

ENSG00000104879.5	CKM	-1.898658	3.48E-24
ENSG00000023171.18	GRAMD1B	-1.900824	6.20E-08
ENSG00000198467.16	TPM2	-2.031831	1.41E-34
ENSG00000244301.6	AOX3P	-2.129299	1.72E-05
ENSG00000243478.9	AOX2P	-2.208442	1.27E-06
ENSG00000276977.3	PDCD1	-2.381312	2.51E-04
ENSG00000079156.17	OSBPL6	-3.095049	2.04E-05

FC: Fold Change; adj.P.Val: adjusted P-value

Table S10. Gene sets used for gene set enrichment analysis and GO analysis containing differentially expressed genes upregulated and downregulated in Z138 Ro 08-2750 vs Z138 DMSO treated cells (genes with an adjusted P-value <0.1 and absolute log_2-transformed fold change >0.65).

**RO_UPREGULATED _GENES**		**RO_DOWNREGULATED_GENES**	
GeneID	Gene Symbol	GeneID	Gene Symbol
ENSG00000130513	GDF15	ENSG00000106624	AEBP1
ENSG00000090104	RGS1	ENSG00000100413	POLR3H
ENSG00000170345	FOS	ENSG00000103245	CIAO3
ENSG00000059804	SLC2A3	ENSG00000173540	GMPPB
ENSG00000254806	SYS1-DBNDD2	ENSG00000072571	HMMR
ENSG00000170689	HOXB9	ENSG00000181826	RELL1
ENSG00000120875	DUSP4	ENSG00000171033	PKIA
ENSG00000138166	DUSP5	ENSG00000099953	MMP11
ENSG00000117425	PTCH2	ENSG00000100592	DAAM1
ENSG00000087074	PPP1R15A	ENSG00000141503	MINK1
ENSG00000140961	OSGIN1	ENSG00000133627	ACTR3B
ENSG00000128965	CHAC1	ENSG00000167771	RCOR2
ENSG00000277632	CCL3	ENSG00000175970	UNC119B
ENSG00000113739	STC2	ENSG00000164442	CITED2
ENSG00000143507	DUSP10	ENSG00000113643	RARS1
ENSG00000175197	DDIT3	ENSG00000176749	CDK5R1
ENSG00000139269	INHBE	ENSG00000136305	CIDEB
ENSG00000185022	MAFF	ENSG00000285199	CIDEB
ENSG00000156804	FBXO32	ENSG00000178295	GEN1
ENSG00000130766	SESN2	ENSG00000100422	CERK
ENSG00000285069	SESN2	ENSG00000185811	IKZF1
ENSG00000163545	NUAK2	ENSG00000215041	NEURL4
ENSG00000185262	UBALD2	ENSG00000288301	NEURL4
ENSG00000183696	UPP1	ENSG00000205726	ITSN1
ENSG00000168209	DDIT4	ENSG00000145604	SKP2
ENSG00000162772	ATF3	ENSG00000110931	CAMKK2
ENSG00000263006	ROCK1P1	ENSG00000213347	MXD3
ENSG00000269378		ENSG00000171970	ZNF57
ENSG00000138670	RASGEF1B	ENSG00000075711	DLG1
ENSG00000138161	CUZD1	ENSG00000127325	BEST3
ENSG00000100439	ABHD4	ENSG00000126107	HECTD3
ENSG00000116717	GADD45A	ENSG00000138074	SLC5A6

ENSG00000134531	EMP1	ENSG00000227051	C14orf132
ENSG00000100292	HMOX1	ENSG00000235374	SSR4P1
ENSG00000157514	TSC22D3	ENSG00000015532	XYLT2
ENSG00000198369	SPRED2	ENSG00000174945	AMZ1
ENSG00000125740	FOSB	ENSG00000148773	MKI67
ENSG00000140044	JDP2	ENSG00000224501	HSPA1B
ENSG00000164463	CREBRF	ENSG00000274287	SCRIB
ENSG00000151012	SLC7A11	ENSG00000223756	TSSC2
ENSG00000167550	RHEBL1	ENSG00000128694	OSGEPL1
ENSG00000110934	BIN2	ENSG00000281178	MLXIP
ENSG00000285130		ENSG00000162073	PAQR4
ENSG00000162512	SDC3	ENSG00000135925	WNT10A
ENSG00000104490	NCALD	ENSG00000118965	WDR35
ENSG00000111981	ULBP1	ENSG00000161547	SRSF2
ENSG00000175697	GPR156	ENSG00000143515	ATP8B2
ENSG00000122224	LY9	ENSG00000070961	ATP2B1
ENSG00000026103	FAS	ENSG00000124357	NAGK
ENSG00000105327	BBC3	ENSG00000188157	AGRN
ENSG00000167723	TRPV3	ENSG00000196678	ERI2
ENSG00000278567	CCL3	ENSG00000134138	MEIS2
ENSG00000174307	PHLDA3	ENSG00000064687	ABCA7
ENSG00000183779	ZNF703	ENSG00000171206	TRIM8
ENSG00000146278	PNRC1	ENSG00000158050	DUSP2
ENSG00000116741	RGS2	ENSG00000137845	ADAM10
ENSG00000172216	CEBPB	ENSG00000100104	SRRD
ENSG00000105856	HBP1	ENSG00000040531	CTNS
ENSG00000283847	HBP1	ENSG00000147862	NFIB
ENSG00000101255	TRIB3	ENSG00000160654	CD3G
ENSG00000139112	GABARAPL1	ENSG00000127585	FBXL16
ENSG00000277406	SEC22B4P	ENSG00000128394	APOBEC3F
ENSG00000185495		ENSG00000180787	ZFP3
ENSG00000134107	BHLHE40	ENSG00000178921	PFAS
ENSG00000099954	CECR2	ENSG00000167536	DHRS13
ENSG00000186591	UBE2H	ENSG00000196922	ZNF252P
ENSG00000128165	ADM2	ENSG00000274675	GTF2H2C_2
ENSG00000139946	PELI2	ENSG00000184557	SOCS3
ENSG00000114796	KLHL24	ENSG00000135637	CCDC142
ENSG00000153443	UBALD1	ENSG00000181038	METTL23
ENSG00000177606	JUN	ENSG00000124635	H2BC11
ENSG00000103522	IL21R	ENSG00000175048	ZDHHC14
ENSG00000185950	IRS2	ENSG00000196787	H2AC11

ENSG00000133874	RNF122	ENSG00000115084	SLC35F5
ENSG00000138764	CCNG2	ENSG00000241015	TPM3P9
ENSG00000123358	NR4A1	ENSG00000188906	LRRK2
ENSG00000280962	DUSP16	ENSG00000162062	TEDC2
ENSG00000111266	DUSP16	ENSG00000112079	STK38
ENSG00000260916	CCPG1	ENSG00000176055	MBLAC2
ENSG00000175592	FOSL1	ENSG00000157350	ST3GAL2
ENSG00000166046	TCP11L2	ENSG00000164880	INTS1
ENSG00000120889	TNFRSF10B	ENSG00000282733	NSFP1
ENSG00000182459	TEX19	ENSG00000169710	FASN
ENSG00000118515	SGK1	ENSG00000101265	RASSF2
ENSG00000105204	DYRK1B	ENSG00000066279	ASPM
ENSG00000281320	DYRK1B	ENSG00000080200	CRYBG3
ENSG00000274221	CCL3	ENSG00000213222	TOMM40P4
ENSG00000175155	YPEL2	ENSG00000281848	TOMM40P4
ENSG00000218358	RAET1K	ENSG00000165521	EML5
ENSG00000067082	KLF6	ENSG00000116005	PCYOX1
ENSG00000104497	SNX16	ENSG00000215271	HOMEZ
ENSG00000174564	IL20RB	ENSG00000135439	AGAP2
ENSG00000178562	CD28	ENSG00000188827	SLX4
ENSG00000269028	MTRNR2L12	ENSG00000137124	ALDH1B1
ENSG00000115956	PLEK	ENSG00000227268	KLLN
ENSG00000148339	SLC25A25	ENSG00000168135	KCNJ4
ENSG00000051108	HERPUD1	ENSG00000119866	BCL11A
ENSG00000255823	MTRNR2L8	ENSG00000177272	KCNA3
ENSG00000204070	SYS1	ENSG00000185504	FAAP100
ENSG00000232196	MTRNR2L4	ENSG00000211675	IGLC1
ENSG00000170222	ADPRM	ENSG00000160606	TLCD1
ENSG00000156232	WHAMM	ENSG00000174236	REP15
ENSG00000225828	FAM229A	ENSG00000276833	TAF15
ENSG00000135540	NHSL1	ENSG00000180900	SCRIB
ENSG00000151687	ANKAR	ENSG00000168646	AXIN2
ENSG00000156976	EIF4A2	ENSG00000072694	FCGR2B
ENSG00000112406	HECA	ENSG00000187808	SOWAHD
ENSG00000119801	YPEL5	ENSG00000076351	SLC46A1
ENSG00000135241	PNPLA8	ENSG00000110697	PITPNM1
ENSG00000130449	ZSWIM6	ENSG00000127603	MACF1
ENSG00000175189	INHBC	ENSG00000143315	PIGM
ENSG00000148842	CNNM2	ENSG00000153896	ZNF599
ENSG00000183307	TMEM121B	ENSG00000254709	IGLL5
ENSG00000278062	ZNF251	ENSG00000177380	PPFIA3

ENSG00000169155	ZBTB43	ENSG00000184508	HDDC3
ENSG00000146232	NFKBIE	ENSG00000206535	LNP1
ENSG00000271303	SRXN1	ENSG00000103707	MTFMT
ENSG00000256618	MTRNR2L1	ENSG00000143126	CELSR2
ENSG00000137502	RAB30	ENSG00000161940	BCL6B
ENSG00000256045	MTRNR2L10	ENSG00000162688	AGL
ENSG00000132003	ZSWIM4	ENSG00000205476	CCDC85C
ENSG00000288360	ZSWIM4	ENSG00000275079	LPCAT1
ENSG00000164949	GEM	ENSG00000144455	SUMF1
ENSG00000141682	PMAIP1	ENSG00000167114	SLC27A4
ENSG00000161011	SQSTM1	ENSG00000175040	CHST2
ENSG00000138835	RGS3	ENSG00000118894	EEF2KMT
ENSG00000187486	KCNJ11	ENSG00000196664	TLR7
ENSG00000164603	BMT2	ENSG00000143971	ETAA1
ENSG00000086619	ERO1B	ENSG00000104356	POP1
ENSG00000277773	SMN2	ENSG00000166965	RCCD1
ENSG00000168003	SLC3A2	ENSG00000105429	MEGF8
ENSG00000223865	HLA-DPB1	ENSG00000117215	PLA2G2D
ENSG00000053524	MCF2L2	ENSG00000177409	SAMD9L
ENSG00000165121		ENSG00000160539	PLPP7
ENSG00000147852	VLDLR	ENSG00000137078	SIT1
ENSG00000170385	SLC30A1	ENSG00000168936	TMEM129
ENSG00000188522	FAM83G	ENSG00000285953	
ENSG00000083828	ZNF586	ENSG00000211644	IGLV1-51
ENSG00000204520	MICA	ENSG00000128274	A4GALT
ENSG00000118308	LRMP	ENSG00000160957	RECQL4
ENSG00000059728	MXD1	ENSG00000140104	CLBA1
ENSG00000110042	DTX4	ENSG00000240204	SMKR1
ENSG00000108306	FBXL20	ENSG00000176108	CHMP6
ENSG00000104221	BRF2	ENSG00000184898	RBM43
ENSG00000132823	OSER1	ENSG00000157184	CPT2
ENSG00000282228	PAM16	ENSG00000285253	
ENSG00000067182	TNFRSF1A	ENSG00000264058	
ENSG00000168916	ZNF608	ENSG00000275835	TUBGCP5
ENSG00000173575	CHD2	ENSG00000108219	TSPAN14
ENSG00000111897	SERINC1	ENSG00000204569	PPP1R10
ENSG00000198886	MT-ND4	ENSG00000185875	THNSL1
ENSG00000160789	LMNA	ENSG00000138131	LOXL4
ENSG00000270188	MTRNR2L11	ENSG00000117643	MAN1C1
ENSG00000212907	MT-ND4L	ENSG00000166317	SYNPO2L
ENSG00000105649	RAB3A	ENSG00000269001	

ENSG00000267127		ENSG00000188042	ARL4C
ENSG00000197063	MAFG	ENSG00000196659	TTC30B
ENSG00000109220	CHIC2	ENSG00000168268	NT5DC2
ENSG00000155961	RAB39B	ENSG00000102384	CENPI
ENSG00000113734	BNIP1	ENSG00000100241	SBF1
ENSG00000104689	TNFRSF10A	ENSG00000196227	FAM217B
ENSG00000175105	ZNF654	ENSG00000186350	RXRA
ENSG00000111011	RSRC2	ENSG00000184574	LPAR5
ENSG00000258555	SPECC1L-ADORA2A	ENSG00000188486	H2AX
ENSG00000175866	BAIAP2	ENSG00000259529	
ENSG00000154359	LONRF1	ENSG00000284845	
ENSG00000173812	EIF1	ENSG00000155265	GOLGA7B
ENSG00000206493	HLA-E	ENSG00000269404	SPIB
ENSG00000229252	HLA-E	ENSG00000146966	DENND2A
ENSG00000230254	HLA-E	ENSG00000230797	YY2
ENSG00000233904	HLA-E	ENSG00000158473	CD1D
ENSG00000102096	PIM2	ENSG00000175283	DOLK
ENSG00000157764	BRAF	ENSG00000198908	BHLHB9
ENSG00000198925	ATG9A	ENSG00000198752	CDC42BPB
ENSG00000228409	CCT6P1	ENSG00000162746	FCRLB
ENSG00000282641		ENSG00000157240	FZD1
ENSG00000112715	VEGFA	ENSG00000129521	EGLN3
ENSG00000166068	SPRED1	ENSG00000213638	ADAT3
ENSG00000172081	MOB3A	ENSG00000136295	TTYH3
ENSG00000152409	JMY	ENSG00000150756	ATPSCKMT
ENSG00000135116	HRK	ENSG00000198743	SLC5A3
ENSG00000196517	SLC6A9	ENSG00000189057	FAM111B
ENSG00000160223	ICOSLG	ENSG00000104983	CCDC61
ENSG00000226329		ENSG00000136514	RTP4
ENSG00000105717	PBX4	ENSG00000171604	CXXC5
ENSG00000256683	ZNF350	ENSG00000261716	H2BC20P
ENSG00000270672	MTRNR2L6	ENSG00000125703	ATG4C
ENSG00000120910	PPP3CC	ENSG00000112293	GPLD1
ENSG00000163584	RPL22L1	ENSG00000108175	ZMIZ1
ENSG00000285360	LONRF1	ENSG00000146802	TMEM168
ENSG00000162616	DNAJB4	ENSG00000144120	TMEM177
ENSG00000275618	BFAR	ENSG00000275074	NUDT18
ENSG00000277117		ENSG00000173898	SPTBN2
ENSG00000168264	IRF2BP2	ENSG00000173208	ABCD2
ENSG00000082641	NFE2L1	ENSG00000127528	KLF2
ENSG00000173875	ZNF791	ENSG00000278195	SSTR3

ENSG00000225553	PHF1	ENSG00000198840	MT-ND3
ENSG00000220804	LINC01881	ENSG00000115884	SDC1
ENSG00000144481	TRPM8	ENSG00000169668	BCRP2
ENSG00000285816		ENSG00000170469	SPATA24
ENSG00000164236	ANKRD33B	ENSG00000163873	GRIK3
ENSG00000087266	SH3BP2	ENSG00000148400	NOTCH1
ENSG00000115548	KDM3A	ENSG00000176444	CLK2
ENSG00000010818	HIVEP2	ENSG00000166750	SLFN5
ENSG00000178607	ERN1	ENSG00000273604	EPOP
ENSG00000130518	IQCN	ENSG00000141642	ELAC1
ENSG00000168255	POLR2J3	ENSG00000105974	CAV1
ENSG00000165030	NFIL3	ENSG00000237651	C2orf74
ENSG00000100784	RPS6KA5	ENSG00000183496	MEX3B
ENSG00000257390		ENSG00000117707	PROX1
ENSG00000143333	RGS16	ENSG00000107816	LZTS2
ENSG00000178338	ZNF354B	ENSG00000134954	ETS1
ENSG00000065809	FAM107B	ENSG00000174885	NLRP6
ENSG00000118496	FBXO30	ENSG00000129480	DTD2
ENSG00000124224	PPP4R1L	ENSG00000198890	PRMT6
ENSG00000124762	CDKN1A	ENSG00000204851	PNMA8B
ENSG00000104856	RELB	ENSG00000142224	IL19
ENSG00000188033	ZNF490	ENSG00000165417	GTF2A1
ENSG00000165113	GKAP1	ENSG00000126705	AHDC1
ENSG00000166900	STX3	ENSG00000164850	GPER1
ENSG00000162373	BEND5	ENSG00000187079	TEAD1
ENSG00000135999	EPC2	ENSG00000149679	CABLES2
ENSG00000264522	OTUD7B	ENSG00000197256	KANK2
ENSG00000126561	STAT5A	ENSG00000284691	
ENSG00000167615	LENG8	ENSG00000271741	
ENSG00000160908	ZNF394	ENSG00000099814	CEP170B
ENSG00000231247	PPP1R18	ENSG00000096746	HNRNPH3
ENSG00000182324	KCNJ14	ENSG00000184709	LRRC26
ENSG00000184897	H1-10	ENSG00000162341	TPCN2
ENSG00000136504	KAT7	ENSG00000158483	FAM86C1
ENSG00000141391	PRELID3A	ENSG00000174516	PELI3
ENSG00000052802	MSMO1	ENSG00000179588	ZFPM1
ENSG00000121577	POPDC2	ENSG00000075240	GRAMD4
ENSG00000220205	VAMP2	ENSG00000175054	ATR
ENSG00000105281	SLC1A5	ENSG00000184524	CEND1
ENSG00000143622	RIT1	ENSG00000273814	OCLN
ENSG00000024862	CCDC28A	ENSG00000176909	MAMSTR

ENSG00000185436	IFNLR1	ENSG00000275600	PIGW
ENSG00000165879	FRAT1	ENSG00000130487	KLHDC7B
ENSG00000198938	MT-CO3	ENSG00000235750	KIAA0040
ENSG00000121879	PIK3CA	ENSG00000177352	CCDC71
ENSG00000117091	CD48	ENSG00000171617	ENC1
ENSG00000198625	MDM4	ENSG00000239732	TLR9
ENSG00000196705	ZNF431	ENSG00000186666	BCDIN3D
ENSG00000127081	ZNF484	ENSG00000211640	IGLV6-57
ENSG00000065911	MTHFD2	ENSG00000176410	DNAJC30
ENSG00000076053	RBM7	ENSG00000160326	SLC2A6
ENSG00000182831	C16orf72	ENSG00000281165	SLC2A6
ENSG00000159556	ISL2	ENSG00000081692	JMJD4
ENSG00000179361	ARID3B	ENSG00000101246	ARFRP1
ENSG00000188215	DCUN1D3	ENSG00000101447	FAM83D
ENSG00000078804	TP53INP2	ENSG00000144824	PHLDB2
ENSG00000080546	SESN1	ENSG00000180626	ZNF594
ENSG00000198888	MT-ND1	ENSG00000135622	SEMA4F
ENSG00000168884	TNIP2	ENSG00000154864	PIEZO2
ENSG00000197019	SERTAD1	ENSG00000114554	PLXNA1
ENSG00000198899	MT-ATP6	ENSG00000112146	FBXO9
ENSG00000284981	UPK3BL2	ENSG00000197183	NOL4L
ENSG00000273559	CWC25	ENSG00000130021	PUDP
ENSG00000170631	ZNF16	ENSG00000182584	ACTL10
ENSG00000286098		ENSG00000277161	PIGW
ENSG00000234585	CCT6P3	ENSG00000198416	ZNF658B
ENSG00000267561		ENSG00000125910	S1PR4
ENSG00000196267	ZNF836	ENSG00000179583	CIITA
ENSG00000113328	CCNG1	ENSG00000153395	LPCAT1
ENSG00000283515		ENSG00000123080	CDKN2C
ENSG00000284099		ENSG00000183060	LYSMD4
ENSG00000204524	ZNF805	ENSG00000169884	WNT10B
ENSG00000197885	NKIRAS1	ENSG00000106689	LHX2
ENSG00000169239	CA5B	ENSG00000135547	HEY2
ENSG00000137955	RABGGTB	ENSG00000177628	GBA
ENSG00000204592	HLA-E	ENSG00000108786	HSD17B1
ENSG00000237973	MTCO1P12	ENSG00000145882	PCYOX1L
ENSG00000100201	DDX17	ENSG00000197471	SPN
ENSG00000142279	WTIP	ENSG00000175305	CCNE2
ENSG00000163635	ATXN7	ENSG00000259330	INAFM2
ENSG00000179454	KLHL28	ENSG00000156970	BUB1B
ENSG00000107731	UNC5B	ENSG00000266074	BAHCC1

ENSG00000059758	CDK17	ENSG00000203883	SOX18
ENSG00000120071	KANSL1	ENSG00000185669	SNAI3
ENSG00000005471	ABCB4	ENSG00000251669	FAM86EP
ENSG00000139289	PHLDA1	ENSG00000144136	SLC20A1
ENSG00000156675	RAB11FIP1	ENSG00000126803	HSPA2
ENSG00000011566	MAP4K3	ENSG00000139174	PRICKLE1
ENSG00000164543	STK17A	ENSG00000274349	ZNF658
ENSG00000228253	MT-ATP8	ENSG00000262446	GBA
ENSG00000259305	ZHX1-C8orf76	ENSG00000117461	PIK3R3
ENSG00000113595	TRIM23	ENSG00000128923	MINDY2
ENSG00000088876	ZNF343	ENSG00000276977	PDCD1
ENSG00000273562	NAP1L4	ENSG00000127863	TNFRSF19
ENSG00000185947	ZNF267	ENSG00000178409	BEND3
ENSG00000151881	TMEM267	ENSG00000160991	ORAI2
ENSG00000139514	SLC7A1	ENSG00000144488	ESPNL
ENSG00000126821	SGPP1	ENSG00000198838	RYR3
ENSG00000285281	SGPP1	ENSG00000168792	ABHD15
ENSG00000224608	HLA-B	ENSG00000108590	MED31
ENSG00000231259	ANAPC1P2	ENSG00000215105	TTC3P1
ENSG00000115289	PCGF1	ENSG00000228672	PROB1
ENSG00000248527	MTATP6P1	ENSG00000171365	CLCN5
ENSG00000198727	MT-CYB	ENSG00000123384	LRP1
ENSG00000109046	WSB1	ENSG00000070159	PTPN3
ENSG00000151917	BEND6	ENSG00000176678	FOXL1
ENSG00000150593	PDCD4	ENSG00000197056	ZMYM1
ENSG00000095951	HIVEP1	ENSG00000124766	SOX4
ENSG00000176407	KCMF1	ENSG00000198435	NRARP
ENSG00000178229	ZNF543	ENSG00000169683	LRRC45
ENSG00000107771	CCSER2	ENSG00000197557	TTC30A
ENSG00000237296	SMG1P1	ENSG00000175414	ARL10
ENSG00000177105	RHOG	ENSG00000179431	FJX1
ENSG00000132680	KHDC4	ENSG00000174123	TLR10
ENSG00000119950	MXI1	ENSG00000173404	INSM1
ENSG00000006652	IFRD1	ENSG00000186193	SAPCD2
ENSG00000166439	RNF169	ENSG00000145214	DGKQ
ENSG00000068323	TFE3	ENSG00000167700	MFSD3
ENSG00000135069	PSAT1	ENSG00000148411	NACC2
ENSG00000015153	YAF2	ENSG00000172000	ZNF556
ENSG00000172687	ZNF738	ENSG00000186523	FAM86B1
ENSG00000142528	ZNF473	ENSG00000163032	VSNL1
ENSG00000115525	ST3GAL5	ENSG00000137802	MAPKBP1

ENSG00000147162	OGT	ENSG00000092758	COL9A3
ENSG00000224420	ADM5	ENSG00000170915	PAQR8
ENSG00000048649	RSF1	ENSG00000184185	KCNJ12
ENSG00000164615	CAMLG	ENSG00000173366	
ENSG00000123908	AGO2	ENSG00000120868	APAF1
ENSG00000164284	GRPEL2	ENSG00000188064	WNT7B
ENSG00000172243	CLEC7A	ENSG00000111913	RIPOR2
ENSG00000174738	NR1D2	ENSG00000126882	FAM78A
ENSG00000168488	ATXN2L	ENSG00000180340	FZD2
ENSG00000128272	ATF4	ENSG00000139537	CCDC65
ENSG00000118503	TNFAIP3	ENSG00000141736	ERBB2
ENSG00000103429	BFAR	ENSG00000273983	H3C8
ENSG00000197852	INKA2	ENSG00000188779	SKOR1
ENSG00000104899	AMH	ENSG00000204060	FOXO6
ENSG00000174132	FAM174A	ENSG00000216775	
ENSG00000174456	C12orf76	ENSG00000117877	CD3EAP
ENSG00000141034	GID4	ENSG00000233155	HMGA1P8
ENSG00000285901		ENSG00000180535	BHLHA15
ENSG00000166398	KIAA0355	ENSG00000186174	BCL9L
ENSG00000163602	RYBP	ENSG00000284308	C2orf81
ENSG00000270136	MICOS10-NBL1	ENSG00000155254	MARVELD1
ENSG00000140941	MAP1LC3B	ENSG00000204713	TRIM27
ENSG00000137575	SDCBP	ENSG00000184916	JAG2
ENSG00000113369	ARRDC3	ENSG00000169252	ADRB2
ENSG00000099725	PRKY	ENSG00000280893	
ENSG00000105643	ARRDC2	ENSG00000167074	TEF
ENSG00000123095	BHLHE41	ENSG00000196990	FAM163B
ENSG00000120616	EPC1	ENSG00000163485	ADORA1
ENSG00000277140	MARF1	ENSG00000095203	EPB41L4B
ENSG00000227232	WASH7P	ENSG00000169991	IFFO2
ENSG00000155304	HSPA13	ENSG00000273802	H2BC8
ENSG00000027697	IFNGR1	ENSG00000188613	NANOS1
ENSG00000023330	ALAS1	ENSG00000259660	DNM1P47
ENSG00000164180	TMEM161B	ENSG00000178401	DNAJC22
ENSG00000168826	ZBTB49	ENSG00000166897	ELFN2
ENSG00000119844	AFTPH	ENSG00000183208	GDPGP1
ENSG00000196352	CD55	ENSG00000108798	ABI3
ENSG00000100601	ALKBH1	ENSG00000177354	C10orf71
ENSG00000057657	PRDM1	ENSG00000184254	ALDH1A3
ENSG00000105613	MAST1	ENSG00000276657	PYCR3
ENSG00000132912	DCTN4	ENSG00000214357	NEURL1B

ENSG00000134324	LPIN1	ENSG00000172731	LRRC20
ENSG00000183484	GPR132	ENSG00000227804	PPP1R10
ENSG00000198839	ZNF277	ENSG00000230995	PPP1R10
ENSG00000130703	OSBPL2	ENSG00000231737	PPP1R10
ENSG00000038945	MSR1	ENSG00000235291	PPP1R10
ENSG00000156050	FAM161B	ENSG00000238104	PPP1R10
ENSG00000180385	EMC3-AS1	ENSG00000160172	FAM86C2P
ENSG00000188554	NBR1	ENSG00000186603	HPDL
ENSG00000090661	CERS4	ENSG00000187634	SAMD11
ENSG00000172007	RAB33B	ENSG00000165507	DEPP1
ENSG00000171791	BCL2	ENSG00000165801	ARHGEF40
ENSG00000157450	RNF111	ENSG00000155926	SLA
ENSG00000019995	ZRANB1	ENSG00000165548	TMEM63C
ENSG00000166123	GPT2	ENSG00000159208	CIART
ENSG00000198804	MT-CO1	ENSG00000182759	MAFA
ENSG00000276681	LENG8	ENSG00000137834	SMAD6
ENSG00000135913	USP37	ENSG00000106003	LFNG
ENSG00000140993	TIGD7	ENSG00000206489	PPP1R10
ENSG00000204178	MACO1	ENSG00000010319	SEMA3G
ENSG00000145365	TIFA	ENSG00000179627	ZBTB42
ENSG00000144566	RAB5A	ENSG00000180592	SKIDA1
ENSG00000099875	MKNK2	ENSG00000263160	TMEM50B
ENSG00000230961	MSH5	ENSG00000137135	ARHGEF39
ENSG00000172954	LCLAT1	ENSG00000211978	IGHV5-78
ENSG00000180530	NRIP1	ENSG00000276708	IGHV5-78
ENSG00000170871	KIAA0232	ENSG00000274750	H3C6
ENSG00000110324	IL10RA	ENSG00000128011	LRFN1
ENSG00000205534	SMG1P2	ENSG00000120280	CXorf21
ENSG00000185019	UBOX5	ENSG00000168906	MAT2A
ENSG00000081320	STK17B	ENSG00000104524	PYCR3
ENSG00000136158	SPRY2	ENSG00000171084	FAM86JP
ENSG00000198355	PIM3	ENSG00000136634	IL10
ENSG00000099860	GADD45B	ENSG00000128917	DLL4
ENSG00000140280	LYSMD2	ENSG00000162068	NTN3
		ENSG00000171388	APLN
		ENSG00000197705	KLHL14
		ENSG00000163449	TMEM169
		ENSG00000164849	GPR146
		ENSG00000125510	OPRL1
		ENSG00000253126	IGLVI-56

Table S11. Functional analysis on differentially expressed genes between Z138 Ro 08-2750 vs Z138 DMSO gene expression profile (genes with an adjusted P-value <0.1 and absolute log_2-transformed fold change >0.65) by using DAVID software.

Category	Term	Count	PValue	Fold Enrichment
GOTERM_BP_DIRECT	GO:0000122~negative regulation of transcription from RNA polymerase II promoter	30	2.2E-05	2.40
GOTERM_BP_DIRECT	GO:0060412~ventricular septum morphogenesis	6	1.3E-04	11.90
GOTERM_BP_DIRECT	GO:0060070~canonical Wnt signaling pathway	8	5.9E-04	5.54
GOTERM_BP_DIRECT	GO:0003151~outflow tract morphogenesis	6	1.2E-03	7.50
GOTERM_BP_DIRECT	GO:0008285~negative regulation of cell proliferation	17	1.6E-03	2.47
GOTERM_BP_DIRECT	GO:0045747~positive regulation of Notch signaling pathway	5	2.4E-03	8.71
BIOCARTA	h_hesPathway:Segmentation Clock	4	2.6E-03	13.27
KEGG_PATHWAY	hsa05217:Basal cell carcinoma	6	2.7E-03	6.11
GOTERM_BP_DIRECT	GO:0060022~hard palate development	3	4.3E-03	28.75
GOTERM_BP_DIRECT	GO:0003150~muscular septum morphogenesis	3	4.3E-03	28.75
GOTERM_BP_DIRECT	GO:0070059~intrinsic apoptotic signaling pathway in response to endoplasmic reticulum stress	10	3.4E-09	17.37
KEGG_PATHWAY	hsa04010:MAPK signaling pathway	19	9.4E-07	3.97
GOTERM_BP_DIRECT	GO:0000188~inactivation of MAPK activity	7	3.5E-06	16.05
KEGG_PATHWAY	hsa04115:p53 signaling pathway	10	3.7E-06	7.90
GOTERM_BP_DIRECT	GO:0006351~transcription, DNA-templated	60	1.5E-05	1.76
GOTERM_BP_DIRECT	GO:0006915~apoptotic process	26	2.0E-05	2.63
GOTERM_BP_DIRECT	GO:0034976~response to endoplasmic reticulum stress	9	4.7E-05	6.88
GOTERM_BP_DIRECT	GO:0045944~positive regulation of transcription from RNA polymerase II promoter	34	2.2E-04	1.99
KEGG_PATHWAY	hsa04650:Natural killer cell mediated cytotoxicity	10	4.4E-04	4.34
KEGG_PATHWAY	hsa04668:TNF signaling pathway	9	8.5E-04	4.45

SUPPLEMENTARY FIGURES

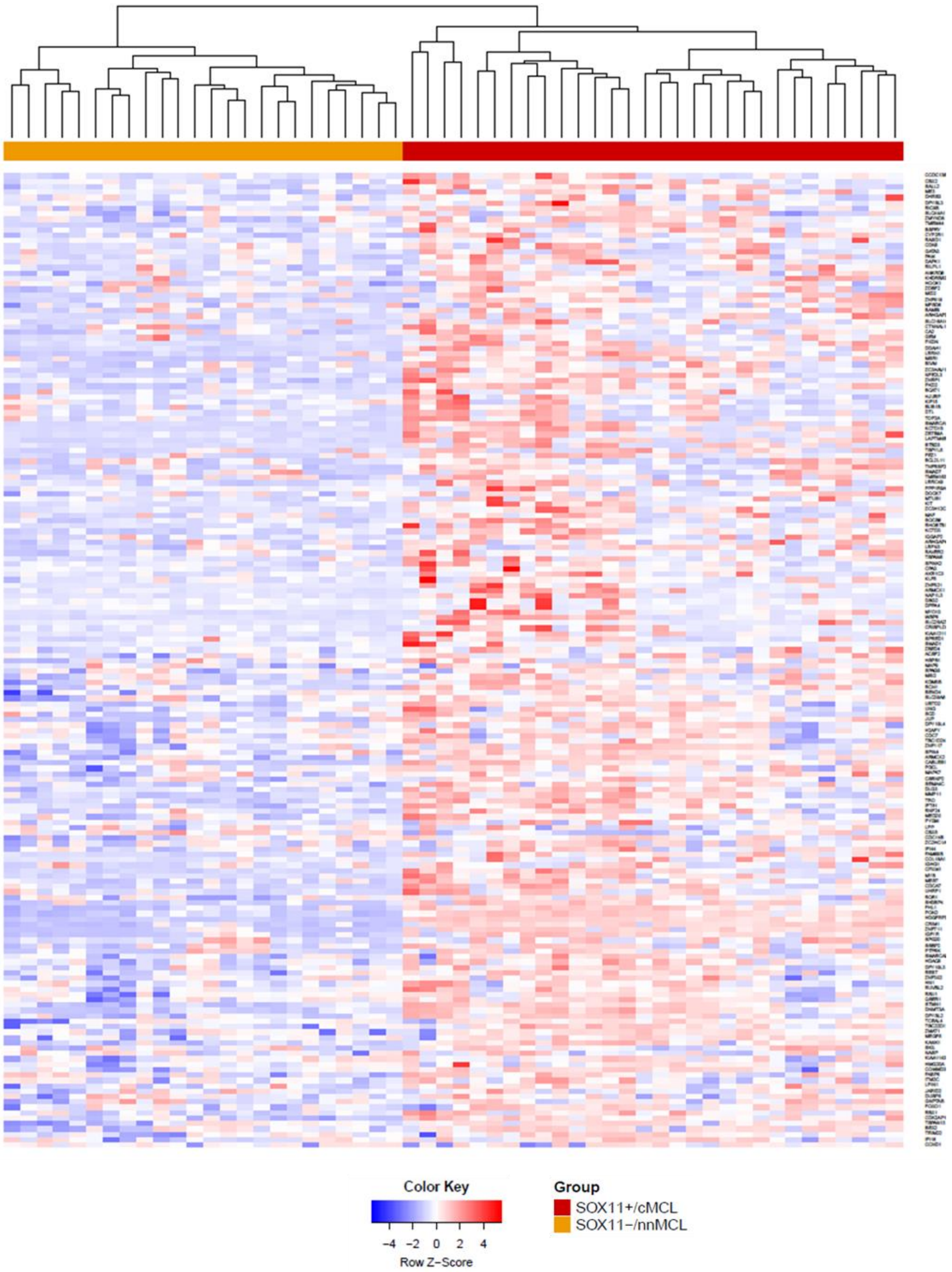

Fig. S1. Up-regulation of genes associated with stem cell functions in SOX11+ compared to SOX11- MCL primary cases. (A) Heatmap showing scaled expression of

181 significant upregulated genes from the top 4 gene sets related to stem cells (HSC-and LSC-related gene sets and NOS-target genes), enriched in SOX11+ (in red) vs. SOX11- (in orange) MCL primary cases (GSE79196), showed in Fig. 1A.

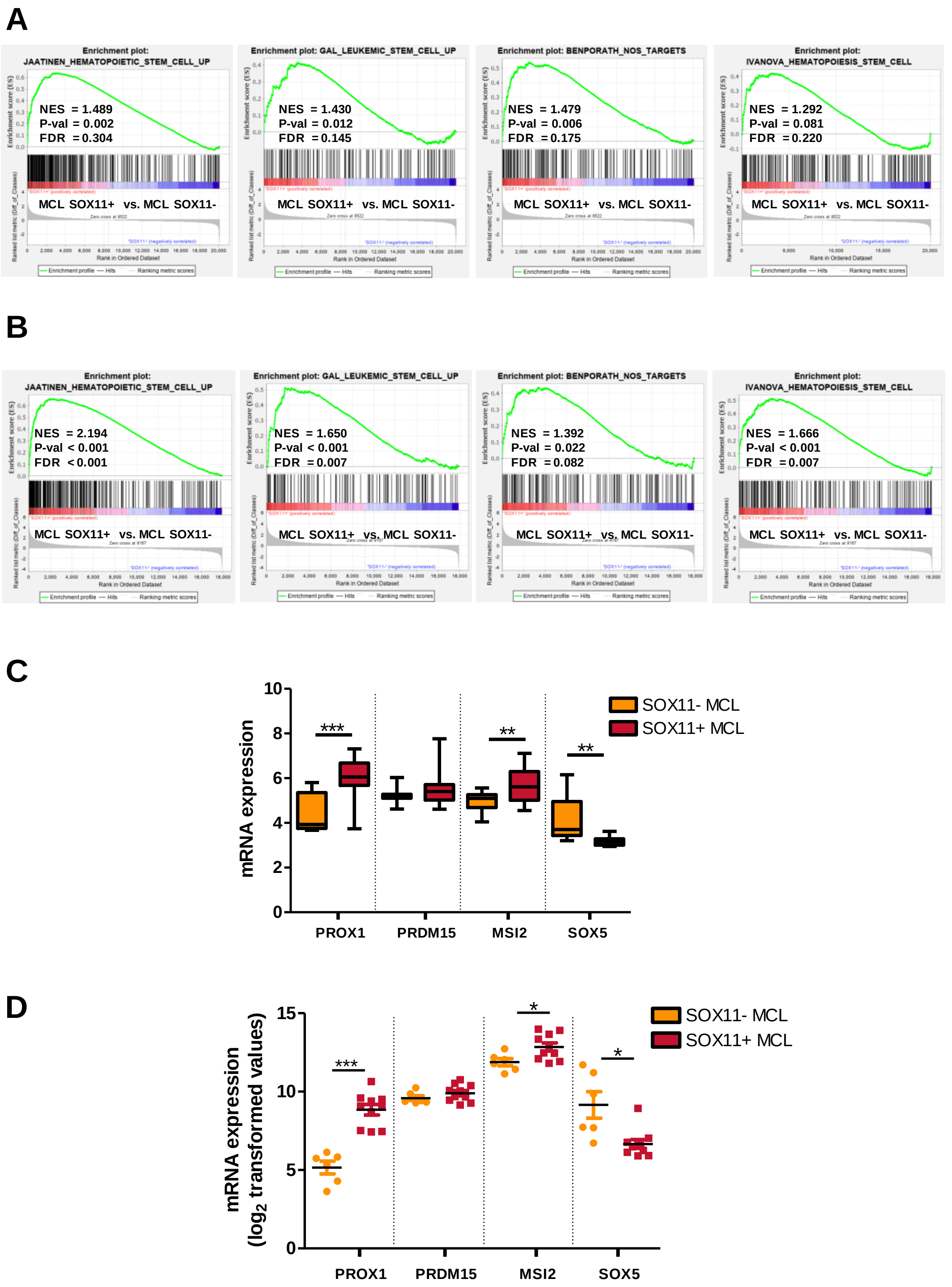

Fig. S2. Enrichment of stem cell related genes and signatures in SOX11+ MCL primary cases. Enrichment plots obtained by gene set enrichment analysis using gene

expression profile microarray data (EGAD00010001842) from 26 SOX11+ and 13 SOX11- **(A)** and RNA-seq data from 10 SOX11+ and 6 SOX11- **(B)** MCL primary tumors, showing significant enrichment of HSC, LSC and NOS target gene sets in $SOX11^{+}$. Normalized enrichment score (NES), P-value (P-val) and false discovery rate (FDR) are shown. **(C-D)** mRNA expression levels of PROX1, PRDM15, MSI2 and SOX5 in an independent cohort of 26 SOX11+ and 13 SOX11- **(C)**, and 10 SOX11+ and 6 SOX11- **(D)** MCL primary tumors, obtained by microarray and RNA-seq, respectively. The significance of difference was determined by unpaired two-tailed Student t-test (Welch's correction was applied to compare samples with different variances): * P-value <0.05, ** P-value <0.01, *** P-value <0.001.

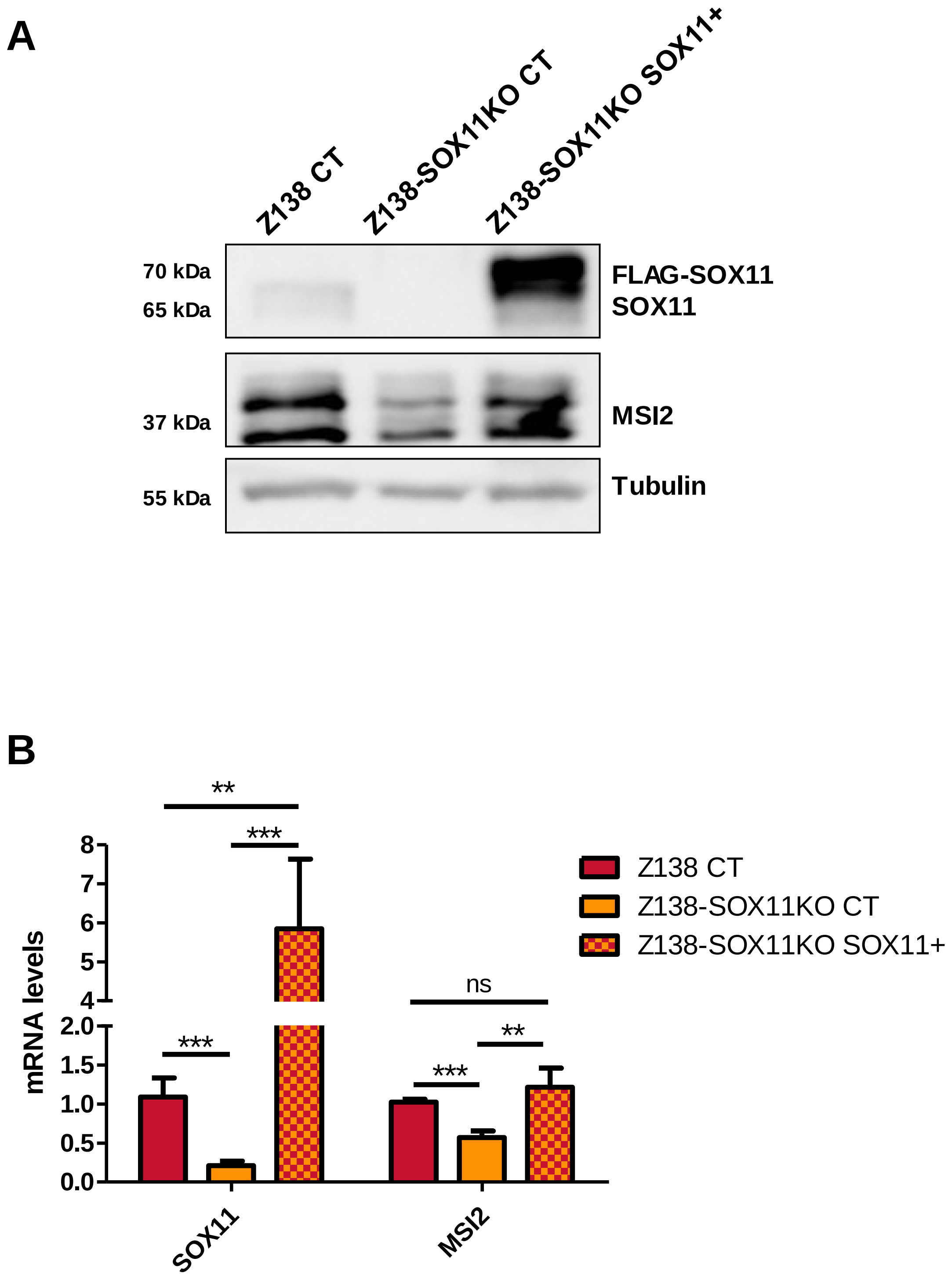

Fig. S3. Rescue of MSI2 mRNA and protein levels upon SOX11 overexpression in the Z138-SOX11KO MCL cell line. (A) SOX11 and MSI2 protein levels in Z138 WT, -SOX11KO CT and -SOX11KO SOX11+ (overexpressing FLAG- tagged SOX11 protein)

MCL cell lines. Tubulin was used as a loading control. **(B)** MSI2 and SOX11 mRNA levels, normalized to GUSB endogenous control (in fold change), in Z138 CT, Z138-SOX11KO CT and Z138-SOX11KO SOX11+. The mRNA levels are relative to Z138 CT and are represented as the mean ± standard deviation of 3 independent experiments. The significance of difference was determined by unpaired two-tailed Student t-test: ** P-value <0.01, *** P-value<0.001, ns = not significant.

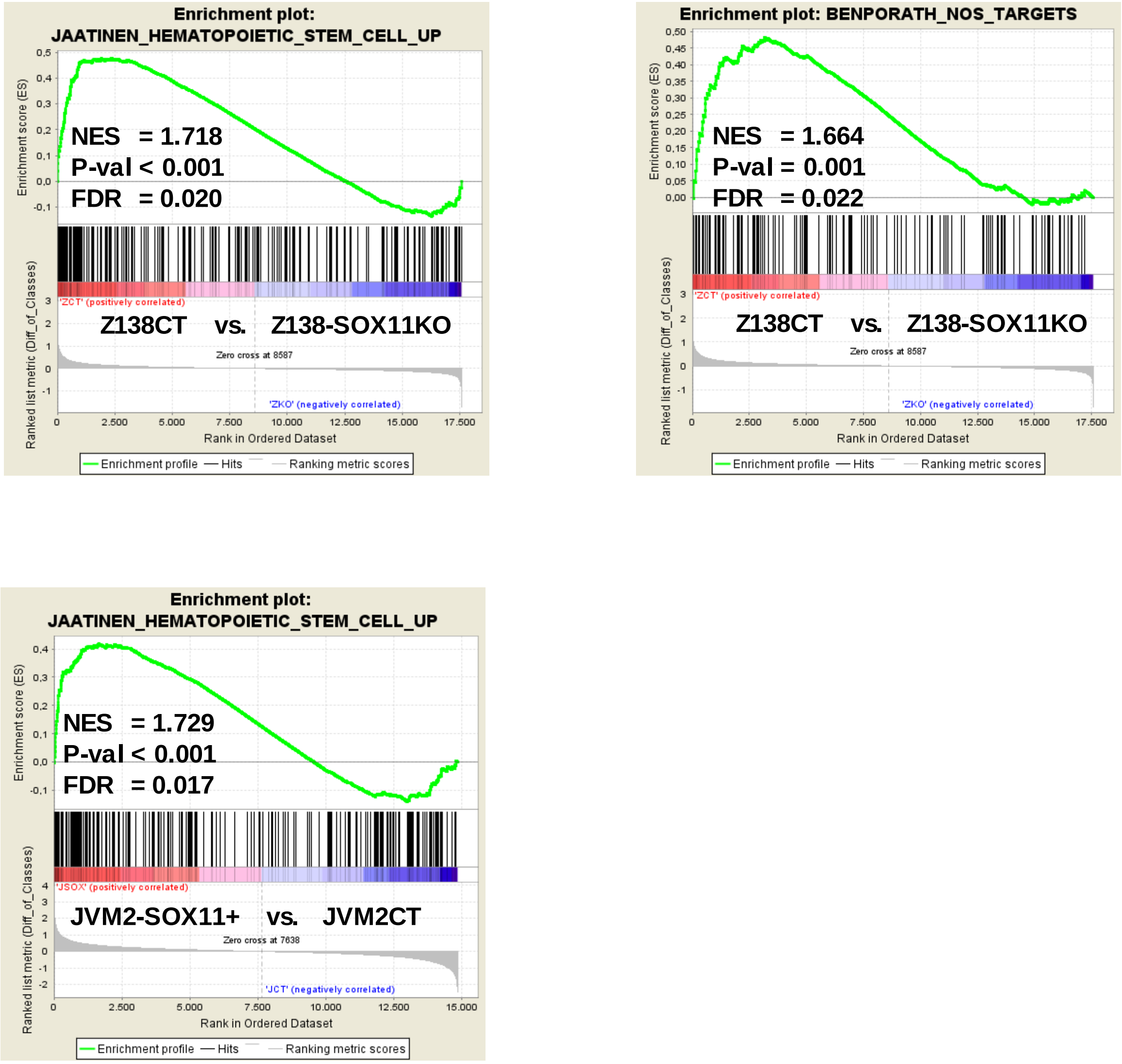

Fig. S4. Stem cell-related gene signatures enrichment in SOX11+ compared to SOX11- MCL cell lines. GSEA analysis using RNA-seq data from Z138CT vs Z138-SOX11KO, and JVM2CT vs JVM2-SOX11+ gene expression profiles on HSCs-related gene sets and NOS (pluripotent transcription factors Nanog, Oct4 and SOX2)-target genes. Normalized enrichment score (NES), P-value (P-val) and false discovery rate (FDR) are shown. Statistical significance is considered when FDR<0.2.

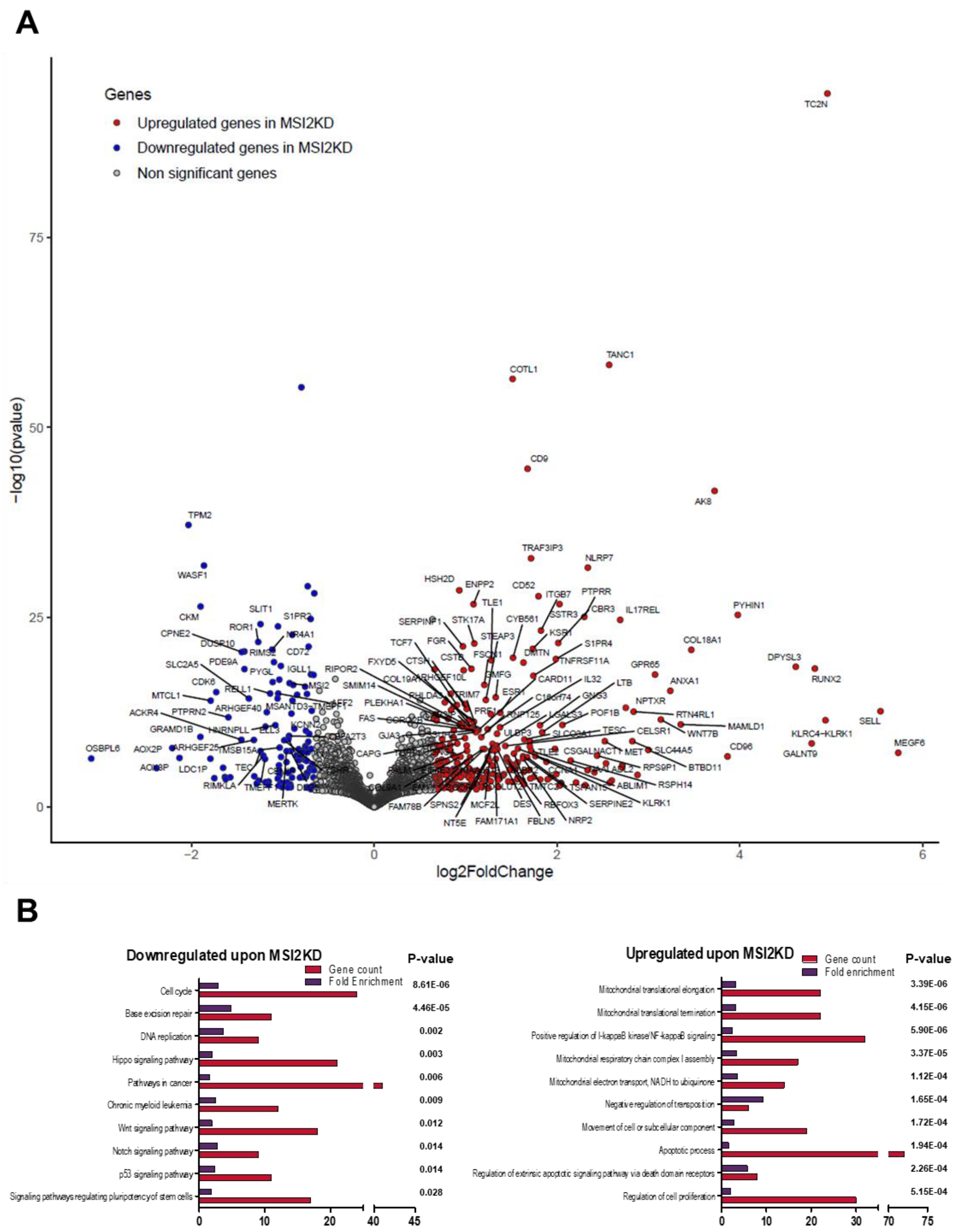

Fig. S5. Differentially expressed genes and dysregulated pathways upon MSI2KD in Z138 MCL cell line. (A) Volcano plot showing significant differentially expressed genes after MSI2KD in Z138 MCL cell line obtained by RNA-seq. The graph shows on the y-axis

-log (P-value) and on the x-axis the log_2-transformed fold change. Genes upregulated and downregulated in Z138MSI2KD vs Z138CT with an adjusted P-value <0.1 and log_2-transformed fold change >0.65 or <-0.65 are colored in red and blue, respectively, and genes with an adjusted P-value <0.00005 and absolute log_2-transformed fold change >0.9 are labeled with their Gene Symbol. **(B)** Functional annotation analysis obtained by DAVID Software of genes significant downregulated **(Left)** and upregulated **(Right)** upon MSI2KD according to an adjusted P-value <0.2 in the comparison of Z138CT vs Z138MSI2KD GEPs. The most significant terms, gene count, fold enrichment and P-value are indicated.

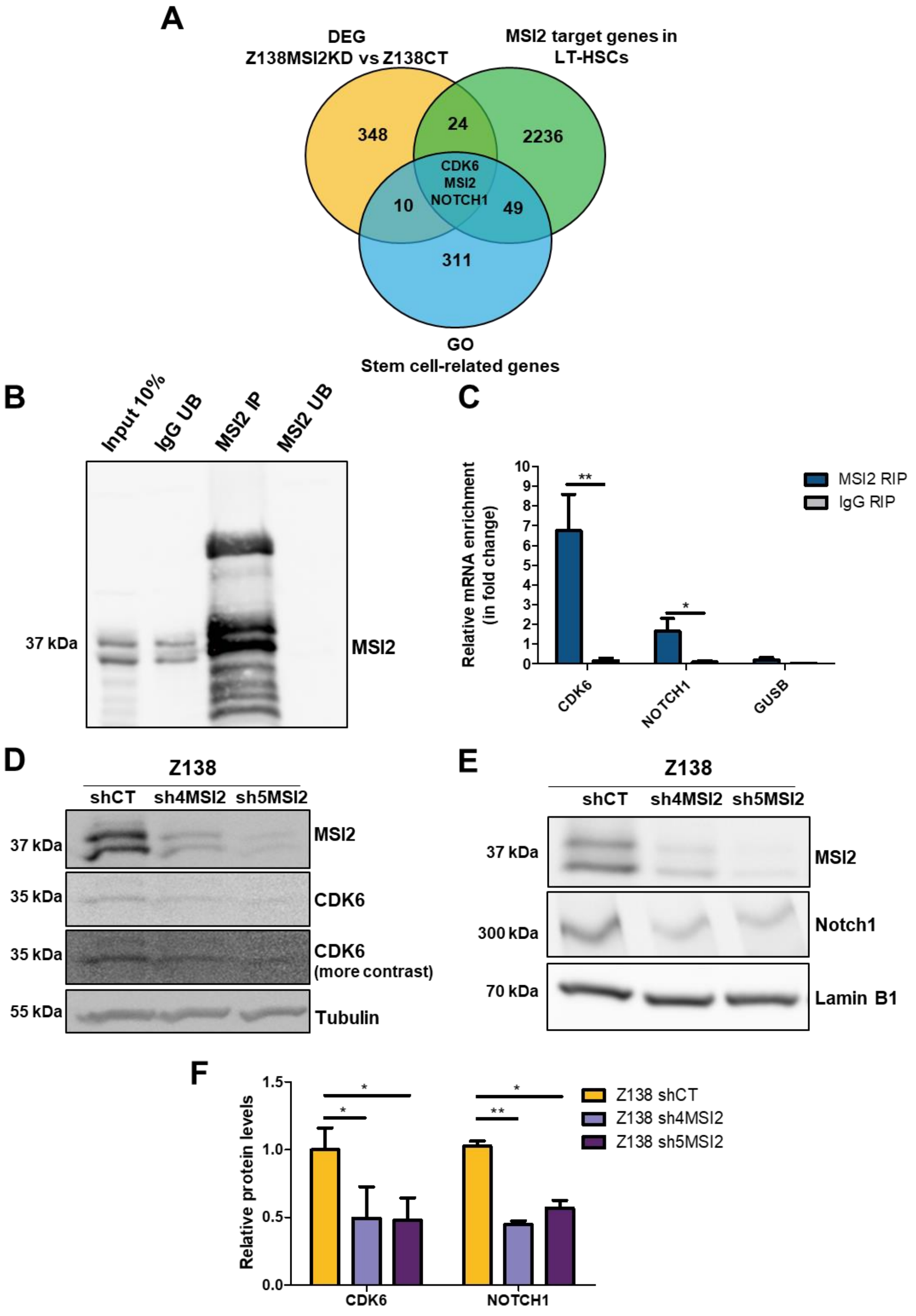

Fig. S6. CDK6 and NOTCH1 mRNAs are direct targets of MSI2 RNA-binding protein in MCL. (A) Venn diagram showing overlapping between DEG after MSI2KD in Z138

MCL cell line (yellow circle, Table S9; 385 genes with Gene Symbol, adjusted P-value <0.1 and absolute log_2-transformed fold change >0.65), previously published direct target mRNAs of MSI2 in long term (LT)-HSCs, found by HyperTRIBE (green circle, 2312 genes) and GO established stem cell-related genes (blue circle, 373 genes; Table S7). **(B)** MSI2-pulldowns from extracts of Z138 MCL cell line (MSI2 IP), immunoprecipitated with a specific anti-MSI2 antibody. The protein fraction not bound to the MSI2 antibody (Unbound MSI2 (MSI2 UB)) is shown. IgG was used as control for RIP showing MSI2 protein in the unbound fraction (IgG UB). **(C)** RT-qPCRs showing CDK6, NOTCH1 and GUSB (used as negative control) mRNA levels in MSI2-specific RIPs (RNA eluted fraction from MSI2-pulldowns (MSI2 IP)) and unspecific IgG RIPs (IgG IP), displayed as fold change relative to their input fraction in 3 independent experiments. **(D-E)** WB experiments showing MSI2 and CDK6 **(D)** and MSI2 and NOTCH1 **(E)** protein levels in Z138shCT, sh4MSI2 and sh5MSI2 cell lines. Tubulin and lamin B1, respectively, were used as loading controls. **(F)** Quantification of CDK6 and NOTCH1 protein levels relative to tubulin or lamin B1 (in fold change) and normalized to Z138shCT, in 3 independent WB experiments. The significance of difference was determined by unpaired two-tailed Student t-test in c and f figures: *P-value <0.05, **P-value <0.01.

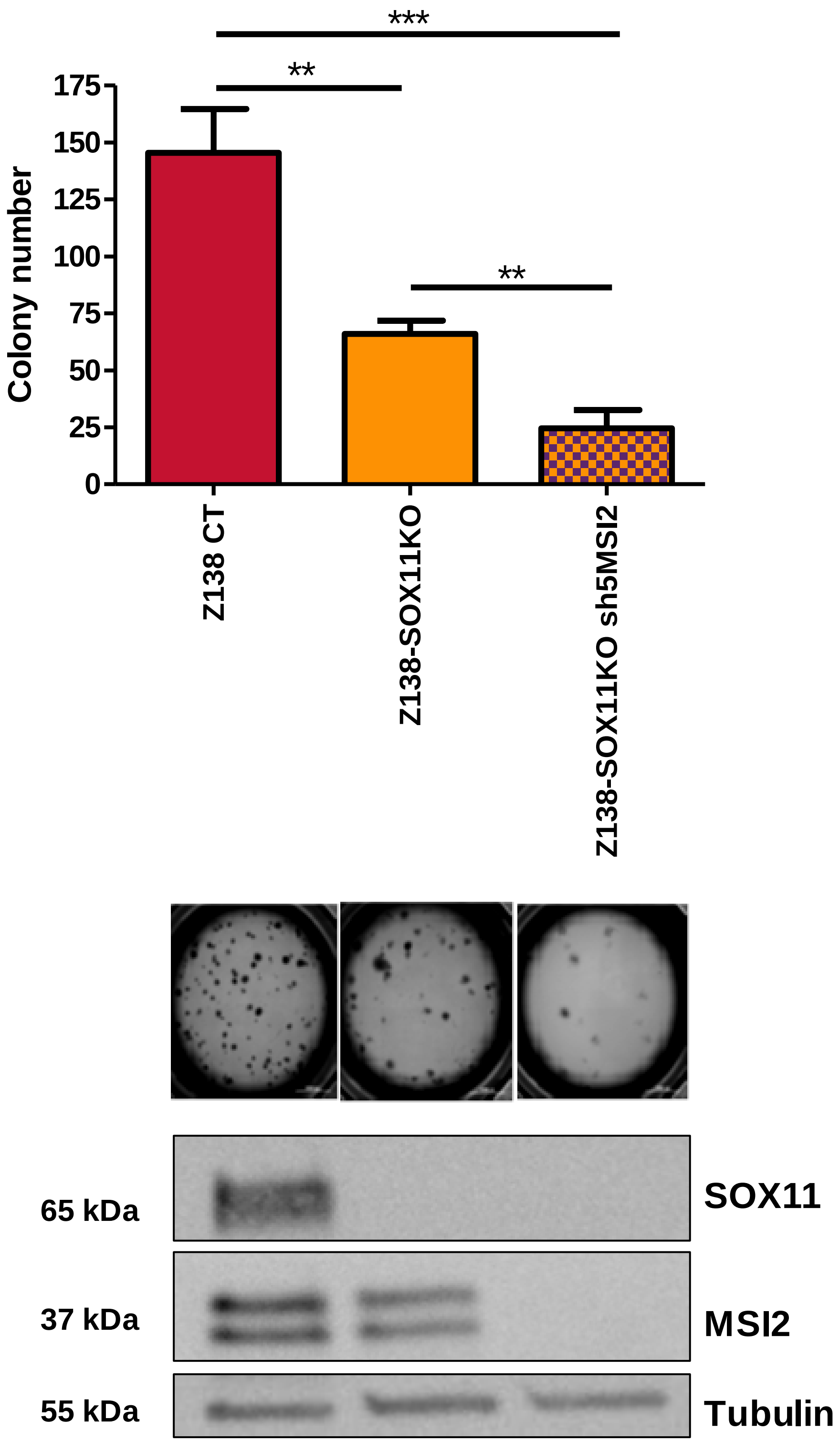

Fig. S7. SOX11KO and MSI2KD reduce colony growth in MCL cells. (Top) Number of colonies counted after 2 weeks of Z138 CT, SOX11KO and SOX11KO sh5MSI2 cells

growing in methylcellulose (1 colony >50 cells). Results are represented as the mean ± standard deviation of 3 independent experiments. **(Mid)** Bright field images of colony assay were obtained using Cytation 5 Imaging Reader with 4X objective lens. To create the full picture 88 images were stitched together (see methods). **(Bottom)** SOX11 and MSI2 protein levels in Z138 CT, SOX11KO and SOX11KO sh5MSI2 cells, obtained by western blot. Tubulin was used as loading control.

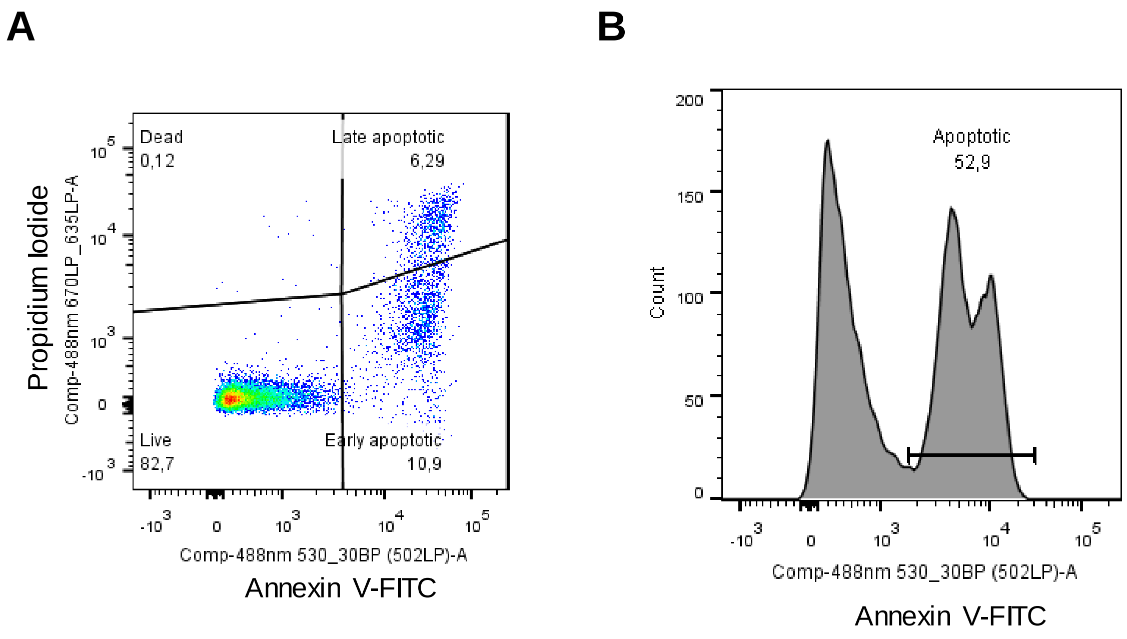

Fig. S8. Gating strategies for cell death analyses. Flow cytometry gating strategy for cell death analyses using Propidium Iodide and Annexin V-FITC **(A)**, or only Annexin V-FITC **(B)** fluorophores.

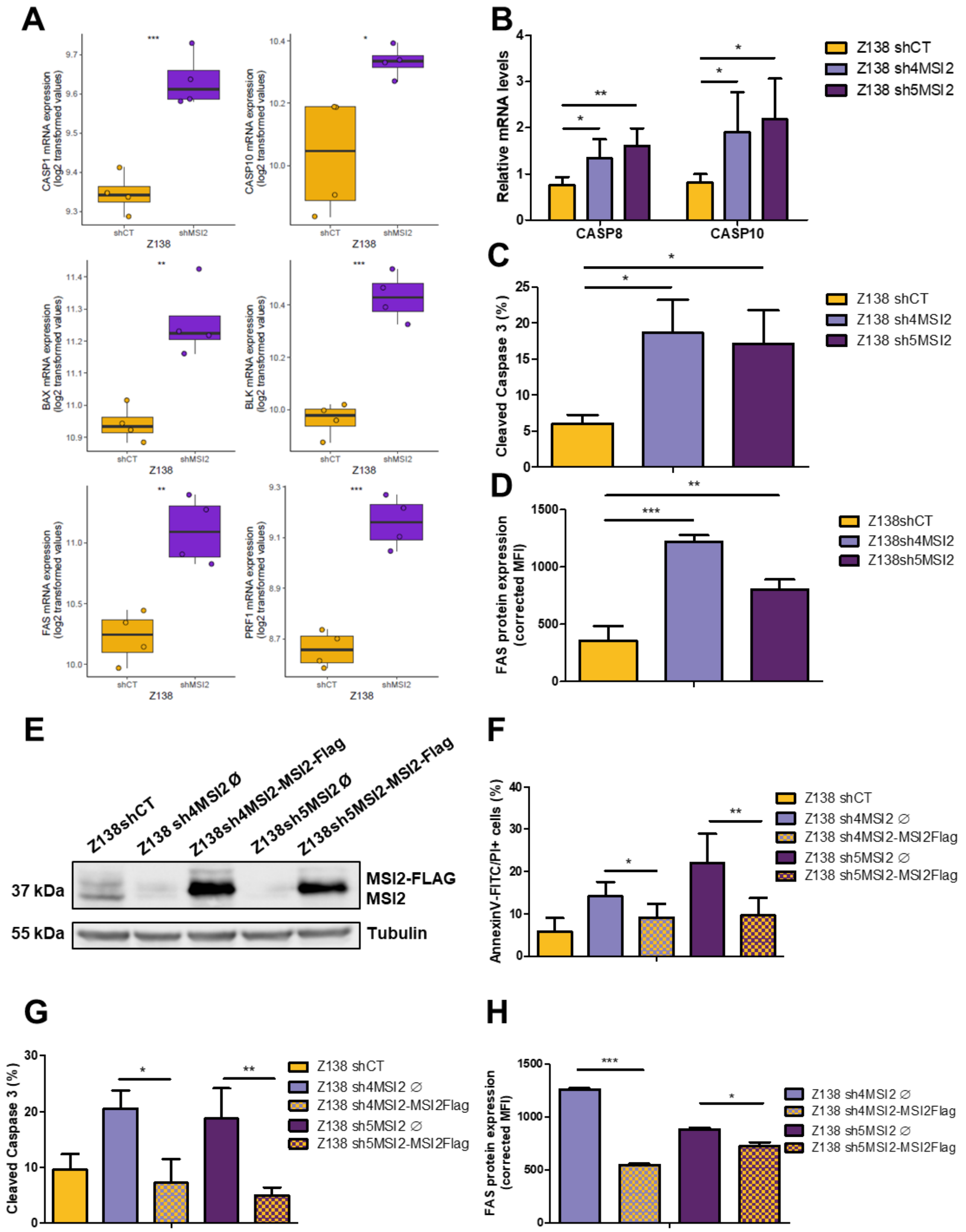

Fig. S9. Apoptosis induced through upregulation of FAS and caspase cascade activation upon MSI2 knockdown is rescued after MSI2 overexpression in MCL cell lines. (A) mRNA expression (in $\log_2$ transformed values) of CASP1, CASP10, BAX, BLK,

FAS and PRF1 in Z138 shCT and shMSI2 cells, obtained by RNA-seq. **(B)** CASP8 and CASP10 mRNA levels, normalized to GUSB endogenous control (in fold change), in Z138 shCT, sh4 and sh5MSI2 cells, obtained by qRT-PCR. The mRNA levels are relative to Z138 shCT. **(C-D)** Cleaved Caspase 3 positive cells (percentage) **(C)** and FAS protein levels **(D)** (corrected mean fluorescence intensity (MFI FAS – MFI isotype)) in Z138 shCT, sh4 and sh5MSI2 cells. **(E)** MSI2-FLAG tagged and MSI2 endogenous protein levels in Z138-shCT, -sh4MSI2 and -sh5MSI2 cells transduced with EF1A empty plasmid (Ø) or EF1A plasmid expressing MSI2-FLAG tagged protein (MSI2-Flag), obtained by western blot. Tubulin was used as loading control. **(F-H)** Apoptotic cells (AnnexinV-FITC/PI positive cells, in percentage) **(F)**, cleaved Caspase 3 positive cells (percentage) **(G)**, and FAS protein levels (corrected mean fluorescence intensity (MFI FAS – MFI isotype)) **(H)** in Z138 shCT cells, and in Z138 sh4MSI2 and sh5MSI2 cells transduced with EF1A empty plasmid (Ø) or EF1A plasmid expressing MSI2-FLAG (MSI2-Flag) tagged protein, obtained by flow cytometry. The significance of difference was determined by unpaired two-tailed Student t-test in at least 3 independent experiments: *P-value <0.05, **P-value <0.01, ***P-value <0.001.

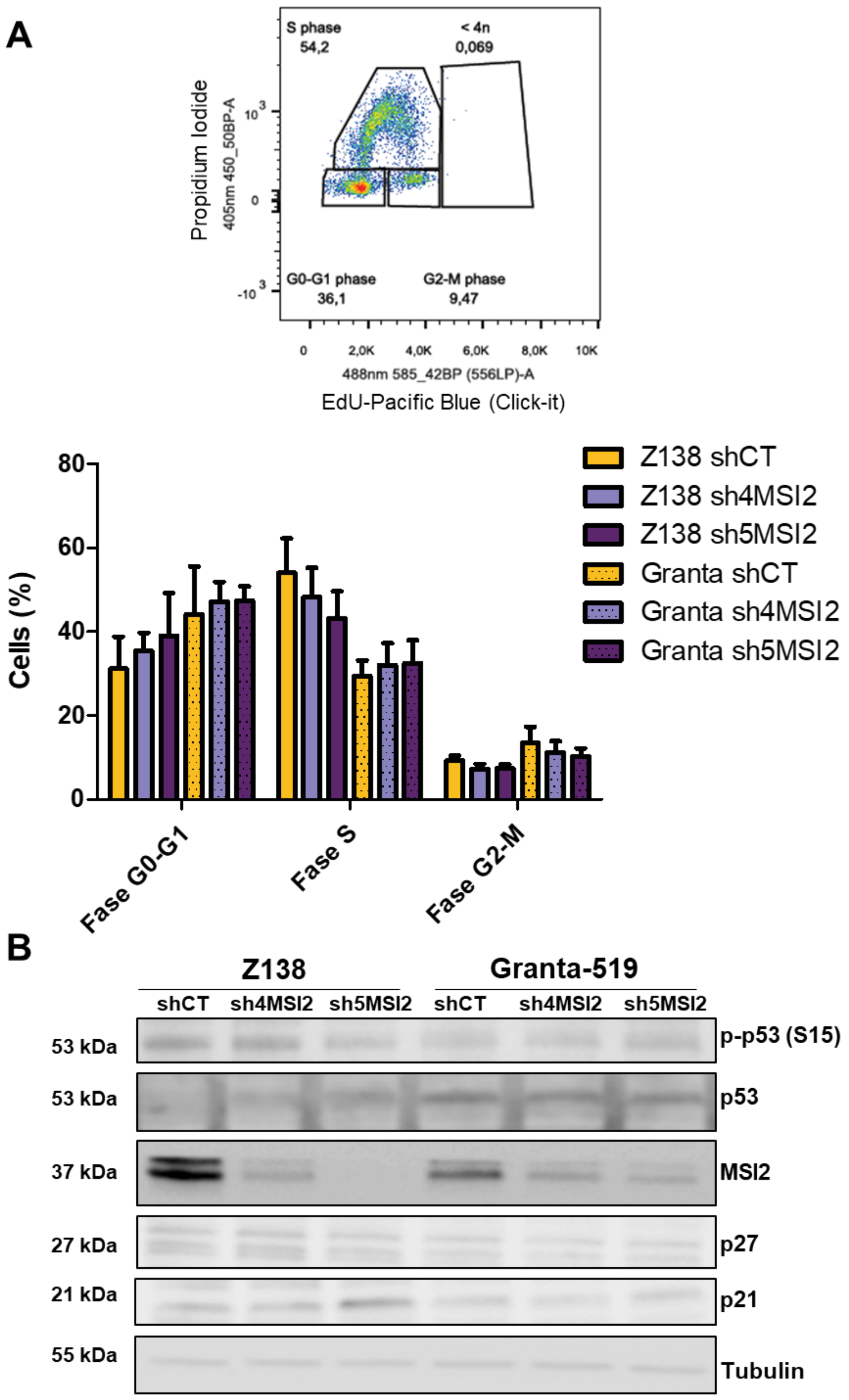

Fig. S10. Cell cycle analyses upon MSI2KD in MCL cell lines. (A) Cell cycle analysis using Propidium Iodide and EdU-Pacific Blue fluorophores for detection of G0-G1, S and

G2-M phases (gating strategy, **Top**) in 3 independent experiments performed in Z138 and Granta MSI2KD and CT cell lines (**Bottom**). Unpaired two-tailed student t-test was used to analyze statistical significance. **(B)** Detection of phospho-p53 (S15), p53, MSI2, p27 and p21 protein levels by western blot in Z138 and Granta MSI2KD and CT cell lines. Tubulin was used as loading control.

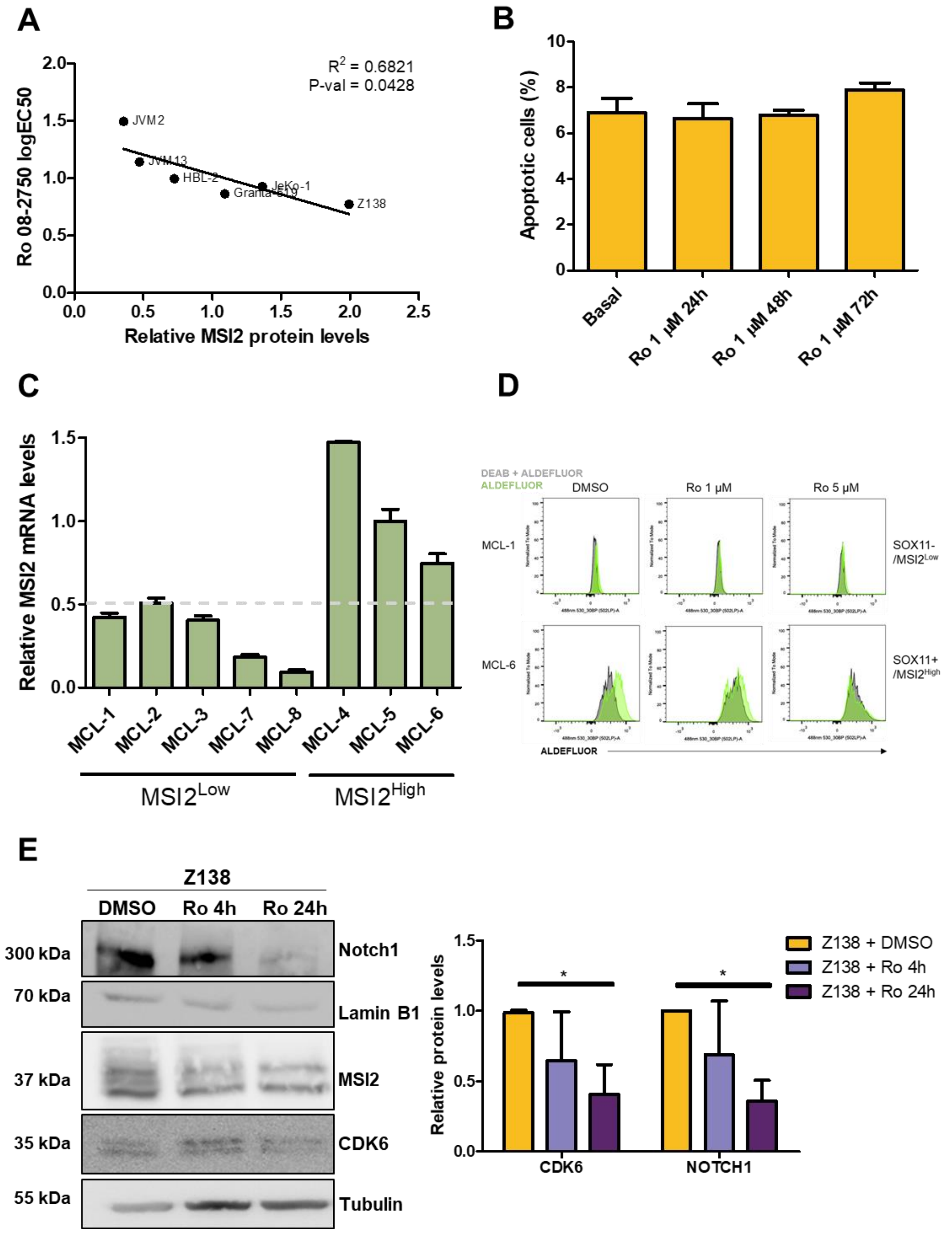

Fig. S11. Effects of MSI2 inhibition with Ro 08-2750 small molecule in MCL. (A) Qualitative correlation between Ro cytotoxicity, measured by the $logEC_{50}$, and relative MSI2 protein levels obtained by western blot (relative to tubulin) in MCL and

lymphoblastic cell lines. Graph shows Pearson coefficient of determination (R^2) and p value (P-val). **(B)** Apoptosis assay showing percentage of Annexin V+ cells in Z138 MCL cell line after 24h, 48h and 72h of Ro treatment at 1 µM. Unpaired two-tailed student t-test was used to analyze statistical significance. **(C)** MSI2 mRNA levels, normalized to GUSB endogenous control (in fold change), in MCL leukemic primary cases (N = 8). The mRNA levels are relative to MCL-5 case. A cutoff at 0.51 (grey line) in relative MSI2 expression was established in order to separate cases with MSI2 high and low expression. **(D)** Histograms showing fluorescence of ALDEFLUOR (in green) and ALDEFLUOR+DEAB (in grey) samples in representative $MSI2^{Low}$ (MCL-1) and $MSI2^{High}$ (MCL-6) MCL primary cases treated with 1 and 5 µM of Ro or 0.05% of DMSO. **(E) (Left)** MSI2, Notch1 and CDK6 protein levels in Z138 cell line after Ro 20 µM treatment for 4 or 24 hours, or DMSO 0.1% solvent treatment for 24 hours. Tubulin and lamin B1 were used as loading controls. **(Right)** Quantification of CDK6 and Notch1 protein levels relative to tubulin and lamin B1, respectively, and normalized to Z138 treated with DMSO 0.1% in 2 independent experiments. The significance of difference was determined by unpaired two-tailed Student t-test: * P-value <0.05.

REFERENCES

1 Balsas P, Palomero J, Eguileor Á, Rodríguez ML, Vegliante MC, Planas-Rigol E et al. SOX11 promotes tumor protective microenvironment interactions through CXCR4 and FAK regulation in mantle cell lymphoma. Blood. 2017; 130: 501–513.

2 Navarro A, Clot G, Martínez-Trillos A, Pinyol M, Jares P, González-Farré B et al. Improved classification of leukemic B-cell lymphoproliferative disorders using a transcriptional and genetic classifier. Haematologica. 2017; 102: 360–363.

3 Nadeu F, Martin-Garcia D, Clot G, Díaz-Navarro A, Duran-Ferrer M, Navarro A et al. Genomic and epigenomic insights into the origin, pathogenesis, and clinical behavior of mantle cell lymphoma subtypes. Blood. 2020; 136: 1419–1432.

4 Navarro A, Clot G, Royo C, Jares P, Hadzidimitriou A, Agathangelidis A et al. Molecular subsets of mantle cell lymphoma defined by the IGHV mutational status and SOX11 expression have distinct biologic and clinical features. Cancer Res. 2012; 72: 5307–5316.

5 Clot G, Jares P, Giné E, Navarro A, Royo C, Pinyol M et al. A gene signature that distinguishes conventional and leukemic nonnodal mantle cell lymphoma helps predict outcome. Blood. 2018; 132: 413–422.

6 Queirós AC, Beekman R, Vilarrasa-Blasi R, Duran-Ferrer M, Clot G, Merkel A et al. Decoding the DNA Methylome of Mantle Cell Lymphoma in the Light of the Entire B Cell Lineage. Cancer Cell. 2016; 30: 806–821.

7 Vilarrasa-Blasi R, Soler-Vila P, Verdaguer-Dot N, Russiñol N, Di Stefano M, Chapaprieta V et al. Dynamics of genome architecture and chromatin function

during human B cell differentiation and neoplastic transformation. Nat Commun. 2021; 12: 1–18.

8 Ernst J, Kellis M. ChromHMM: automating chromatin-state discovery and characterization. Nat Methods. 2012; 9: 215–216.

9 Beekman R, Chapaprieta V, Russiñol N, Vilarrasa-Blasi R, Verdaguer-Dot N, Martens JHA et al. The reference epigenome and regulatory chromatin landscape of chronic lymphocytic leukemia. Nat Med. 2018; 24: 868.

10 Heigwer F, Kerr G, Boutros M. E-CRISP: fast CRISPR target site identification. Nat Methods. 2014; 11: 122–123.
